# Comparative effectiveness of treatment strategies for pediatric empyema thoracis: a systematic review with pairwise and network meta-analyses

**DOI:** 10.3389/fped.2026.1980275

**Published:** 2026-10-26

**Authors:** Minat Allah Alhusami, Khaled Senjab, Maryam Alabdullah, Momina Malik, Sarah Kanj, Lana Kanj, Hussein Naji

**Affiliations:** 1 College of Medicine, Mohammed Bin Rashid University of Medicine and Health Sciences, Dubai Health, Dubai, United Arab Emirates; 2 Department of Pediatric Surgery, Mediclinic Parkview Hospital, Dubai, United Arab Emirates

**Keywords:** chest tube drainage, intrapleural fibrinolysis, network meta-analysis, pairwise meta-analysis, pediatric empyema, systematic review, thoracotomy, video-assisted thoracoscopic surgery (VATS)

## Abstract

**Background:**

Empyema thoracis is a complication of bacterial pneumonia in children with substantial morbidity. Multiple treatment options are available, but the optimal strategy remains uncertain. This systematic review compared major treatment strategies for pediatric empyema thoracis across clinically relevant outcomes.

**Methods:**

The protocol was prospectively registered with PROSPERO (CRD420251274193). PubMed, Embase, Scopus, Web of Science, and CENTRAL were searched for studies published from January 2000 through January 2026 evaluating empyema thoracis in children aged <18 years. Random-effects pairwise and frequentist network meta-analyses compared chest tube (CT) alone, CT + fibrinolytics, video-assisted thoracoscopic surgery (VATS), and thoracotomy. The primary outcome was hospital length of stay (LOS); secondary outcomes included treatment escalation, treatment success, complications, mortality, and treatment-related durations. Treatments were ranked using P-scores; a sensitivity analysis restricted the network to primary-line arms. Risk of bias was assessed using ROBINS-I, RoB 2, or the JBI critical appraisal checklist, as appropriate to study design, and certainty of evidence was evaluated using GRADE.

**Results:**

Seventy-seven studies (7,378 children) were included. Compared with CT, treatment escalation was lower with VATS (RR 0.17, 95% CI 0.07–0.37), thoracotomy (RR 0.13, 95% CI 0.04–0.36), and CT + fibrinolytics (RR 0.48, 95% CI 0.26–0.89); certainty was moderate for three of six escalation comparisons and low or very low elsewhere. VATS was associated with shorter pleural drainage (MD −2.54 days, 95% CI −4.64 to −0.44), antibiotic duration (−4.95, −9.36 to −0.54), and fever duration (−2.00, −3.97 to −0.04) but not with LOS (−2.06, −4.57 to 0.45). In pairwise analysis, escalation was higher with CT + fibrinolytics than with VATS (RR 3.18, 95% CI 1.45–6.96). Restricting the network to primary-line arms preserved these associations; the higher network mortality with thoracotomy lost significance. Exploratory mean P-scores were 0.74 (VATS), 0.50 (thoracotomy), 0.47 (CT + fibrinolytics), and 0.29 (CT alone).

**Conclusions:**

CT alone was associated with more treatment escalation than each alternative, while VATS was associated with shorter treatment-related durations. However, the evidence is predominantly observational, of low to very low certainty for most comparisons, while estimates involving thoracotomy were sensitive to treatment line. These associations are hypothesis-generating and do not establish a treatment hierarchy. Trials with explicit staging and standardized escalation definitions are needed.

**Systematic Review Registration:**

https://www.crd.york.ac.uk/PROSPERO/view/CRD420251274193, PROSPERO CRD420251274193.

## 1 Introduction

Empyema thoracis is an accumulation of purulent fluid within the pleural space, most commonly developing as a complication of bacterial pneumonia in children (1). It is associated with significant morbidity, prolonged hospitalization, and risk of rapid clinical deterioration (2). Pleural infection begins with an exudative parapneumonic effusion, which may advance to a fibrinopurulent stage characterized by infected fluid, loculations, and septation formation, and ultimately to an organizing stage marked by fibroblast proliferation and pleural thickening. Disease staging guides management and influences the effectiveness of different treatment approaches (3, 4).

Management of empyema thoracis is multifaceted, ranging from less invasive measures such as chest tube (CT) drainage with or without intrapleural fibrinolytic therapy, to surgical approaches such as decortication with video-assisted thoracoscopic surgery (VATS) or open thoracotomy. Previous meta-analyses have compared outcomes across treatment strategies for empyema thoracis. Evidence suggests that primary non-operative management, including antibiotics, thoracocentesis, and CT drainage, may be associated with longer hospital stays, higher rates of reintervention, and increased mortality compared with operative or fibrinolytic approaches (5). However, findings across the literature remain inconsistent. A Cochrane review reported no statistically significant difference in mortality rates between patients who received surgical and non-surgical management, although VATS may reduce hospital length of stay (LOS) compared with CT drainage alone (6). Individual studies have also reported high success rates across multiple treatment modalities, including thoracocentesis, CT drainage, fibrinolytic therapy, and surgical intervention, although secondary outcomes such as LOS and duration of CT placement vary considerably (7).

Current clinical guidance reflects this uncertainty. The 2026 clinical practice guideline update from the Infectious Diseases Society of America (IDSA) and Pediatric Infectious Diseases Society (PIDS) recommends pleural drainage for children with moderate parapneumonic effusions accompanied by respiratory distress, large effusions, or documented purulent effusions (8). When drainage is indicated for pneumonia-associated empyema, CT with intrapleural fibrinolytics is suggested over VATS as first-line therapy in most cases, primarily because it is less invasive, less costly, and can often be performed without general anesthesia, whereas VATS is generally reserved for extensive loculations or disease refractory to initial drainage and fibrinolysis (8). This recommendation is conditional and based on very low-certainty evidence (8).

Existing evidence is limited by inconsistent case definitions, heterogeneous study populations, and pairwise comparisons restricted to selected interventions or outcomes, making it difficult to compare the relative effectiveness of all available treatment strategies. A network meta-analysis allows multiple treatment strategies to be compared simultaneously by integrating direct and indirect evidence within a single analytical framework, complementing conventional pairwise comparisons. Unlike previous syntheses, this review restricted inclusion to Stage II or III empyema confirmed by prespecified diagnostic criteria and compared CT alone, CT with fibrinolytics, VATS, and thoracotomy across eight outcomes. Randomized and non-randomized comparative studies were included, with certainty assessed for each pooled estimate. Using pairwise and network meta-analysis, we evaluated hospital LOS and other clinically relevant outcomes in children aged <18 years using randomized and non-randomized evidence.

## 2 Methods

### 2.1 Protocol registration

This systematic review and meta-analysis was conducted in accordance with the methodological guidance of the Cochrane Handbook for Systematic Reviews of Interventions and reported according to the Preferred Reporting Items for Systematic Reviews and Meta-Analyses (PRISMA) 2020 statement and the PRISMA extension for network meta-analyses (9–11). The study protocol was prospectively registered with the International Prospective Register of Systematic Reviews (PROSPERO; registration number: CRD420251274193). The review scope was refined to focus specifically on the comparative effectiveness and safety of treatment modalities for pediatric empyema thoracis; this change was made to provide a more focused and clinically relevant review.

### 2.2 Eligibility criteria

Studies were eligible if they reported original research evaluating the management of empyema thoracis in children aged <18 years. Eligible study designs included randomized controlled trials (RCTs), cohort studies, case–control studies, and case series including >10 patients. Studies were required to provide biochemical, radiological, microbiological, procedural, or surgical confirmation of empyema thoracis and to report at least one eligible treatment modality with an associated clinical outcome. Only studies published in English were included because of feasibility constraints and the lack of translation resources.

The review focused on Stage II (fibrinopurulent) and Stage III (organizing) empyema. Stage I (exudative) disease was defined as a free-flowing, non-loculated sterile parapneumonic effusion with pleural fluid pH >7.30 and glucose >60 mg/dL (>3.3 mmol/L) (6, 12). Stage II disease was characterized by loculated or septated pleural fluid with one or more of the following: pleural fluid pH <7.20, glucose <35 mg/dL (<2.2 mmol/L), lactate dehydrogenase (LDH) > 1000 IU/L, neutrophil predominance, positive Gram stain or bacterial culture, frank pus within the pleural space, echogenic pleural fluid on ultrasonography, or radiological evidence of septations or loculations (6, 12). Stage III disease was characterized by organization of the pleural infection, with fibroblast proliferation, pleural peel formation resulting in lung entrapment, and pus within the pleural space (6, 12). When empyema stage was not explicitly reported, studies were eligible only if the diagnosis fulfilled one or more of the diagnostic criteria used to define Stage II or Stage III disease. Studies containing patients with Stage I disease were included only when outcome data for Stage II and/or Stage III disease could be extracted separately.

Studies were excluded if they exclusively evaluated fungal, tuberculous, post-traumatic, postsurgical, or metastatic empyema; reported only incidence, microbiology, epidemiology, or risk factors without treatment outcomes; or exclusively included Stage I parapneumonic effusions. Case reports, case series with <10 patients, narrative reviews, editorials, preprints, commentaries, and conference abstracts without sufficient outcome data were also excluded. Systematic reviews and meta-analyses were not included in the quantitative synthesis, although their reference lists were screened for additional eligible primary studies.

### 2.3 Search strategy, study selection, and data extraction

A systematic literature search was conducted on 24 January 2026, across PubMed, Embase, Scopus, Web of Science, and the Cochrane Central Register of Controlled Trials (CENTRAL) for studies published from January 2000 through January 2026. The search combined terms relating to empyema thoracis, pediatric populations, and relevant treatment and disease characteristics, and was intentionally broad to maximize sensitivity, with the prespecified eligibility criteria applied afterward during study selection (Supplementary Table S1). Backward citation screening was prespecified in the search strategy of the registered protocol and was performed after database screening by manually screening the reference lists of included studies and previous systematic reviews and meta-analyses. Systematic reviews and meta-analyses themselves were not eligible as units of analysis (Section 2.2).

Titles and abstracts were initially screened against the eligibility criteria by five reviewers, with each record independently assessed by two reviewers. This was followed by full-text assessment of potentially eligible articles, with each full-text article independently evaluated by two reviewers. Disagreements regarding study inclusion were resolved through consensus and, where necessary, consultation with the senior author.

Data were extracted by the same five reviewers using a standardized data extraction form, with data from each included study independently extracted by two reviewers. Extracted data included study characteristics, patient characteristics, treatment modality, and prespecified clinical outcomes. For studies reporting multiple eligible treatment arms, data were extracted separately for each treatment group to preserve treatment-specific outcome estimates. Study investigators were not contacted for missing or unclear data; analyses were based on information available in the published reports and Supplementary Materials. No automation tools were used for data extraction. Information not reported in the original study was recorded as “not reported” (NR). Unclear or ambiguous information and discrepancies in data extraction were discussed with the lead author and principal investigator and resolved by consensus. When the published report did not permit reliable interpretation, the information was recorded as unclear rather than inferred. The extraction sheet is provided in the Supplementary Materials.

### 2.4 Interventions and outcomes

The outcomes of interest were prespecified to compare the clinical effectiveness and safety of the principal management strategies for pediatric empyema thoracis. Treatment modalities were categorized as CT alone, CT with intrapleural fibrinolytics, VATS, and thoracotomy with decortication. These four categories formed the treatment nodes of the network. Arms described as tube thoracostomy were treated as synonymous with chest tube drainage alone. All intrapleural fibrinolytic regimens were pooled into a single node irrespective of agent, dose, or schedule, because the number of arms per individual agent was too small to support separate nodes; the pooled node comprised urokinase, streptokinase, and alteplase regimens. This node is therefore clinically heterogeneous, and the network cannot resolve agent-, dose- or schedule-specific effects. Arms consisting of antibiotics alone, thoracentesis alone, or a step-up protocol that could not be attributed to a single modality were not assigned to any node and did not contribute to the network.

Each treatment arm was also classified by treatment line as primary (the initial pleural intervention), step-up (delivered after failure of a less invasive strategy within the same study) or rescue (delivered for clinical deterioration), using the description in the source report. Arms whose line could not be determined were recorded as unclear. In the principal pairwise and network analyses, arms were assigned to nodes by modality irrespective of treatment line so that a thoracotomy performed after failed drainage and a primary thoracotomy contribute to the same node. This choice compares strategies that may have been applied at different points in the disease course. A sensitivity analysis added during peer review restricted the network to primary-line arms only (Section 2.5).

The primary outcome was hospital LOS, reported in days. Secondary outcomes included escalation or reintervention, complications, mortality, duration of chest tube drainage, antibiotic duration, postintervention fever duration, and treatment success. Treatment escalation or reintervention was defined as the requirement, during the index admission, for a subsequent procedure of greater invasiveness than the intervention to which the arm was assigned, for example, progression from chest tube drainage to fibrinolytic therapy or from either to VATS or to thoracotomy with decortication. Escalation was counted from the assigned intervention onward, as reported by each study. For arms delivered as step-up or rescue procedures, escalation was counted only after the step-up procedure itself. Because these arms had already exhausted less invasive options, they had fewer opportunities for further escalation. Consequently, escalation comparisons involving step-up surgical arms may favor those arms, motivating the primary-line sensitivity analysis. Treatment success was extracted according to the definitions provided by the original studies. Continuous outcomes were synthesized quantitatively where sufficient comparable data were available. All eligible reported results were sought, and treatment-specific data were extracted separately. Data that could not be appropriately pooled were retained for narrative synthesis where relevant.

### 2.5 Sensitivity analyses

Leave-one-out sensitivity analyses were performed for pooled estimates to evaluate the influence of individual studies on the overall effect estimates and statistical heterogeneity. In a second, independent sensitivity analysis, all pooled estimates were refitted using restricted maximum likelihood in place of the DerSimonian–Laird estimator of between-study variance, with Hartung–Knapp confidence intervals and inverse-variance rather than Mantel–Haenszel weighting for dichotomous outcomes, to test whether the findings depended on the choice of variance estimator. Agreement between the two model specifications is reported as concordance in the direction and the statistical significance of each pooled estimate. A third sensitivity analysis, added during peer review and not prespecified in the registered protocol, refitted all eight network meta-analyses using arms classified as only primary-line treatment (Section 2.4). Studies contributed only if at least two of their arms were primary. Step-up, rescue, mixed, and unclear arms were excluded. The network specification was otherwise identical to the principal analysis. Estimates were compared with those from the principal network by direction, statistical significance, and confidence interval width. Where a node was no longer connected, the estimate is reported as not estimable rather than imputed.

### 2.6 Quality assessment

Risk of bias was assessed independently by two reviewers, with disagreements resolved by discussion and, where necessary, by a third reviewer. Each study was appraised with the instrument appropriate to its design. Non-randomized comparative studies were appraised with the ROBINS-I tool across its seven domains: confounding, selection of participants into the study, classification of interventions, deviations from intended interventions, missing data, measurement of outcomes, and selection of the reported result (13). Randomized trials were appraised with the Cochrane risk-of-bias tool for randomized trials (RoB 2) across its five domains and single-arm case series with the Joanna Briggs Institute (JBI) critical appraisal checklist for case series (14, 15). Each domain was rated according to its instrument's signaling questions, and every judgment was recorded together with the supporting text from the source article. The registered protocol did not enumerate confounding domains. For the ROBINS-I appraisal, we designated stage of empyema at presentation as the principal confounder as it is strongly associated with both treatment selection and the clinical outcomes assessed. Organized disease drains more slowly, requires longer antibiotic courses, and more often needs a second procedure. We also considered other determinants of treatment selection, including age, extent of loculation, respiratory status, comorbidity, duration of symptoms before presentation, institutional practice, and calendar period. Because no included study reported estimates adjusted for these prognostic factors, empyema stage remained the principal confounder for ROBINS-I assessment. We defined the target intervention for ROBINS-I as the treatment strategy to which the arm was assigned at the start of pleural management, an estimand based on the treatment assigned at the start of pleural management. We therefore recorded crossover to a more invasive procedure after failure of the assigned strategy as a deviation from intended interventions. Under an alternative, pathway-based estimand, this crossover would be part of the strategy rather than a deviation. The primary-line sensitivity analysis approximates that alternative reading. Studies whose own between-strategy comparison was subject to severity-driven allocation were rated at critical risk of bias for this domain. Single-strategy studies were rated at serious rather than critical risk because they did not make an internal between-treatment comparison. The overall judgment of each study was taken as its worst domain judgment. Study-level judgments were then carried to each outcome-by-comparison cell of the pooled analyses to inform the risk-of-bias domain of the GRADE assessment: a cell was rated at very serious risk of bias where at least half of the contributing studies were at critical risk, at serious risk where any contributing study was at critical risk or at least one was at serious risk, and at low risk where all contributing studies were at low or moderate risk (16). Very serious risk of bias led to a two-level downgrade of certainty and serious risk to a one-level downgrade. Certainty of evidence was rated for every outcome-by-comparison analysis using the GRADE approach across five domains: risk of bias, inconsistency, indirectness, imprecision, and publication bias. Because all comparisons were informed predominantly or exclusively by non-randomized studies appraised with ROBINS-I, each analysis began at high certainty and was downgraded from that starting point, as recommended when ROBINS-I is used. Inconsistency was downgraded one level where *I*^2^ exceeded 50% and two levels where it exceeded 75%; imprecision was downgraded one level where the 95% confidence interval crossed the line of no effect and two levels where it additionally spanned appreciable benefit and appreciable harm; publication bias was downgraded one level where the Egger test was significant. Indirectness was rated not serious throughout, as all included populations, interventions, and outcomes matched the review question directly. Certainty was rated for the pairwise (direct) estimates, which form the basis of the summary-of-findings table. The two network-specific assumptions were handled as follows. Incoherence was assessed by node-splitting and by the design-by-treatment interaction test (Section 2.7). Where either test indicated significant disagreement for an outcome, that outcome's network estimates are flagged in the Results. For these estimates, the certainty of the corresponding direct evidence was treated as the maximum plausible certainty. Because transitivity could not be verified for disease stage and severity, and considering the treatment-line imbalance described in Section 2.4, all network estimates were interpreted with this limitation. This is not applied as a formal downgrade of the pairwise certainty ratings. The network estimates are therefore no more certain than the direct evidence that informs them, and are often less certain. The risk-of-bias domain judgments for each pooled analysis are reported in Supplementary Table S6 and the resulting certainty ratings in Supplementary Table S7.

### 2.7 Statistical analysis

Pairwise random-effects meta-analyses were conducted to compare the four principal management strategies: CT, CT with fibrinolytics, VATS, and thoracotomy/decortication. Each eligible pair of interventions was analyzed separately for each outcome. For binary outcomes, treatment effects were expressed as risk ratios (RRs), while continuous outcomes were summarized using mean differences (MDs), with corresponding 95% confidence intervals (CIs). When continuous variables were reported as medians with ranges or interquartile ranges, means were estimated using the method described by Luo et al. and standard deviations using the method described by Wan et al. (17, 18). Studies reporting means without an accompanying measure of dispersion were excluded from the corresponding pooled estimate without imputation of the standard deviation. When a study contributed more than one eligible intervention arm to the same treatment category, the relevant groups were combined according to the methods recommended in the Cochrane Handbook before inclusion in the meta-analysis (9). A pairwise meta-analysis was undertaken only when at least two studies directly compared the relevant interventions. Outcomes supported by a single study were summarized narratively. Statistical heterogeneity was assessed using the *I*^2^ statistic and between-study variance (*τ*^2^). Leave-one-out analyses were used to assess the robustness of pooled estimates and identify influential studies. The pairwise meta-analysis results were visually presented using forest plots.

A frequentist random-effects network meta-analysis was additionally performed to integrate direct and indirect evidence across the four treatment modalities. Only studies reporting two or more of the four treatment modalities were eligible for the network, as a contrast-based model requires a within-study comparison; single-arm studies were therefore excluded from the network analyses and summarized narratively. Treatment ranking was assessed using P-scores, ranging from 0 to 1, with higher values indicating a more favorable relative ranking for the corresponding outcome, and were displayed using rankograms. The direction of benefit was specified separately for each outcome before ranking. Lower values were treated as favorable for all four duration outcomes, mortality, complications, and escalation. Higher values were treated as favorable for treatment success. P-scores were computed and are reported per outcome. An unweighted mean of the eight outcome-specific P-scores is also reported. No outcome weighting or clinical prioritization was prespecified. Because the eight outcomes differ in clinical importance and in susceptibility to bias, this mean is presented as an exploratory summary of the rankings and not as a measure of overall treatment effectiveness. Studies contributing three or more eligible arms were included as multiarm studies, with the correlation between their contrasts accounted for by the multivariate approach implemented in netmeta. Network geometry was examined using network plots depicting the available direct comparisons among treatments. The transitivity assumption was assessed clinically by comparing the distribution of potential effect modifiers across the six direct comparisons. These included study design, publication period, care setting (single- versus multicenter), WHO region, the proportion of studies reporting empyema stage, and, following peer review, the proportion of contributing arms delivered as primary versus step-up or rescue treatment (Supplementary Table S9). Empyema stage and markers of disease severity were reported too inconsistently to permit a formal comparison of their distribution across comparisons, and the transitivity assumption could therefore not be verified with respect to the effect modifiers of greatest concern. Network connectivity permitted indirect estimation but did not establish clinical exchangeability across comparisons; transitivity was therefore considered unverified (Sections 2.6 and 4.1). Agreement between direct and indirect evidence was evaluated using node-splitting analyses, while global network consistency was assessed using the design-by-treatment interaction model. The registered protocol prespecified random-effects models for all meta-analyses. The random-effects design-by-treatment interaction test is therefore the prespecified inconsistency assessment. We chose this test because between-study heterogeneity was expected to be substantial for the duration outcomes. In that setting, the fixed-effect test attributes heterogeneity to inconsistency and inflates the rate of significant findings. The fixed-effect test was run as a *post hoc* check, and both results are reported. When the models disagreed, interpretation was based on the prespecified random-effects test because the fixed-effect model does not account for between-study heterogeneity. Potential publication bias and small-study effects were evaluated using funnel plots and Egger's regression test for outcomes with sufficient contributing studies (19). Comparison-adjusted funnel plots were additionally examined for outcomes included in the network meta-analysis.

All statistical analyses were performed employing R version 4.5.3 (R Foundation for Statistical Computing, Vienna, Austria), using the meta, netmeta, and metafor packages (20).

## 3 Results

### 3.1 Study selection and characteristics

The systematic search identified 2,138 records, of which 1,247 remained after duplicate removal (Figure 1). Following title and abstract screening and full-text assessment of 301 studies, 77 met the eligibility criteria and were included in the review, together enrolling 7,378 children with empyema thoracis (7, 21–96). Five were RCTs (33, 58, 60, 84, 89), 68 were observational cohort studies (7, 21–23, 25–32, 34–46, 48, 50–57, 59, 61–68, 70–83, 85–88, 90–95) (retrospective or prospective), and four were case series (24, 47, 49, 69). The included studies spanned 23 countries, with India (18 studies) (21, 27, 35, 36, 44, 54, 55, 61, 63–65, 68, 70, 75, 81, 84, 87, 88), Turkey (14 studies) (7, 24, 25, 33, 38–40, 43, 51, 66, 67, 78, 92, 93) and the US (11 studies) (29, 31, 41, 42, 49, 53, 62, 72, 82, 89, 91) contributing the most, and the great majority were conducted at single centers (69 of 77) (21–37, 39–44, 46–59, 61–82, 84, 85, 87–96). Median sample size was 60 patients per study (range 12–681). Among the 66 studies reporting sex, the median proportion of boys was 55.0%. Empyema stage was reported in only 25 of the 77 studies (32.5%), confirming that a subgroup analysis by stage was not feasible (22, 24, 25, 28, 29, 31–33, 39, 44, 47, 48, 57, 66, 68, 72, 73, 76, 79, 83, 84, 87, 88, 91, 93). Publication dates ranged from 2000 to 2026. CT was the most commonly reported treatment, followed by CT with fibrinolytics, VATS, and thoracotomy (Supplementary Table S2). Potentially relevant studies that otherwise appeared to meet the eligibility criteria but were excluded following full-text assessment are presented in Supplementary Table S3 (100, 106, 107), together with the principal reason for exclusion. The most frequent reasons among these studies were failure to meet the prespecified diagnostic criteria for Stage II/III empyema and inability to extract treatment-specific outcome data.

**Figure 1:**
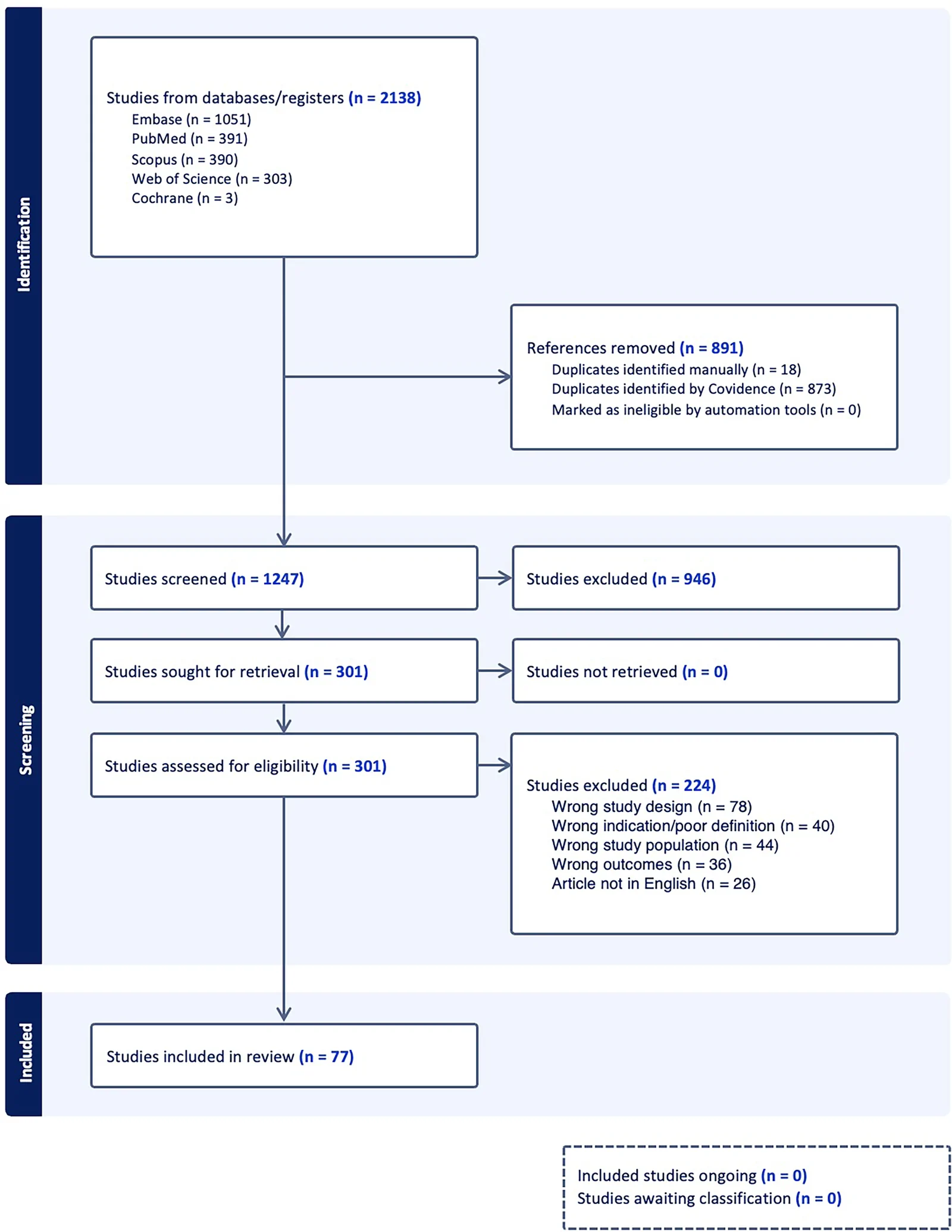
A PRISMA flow diagram illustrating study identification, screening, eligibility, and inclusion.

Three nested sets of studies underlie the results. All 77 included studies contributed at least one treatment arm to the descriptive synthesis of treatment use, microbiology, and complication subtypes (Tables 1, 2). Forty studies reported two or more of the four treatments and contributed within-study contrasts. These 40 studies contributed to both the pairwise meta-analyses and the network, because the contrast-based network model uses the same within-study comparisons. The remaining 37 studies were single-arm reports or included only treatment arms beyond the scope of this review and contributed to the descriptive synthesis only. The correspondence between study design, contribution to the analyses, and risk-of-bias instrument is tabulated in Supplementary Table S10. Four treatments were represented across studies: CT (33 studies) (7, 23, 25–27, 31, 32, 34, 36–38, 44, 45, 49, 50, 54, 55, 59, 61, 63–66, 72, 74, 75, 80, 81, 83, 86, 88, 95, 96), CT with fibrinolytics (34 studies) (25, 32, 33, 35, 36, 38, 39, 42, 43, 45, 46, 50, 51, 57, 58, 60–64, 66, 67, 74–76, 78, 79, 83, 84, 89, 90, 93, 94, 96), VATS (39 studies) (24, 26, 28–30, 32–35, 37, 38, 40, 42, 46, 47, 49, 50, 52, 53, 56, 57, 60, 62, 65, 69–74, 76, 77, 82, 84, 88, 89, 91, 92, 94), and thoracotomy (23 studies) (21–23, 26, 27, 32, 36, 38, 40, 43–45, 48, 50, 59, 65, 66, 68, 77, 78, 81, 85, 87). The 40 comparative studies comprised 35 observational cohorts, four randomized trials, and one case series that reported two treatment groups (Kercher 2000). The fifth randomized trial contributed single-arm data only, because its comparator was not one of the four network treatments (Supplementary Tables S2 and S10).

**Table 1:** Microbiology and geographic distribution.

Characteristic	Value
Studies contributing microbiology data, *n*	58
Studies with total cultures + positivity, *n*	45
Overall culture-positivity rate	42.5%
Organisms isolated	
* Streptococcus pneumoniae*	667 isolates (45 studies)
* Staphylococcus aureus*	592 isolates (37 studies)
* Streptococcus pyogenes*	62 isolates (10 studies)
* *MRSA	42 isolates (10 studies)
WHO region (all 77 studies), *n*	
* *European Region	34
* *South-East Asia Region	19
* *Region of the Americas	16
* *Western Pacific Region	5
* *Eastern Mediterranean Region	2
* *African Region	1

Microbiology denominator is the 58 studies contributing organism or culture data; culture positivity is across the 45 reporting both total and positive cultures. The WHO region is tabulated across all 77 studies. Isolate counts pooled across reporting studies.

**Table 2:** Treatment utilization and complication subtypes, by modality.

Characteristic	CT alone	CT + fibrinolytics	VATS	Thoracotomy/decortication
Utilization				
Studies contributing, *n*	34	35	39	23
Treatment arms, *n*	34	37	47	27
Pooled patients, *n*	1,829	1,554	1,540	856
Complication subtype, *n* (%)				
Surgical-site infection	16 (0.9)	4 (0.3)	2 (0.1)	21 (2.5)
Air leak	0 (0.0)	0 (0.0)	45 (2.9)	14 (1.6)
Subcutaneous emphysema	0 (0.0)	3 (0.2)	33 (2.1)	0 (0.0)
Pneumatocele	0 (0.0)	0 (0.0)	13 (0.8)	0 (0.0)
Pneumothorax	3 (0.2)	7 (0.5)	15 (1.0)	0 (0.0)

Pooled patients sum across all arms of each modality; a multiarm study contributes to more than one column. Complications were inconsistently reported, and therefore, percentages are minimum estimates. Studies=studies contributing that modality to the network. Across all arms with treatment-line data (*n* = 145), 33 (23%) were delivered as step-up or rescue therapy.

Six pairwise comparisons were possible across eight outcomes: LOS, pleural drainage duration, duration of antibiotic therapy, postintervention fever duration, treatment success, need for treatment escalation, complications, and mortality. The definition of treatment success was not uniform: some studies required resolution of symptoms without further surgery, while others used radiographic clearance or simply avoided a second procedure (Supplementary Table S4).

Microbiological data were available from 58 studies. Among the 45 studies reporting both total cultures and positivity, the overall culture-positivity rate was 42.5%. *Streptococcus pneumoniae* was the most frequently isolated organism (667 isolates across 45 studies) (7, 22–26, 29, 31–34, 36–41, 43, 45–50, 52, 53, 55, 58, 59, 62, 63, 65, 66, 69, 72–74, 76, 77, 83, 89, 91, 95, 96), followed by *Staphylococcus aureus* (592 isolates across 37 studies) (7, 22, 23, 25, 31, 33, 34, 36, 38, 40, 41, 43–48, 50, 52, 54, 58, 59, 62–67, 72, 76, 83, 89, 91, 93, 95, 96), *and Streptococcus pyogenes* (62 isolates across 10 studies) (26, 43, 45, 46, 50, 53, 58, 76, 83, 91). Methicillin-resistant *S. aureus* accounted for 42 isolates across 10 studies (29, 33, 41, 44, 47, 58, 64, 65, 89, 91). The studies were distributed across six World Health Organization regions, most heavily the European Region (34 studies) (7, 22, 24, 25, 28, 33, 34, 37–40, 43, 45, 46, 50, 51, 56, 57, 60, 66, 67, 69, 73, 74, 76–78, 80, 83, 85, 86, 92–94), the South-East Asia Region (19 studies) (21, 27, 35, 36, 44, 48, 54, 55, 61, 63–65, 68, 70, 75, 81, 84, 87, 88), and the Region of the Americas (16 studies) (26, 29, 31, 41, 42, 49, 52, 53, 58, 59, 62, 71, 72, 82, 89, 91) (Table 1).

CT alone was used exclusively as a primary therapy, whereas thoracotomy was often employed as a step-up procedure (33 of 145 treatment arms were step-up or rescue) (22, 23, 27, 28, 32, 36, 38, 43, 44, 47, 48, 52, 57, 58, 62, 65, 66, 68, 70, 79, 84, 88). Among the 94 study-by-treatment units that entered the network, 68 were primary line, 17 were step-up or rescue, four pooled primary and step-up patients within a single treatment node, and five could not be classified from the source report (Supplementary Table S11). The imbalance was concentrated in the thoracotomy comparisons. Primary-line status applied to 87%–100% of CT arms, 38%–80% of CT with fibrinolytics arms, and 62%–75% of VATS arms but only 21%–38% of thoracotomy arms (Supplementary Table S9). Among complication subtypes, air leak was most common after VATS (2.9%), while surgical-site infection was most frequent after thoracotomy (2.5%) and CT alone (0.9%) (Table 2).

### 3.2 Pairwise meta-analysis

#### 3.2.1 CT with fibrinolytics versus VATS

This comparison had the largest evidence base, with up to 12 studies contributing to a single outcome. VATS was associated with higher treatment success, shorter drainage duration, shorter fever duration, and a lower risk of escalation; no other outcome reached statistical significance. Treatment success was modestly but consistently higher after VATS (RR 0.91 for CT with fibrinolytics relative to VATS, 95% CI 0.85–0.98, *p* = 0.010; *I*^2^ = 36%; 12 studies, 813 children) (Figure 2A), and the finding was consistent when the single largest cohort was removed in the leave-one-out check. Drainage duration was shorter with VATS (MD 1.11 days in favor of VATS, 95% CI 0.88–1.35, *p* < 0.001; *I*^2^ = 0%; three studies) (Figure 2B), and children given fibrinolytics were about three times as likely to require escalation to a further procedure (RR 3.18, 95% CI 1.45–6.96, *p* = 0.004; *I*^2^ = 25%; nine studies, 687 children) (Figure 2C). This was the most robust of the three associations and remained unchanged on leave-one-out and in the alternative-estimator sensitivity analysis. Postintervention fever duration was approximately 1 day shorter after VATS (MD 0.95 days, 95% CI 0.02–1.89, *p* = 0.046; *I*^2^ = 0%; four studies) (Figure 2D).

**Figure 2:**
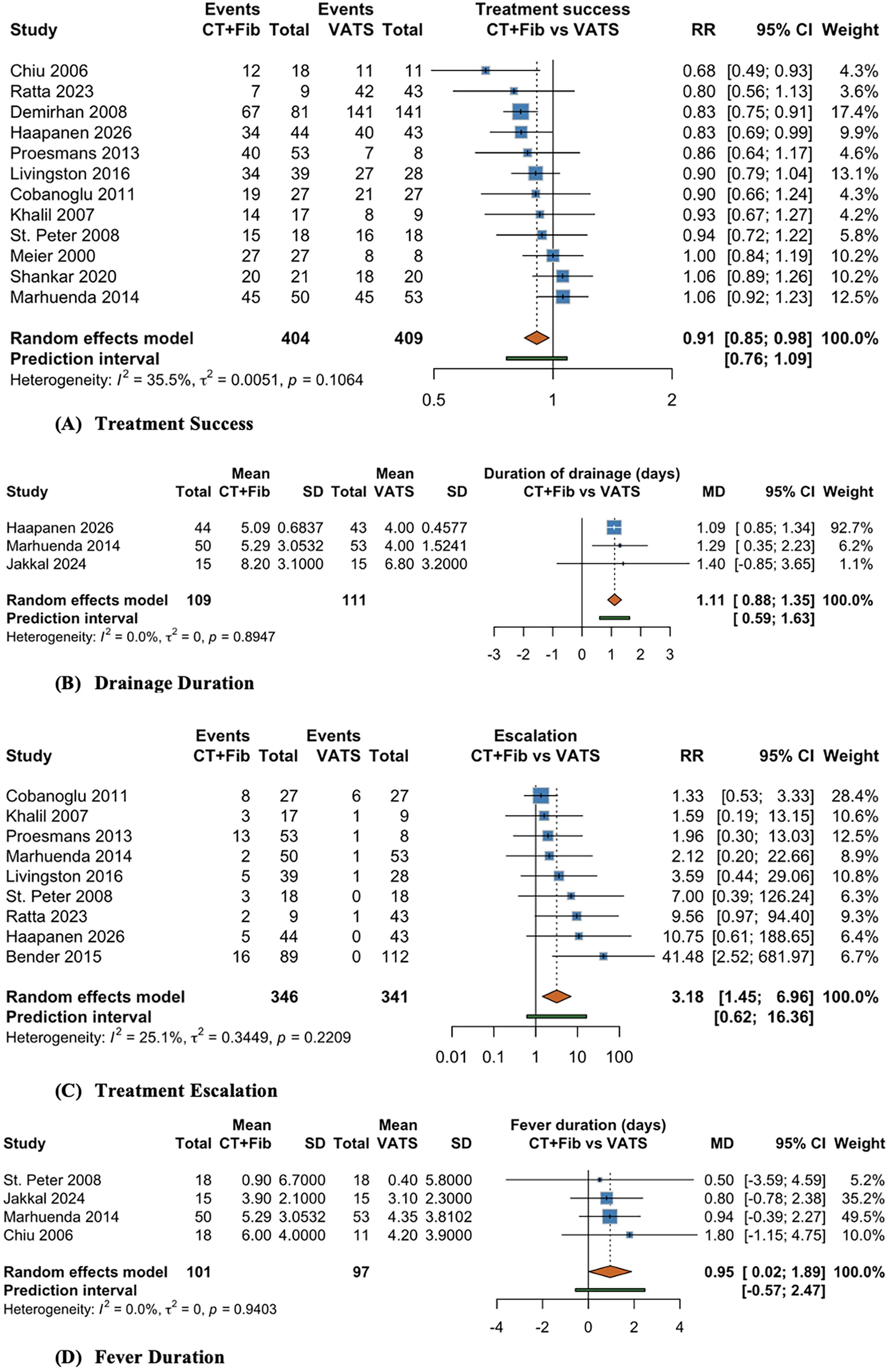
Pairwise meta-analysis comparing chest tube drainage with fibrinolytics (CT + fib) and video-assisted thoracoscopic surgery (VATS). **(A)** Treatment success; **(B)** drainage duration; **(C)** treatment escalation; **(D)** fever duration.

No statistically significant difference was detected for the remaining outcomes: length of stay (MD 0.46 days, 95% CI −1.24 to 2.16, *p* = 0.60; *I*^2^ = 84%), antibiotic duration (MD 0.30 days, 95% CI −0.13 to 0.73, *p* = 0.17; *I*^2^ = 0%), and complications (RR 1.15, 95% CI 0.72–1.82, *p* = 0.56; *I*^2^ = 0%). Mortality could not be compared, as the contributing studies recorded essentially no deaths in either arm.

#### 3.2.2 CT with fibrinolytics versus CT alone

Adding fibrinolytics to CT roughly halved the need for escalation (RR 0.40, 95% CI 0.20–0.79, *p* = 0.008; *I*^2^ = 72%; eight studies, 679 children) (Figure 3). The association remained significant in the leave-one-out and alternative-estimator sensitivity analyses, although its statistical heterogeneity was substantial.

**Figure 3:**
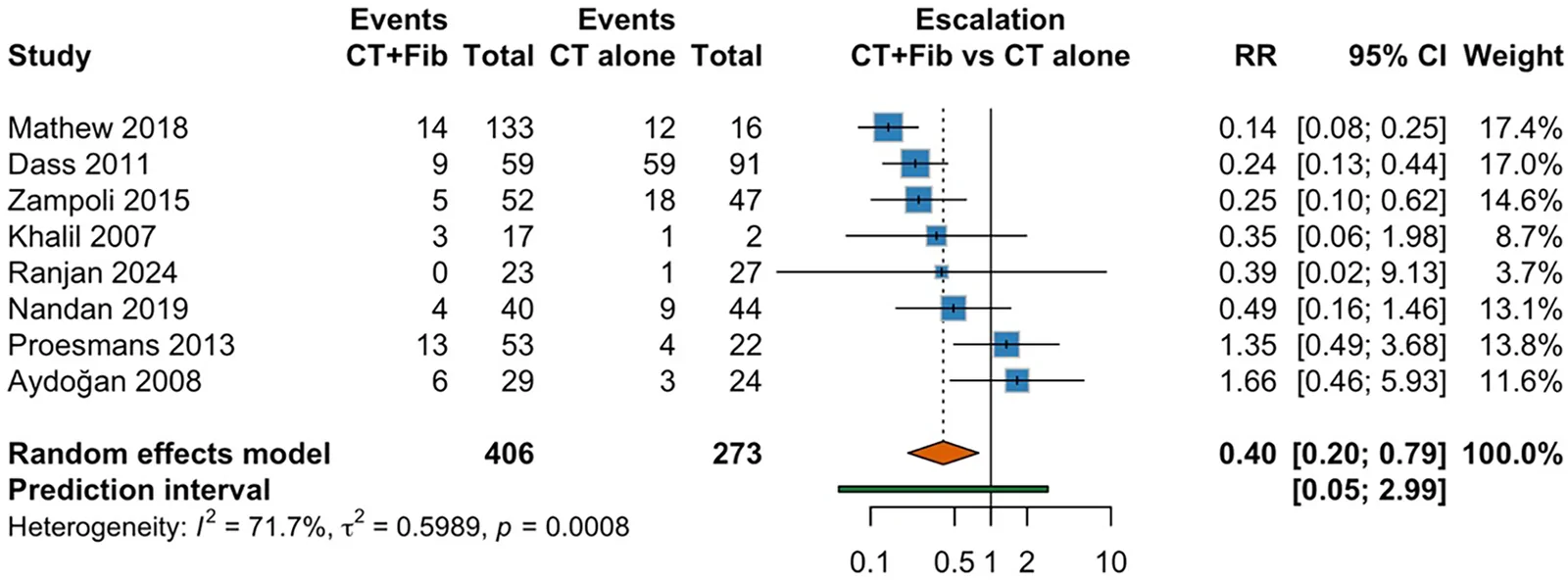
A forest plot comparing treatment escalation between chest tube drainage alone (CT) and chest tube drainage with fibrinolytics (CT + fib).

No statistically significant difference was detected for any other outcome, and the wide intervals do not establish equivalence. Length of stay (MD −1.33 days, 95% CI −4.36 to 1.70, *p* = 0.39; *I*^2^ = 88%), drainage duration (MD −0.74 days, 95% CI −3.20 to 1.72, *p* = 0.55; *I*^2^ = 83%), fever (MD −1.62 days, 95% CI −5.68 to 2.43, *p* = 0.43; *I*^2^ = 91%), antibiotic duration (MD −1.97 days, 95% CI −6.79 to 2.85, *p* = 0.42; *I*^2^ = 84%), treatment success (RR 1.01, 95% CI 0.88–1.15, *p* = 0.93; *I*^2^ = 82%), complications (RR 2.04, 95% CI 0.87–4.81, *p* = 0.10; *I*^2^ = 74%), and mortality (RR 1.42, 95% CI 0.37–5.41, *p* = 0.61; *I*^2^ = 29%) were all non-significant. The treatment-success estimate showed evidence of small-study asymmetry on formal testing (Egger *p* = 0.003), a caveat to its null result.

#### 3.2.3 CT alone versus thoracotomy/decortication

CT alone was associated with a higher risk of escalation than thoracotomy (RR 5.96, 95% CI 1.48–23.95, *p* = 0.012; *I*^2^ = 0%; four studies) (Figure 4A). However, the estimate was imprecise, with a wide confidence interval and few events (no escalations at all in the surgical arms). The mortality estimate favored CT alone but was similarly imprecise. Deaths were more frequent in the thoracotomy arm (RR for CT alone 0.17, 95% CI 0.04–0.84, *p* = 0.030; *I*^2^ = 9%; 4 informative studies) (Figure 4B), but this rested on just 7 deaths in total, with 3 among 207 patients with CT versus 4 among 36 children, who had undergone thoracotomy, from four informative studies.

**Figure 4:**
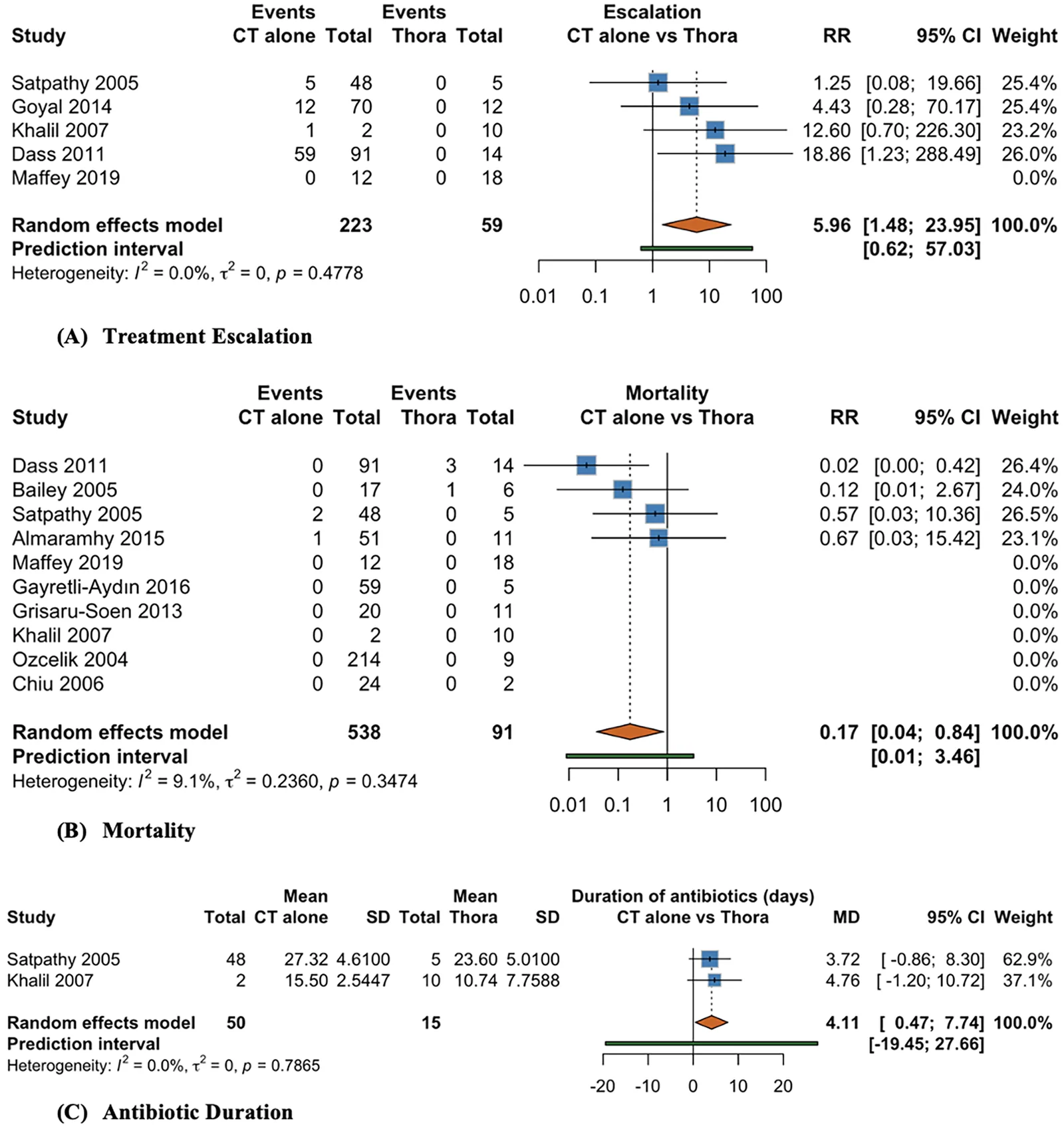
A pairwise meta-analysis comparing chest tube drainage alone (CT) and thoracotomy with decortication (Thora). **(A)** Treatment escalation; **(B)** mortality; **(C)** antibiotic duration.

The duration of antibiotics was longer with CT alone (MD 4.11 days, 95% CI 0.47–7.74, *p* = 0.027; *I*^2^ = 0%), although this estimate was based on only two small studies (Figure 4C). LOS (MD −1.42 days, 95% CI −5.31 to 2.48, *p* = 0.48; *I*^2^ = 92%), drainage (MD −3.61 days, 95% CI −12.38 to 5.16, *p* = 0.42; *I*^2^ = 88%), treatment success (RR 0.92, 95% CI 0.84–1.01, *p* = 0.07; *I*^2^ = 85%), and complications (RR 1.30, 95% CI 0.67–2.50, *p* = 0.43; *I*^2^ = 0%) showed no statistically significant difference. Given the width of these intervals, the results do not demonstrate equivalence.

#### 3.2.4 CT alone versus VATS

The clearest difference was observed for treatment escalation. CT alone carried nearly five times the escalation risk of VATS (RR 4.81, 95% CI 1.55–14.91, *p* = 0.007; *I*^2^ = 32%; seven studies, 244 children) (Figure 5A). This association remained significant in both sensitivity analyses. Fever lasted nearly three days longer with CT alone, but this estimate rested on very heterogeneous data (MD 2.96 days, 95% CI 0.09–5.83, *p* = 0.043; *I*^2^ = 95%) (Figure 5B). All other duration outcomes were longer with CT alone as well, but they did not reach statistical significance because of the wide between-study scatter: drainage (MD 3.02 days, 95% CI −4.75 to 10.78, *p* = 0.45; *I*^2^ = 92%) (Figure 5C) and antibiotics (MD 5.60 days, 95% CI −0.84 to 12.03, *p* = 0.088; *I*^2^ = 85%) (Figure 5D). LOS (MD 2.53 days, 95% CI −1.01 to 6.07, *p* = 0.16; *I*^2^ = 88%), treatment success (RR 0.61, 95% CI 0.21–1.74, *p* = 0.36; *I*^2^ = 99%), and complications (RR 1.76, 95% CI 0.49–6.29, *p* = 0.39; *I*^2^ = 0%) showed no statistically significant difference. Mortality could not be estimated.

**Figure 5:**
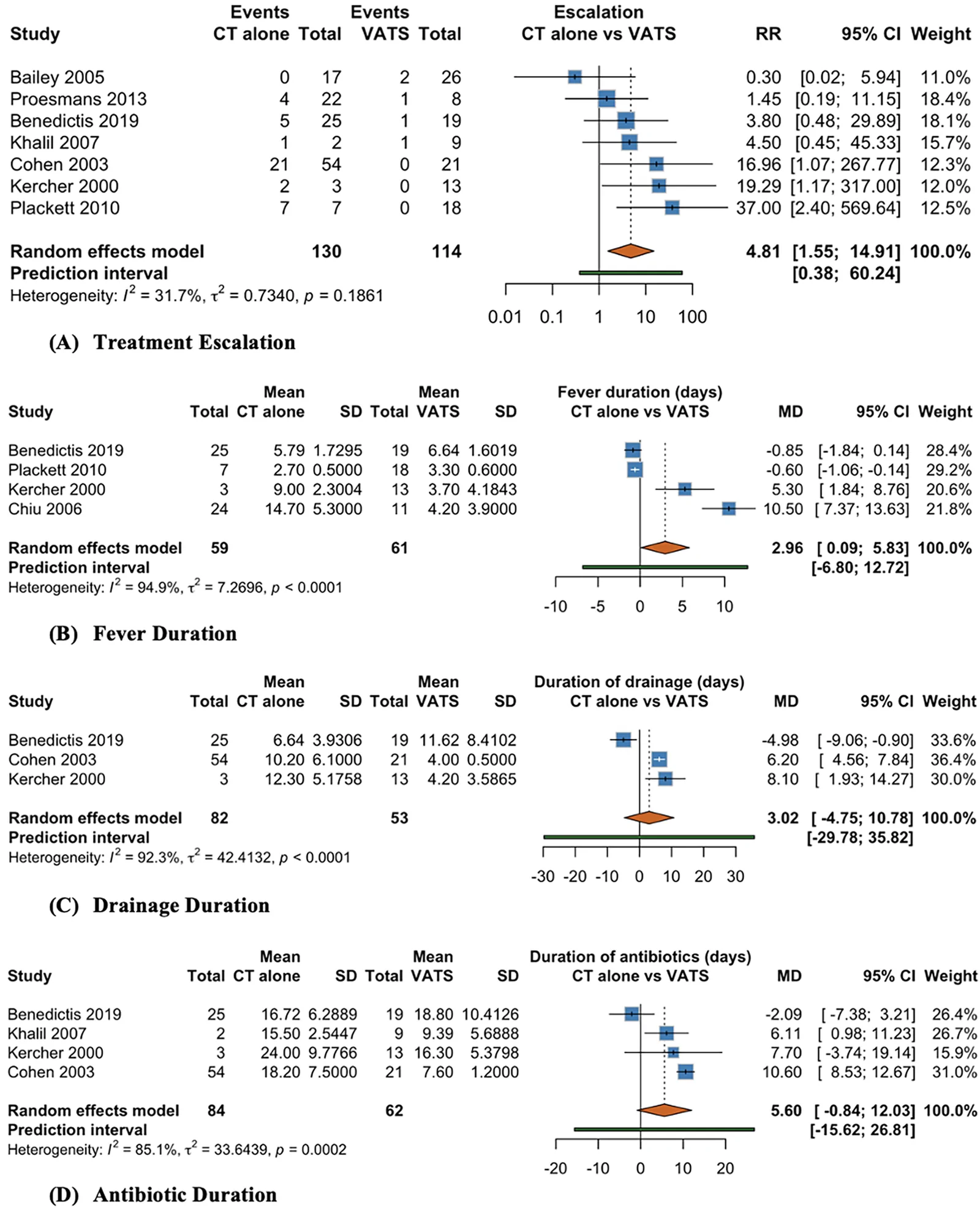
A pairwise meta-analysis comparing chest tube drainage alone (CT) and video-assisted thoracoscopic surgery (VATS). **(A)** Treatment escalation; **(B)** fever duration; **(C)** drainage duration; **(D)** antibiotic duration.

#### 3.2.5 Thoracotomy/decortication versus VATS

Few studies compared the two surgical approaches directly, and among those that did, no statistically significant difference was detected for most outcomes. The confidence intervals were wide enough to be consistent with clinically important differences in either direction. Non-significant outcomes included LOS (MD 2.33 days, 95% CI −0.65 to 5.32, *p* = 0.13; *I*^2^ = 22%; four studies) (Figure 6A), treatment success (RR 0.92, 95% CI 0.74–1.14, *p* = 0.46; *I*^2^ = 65%; four studies), and escalation (RR 0.21, 95% CI 0.03–1.70, *p* = 0.14; *I*^2^ = 0%) (Figure 6B). Complications were less frequent after thoracotomy/decortication than after VATS (RR 0.22, 95% CI 0.07–0.71, *p* = 0.011; *I*^2^ = 0%; two studies) but with only two small studies and 9 events between them (Figure 6C).

**Figure 6:**
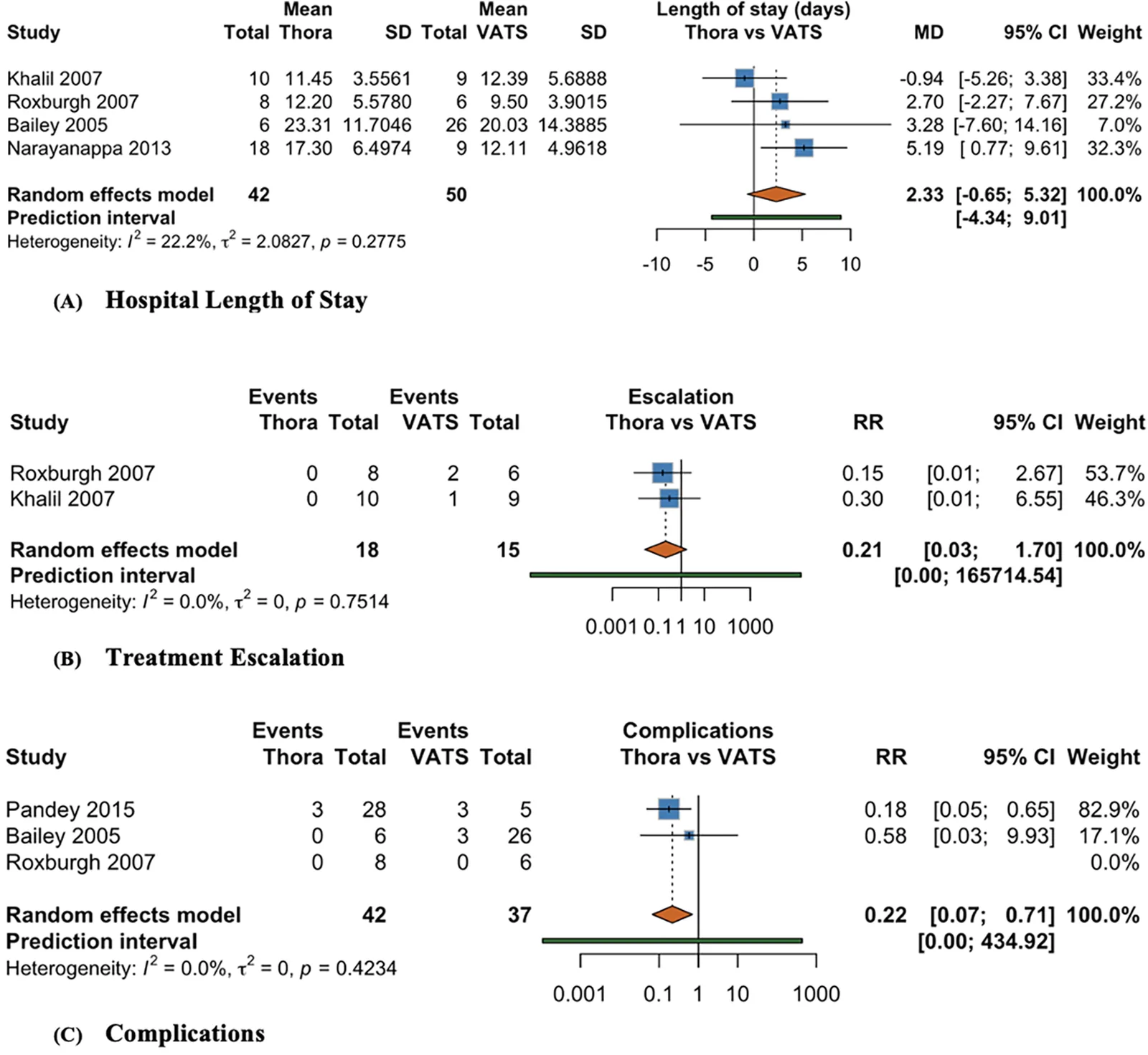
A pairwise meta-analysis comparing thoracotomy/decortication (Thora) and video-assisted thoracoscopic surgery (VATS). **(A)** Hospital length of stay; **(B)** treatment escalation; **(C)** complications.

#### 3.2.6 CT with fibrinolytics versus thoracotomy/decortication

None of the pooled estimates reached significance in this comparison. LOS (MD 2.08 days, 95% CI −5.71 to 9.87, *p* = 0.60; *I*^2^ = 89%), treatment success (RR 0.88, 95% CI 0.71–1.10, *p* = 0.27; *I*^2^ = 93%) (Figure 7A), escalation (RR 6.16 for CT with fibrinolytics vs. thoracotomy, 95% CI 0.49–77.70, *p* = 0.16; *I*^2^ = 86%) (Figure 7B), complications (RR 1.39, 95% CI 0.66–2.93, *p* = 0.39; *I*^2^ = 0%), and mortality (RR 0.34, 95% CI 0.10–1.18, *p* = 0.09; *I*^2^ = 0%) showed no statistically significant difference between groups.

**Figure 7:**
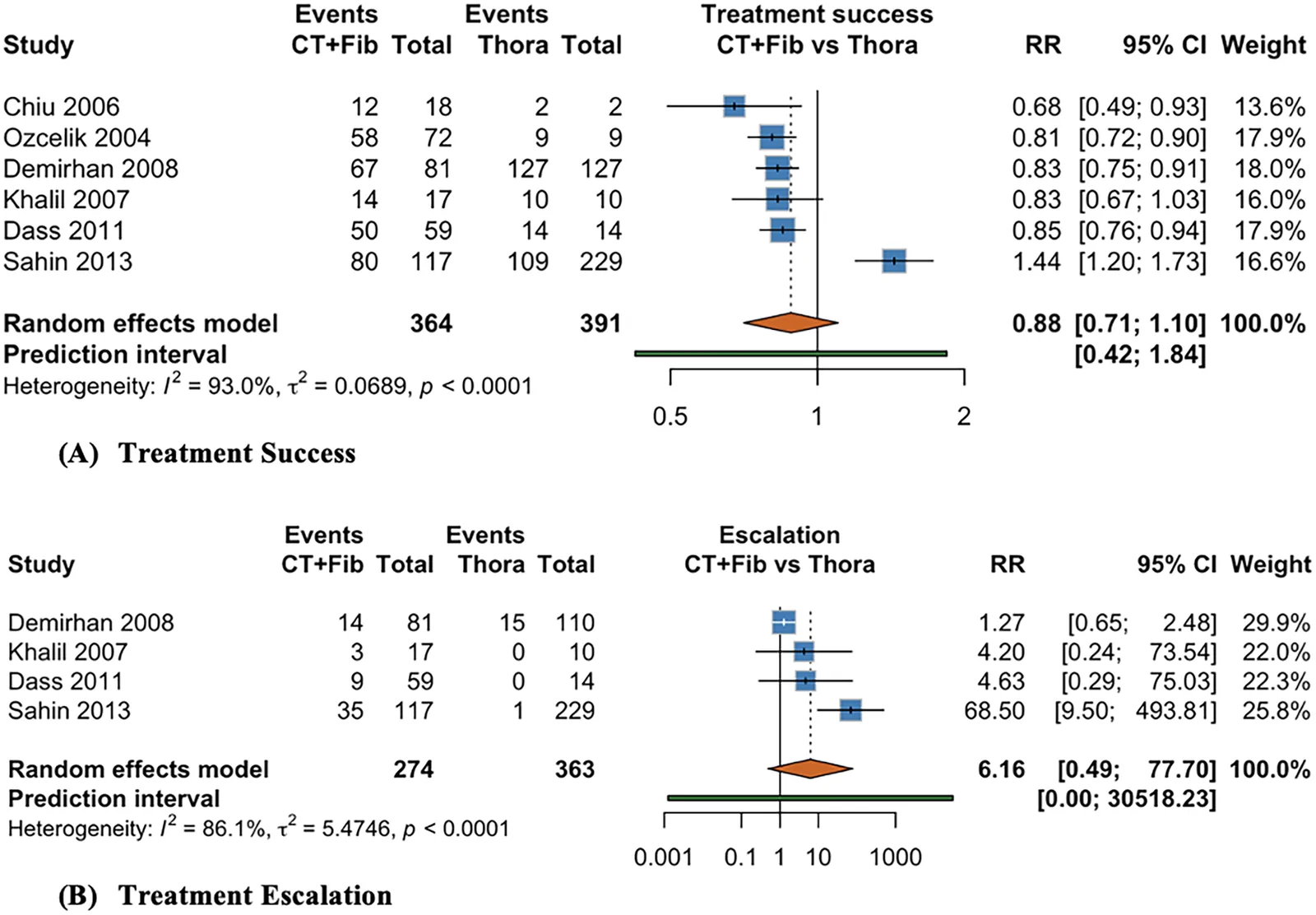
A pairwise meta-analysis comparing chest tube drainage with fibrinolytics (CT + fib) and thoracotomy/decortication (Thora). **(A)** Treatment success; **(B)** treatment escalation.

#### 3.2.7 Outcomes with insufficient data for pooling

Several outcome–comparison cells were informed by only a single study or by studies that reported zero events in both arms and could not be meaningfully pooled. These included drainage and fever duration for the thoracotomy-versus-VATS and fibrinolytics-versus-thoracotomy comparisons, and mortality and complications wherever the surgical arms recorded no events. These cells are reported in the summary tables rather than as forest plots, and no conclusions should be drawn from any single-study or zero-event cell.

Forest plots for all remaining pooled outcome-comparison cells, including those whose pooled estimates did not reach statistical significance, are provided in Supplementary Figures S1–S22.

### 3.3 Network meta-analysis

Shared comparators between the four treatments allowed us to combine the direct and indirect evidence in a single frequentist network meta-analysis for each outcome. The network was restricted to the 40 studies contributing two or more treatment arms, because a contrast-based model requires a within-study comparison; the 37 single-arm studies were retained for the narrative synthesis and the descriptive summaries of treatment utilization but did not enter the network. CT alone was used as the reference treatment throughout, as it is the least invasive option and the natural comparator for the escalating tiers of intervention. All eight outcomes formed a connected network of the four nodes; the geometry, with node sizes proportional to the number of contributing participants and edge widths to the volume of direct evidence, is shown in Figure 8. The networks were closed four-node loops for most outcomes, and therefore, every pairwise contrast was informed by both direct and indirect evidence; the sparser outcomes (mortality, fever) were supported by fewer closed loops. Network geometry was uneven, with greater evidence available for comparisons involving CT, CT with fibrinolytics, and VATS and fewer studies informing thoracotomy comparisons.

**Figure 8:**
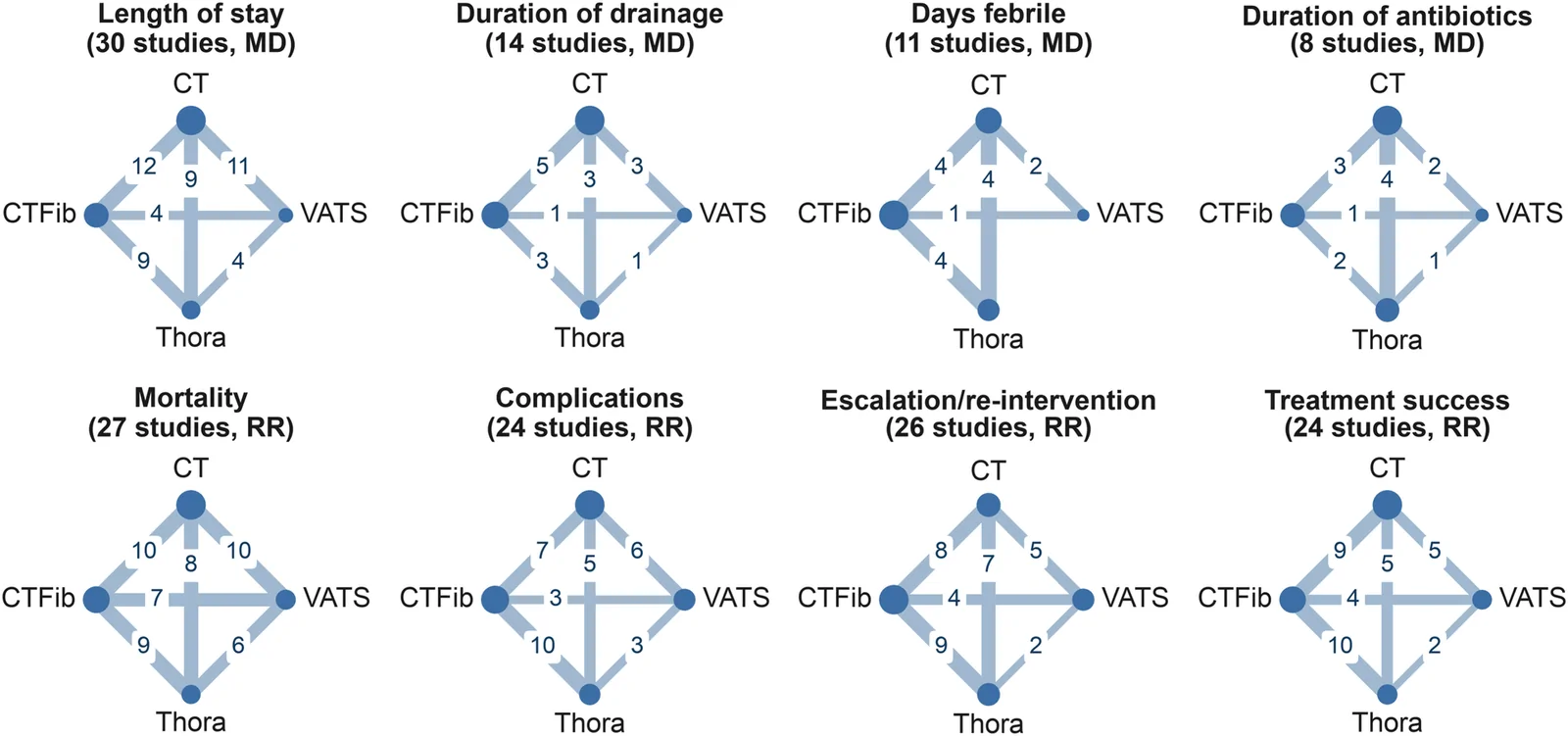
Network geometry for all eight outcomes. Node size is proportional to the number of participants receiving each treatment, and edge width is proportional to the number of studies directly comparing the connected treatments.

#### 3.3.1 Effects relative to CT drainage

Compared with CT alone, the largest relative differences for VATS and thoracotomy were observed for treatment escalation (Figure 9). Treatment escalation was less frequent with VATS, thoracotomy, and fibrinolytics: VATS RR 0.17 (95% CI 0.07–0.37), thoracotomy RR 0.13 (95% CI 0.04–0.36), and CT with fibrinolytics RR 0.48 (95% CI 0.26–0.89). VATS was associated with shorter durations across all three outcomes: drainage was 2.54 days shorter than with a CT alone (95% CI 0.44–4.64), fever resolved 2.00 days sooner (95% CI 0.04–3.97), and antibiotics were stopped 4.95 days earlier (95% CI 0.54–9.36). LOS favored VATS by a similar margin but with a confidence interval that crossed zero (MD 2.06 days, 95% CI −0.45 to 4.57).

**Figure 9:**
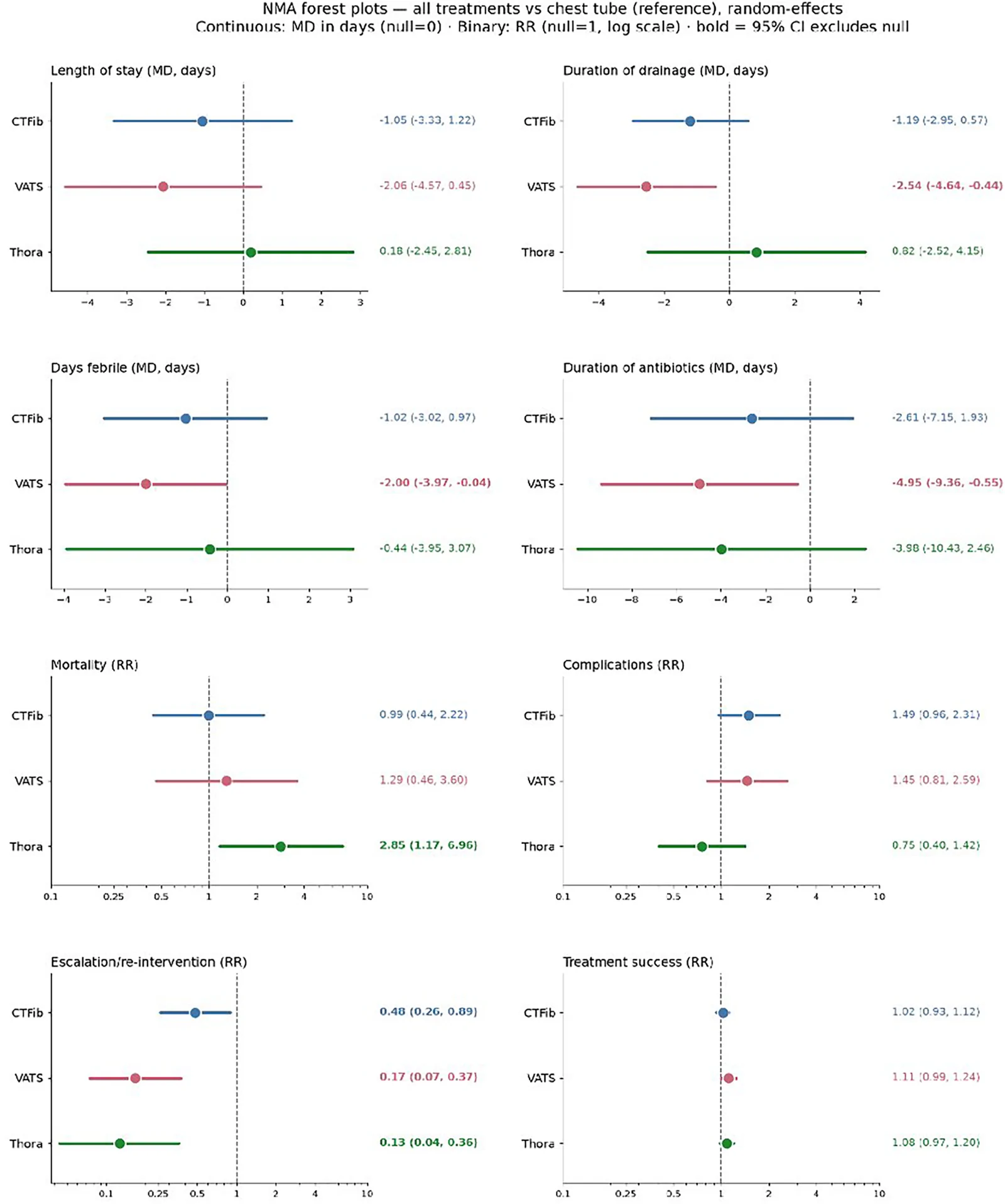
Network meta-analysis estimates for each treatment compared with chest tube drainage (CT) as the reference across all eight outcomes. Effect estimates are presented as mean differences (MDs) for continuous outcomes and risk ratios (RRs) for binary outcomes, with 95% confidence intervals.

No statistically significant difference in treatment success from CT alone was found for any option in the network, although VATS came closest (RR 1.11, 95% CI 0.99–1.24). In the mortality network, thoracotomy was associated with higher mortality than CT (RR 2.85, 95% CI 1.17–6.96). No statistically significant difference in complications was detected for any contrast in the network.

#### 3.3.2 League tables

The full set of network estimates for every pairwise contrast is presented as league tables in Figure 10. In the escalation network, risk was lower relative to CT alone with fibrinolytics (RR 0.48, 95% CI 0.26–0.89), VATS (RR 0.17, 95% CI 0.07–0.37), and thoracotomy (RR 0.13, 95% CI 0.04–0.36), and VATS also significantly outperformed fibrinolytics (RR 0.35, 95% CI 0.17–0.73). No statistically significant difference between the two surgical approaches was detected for escalation (VATS vs. thoracotomy RR 1.32, 95% CI 0.41–4.23), and no contrast reached significance for any other outcome. Because the intervals were wide, these null results do not demonstrate equivalence.

**Figure 10:**
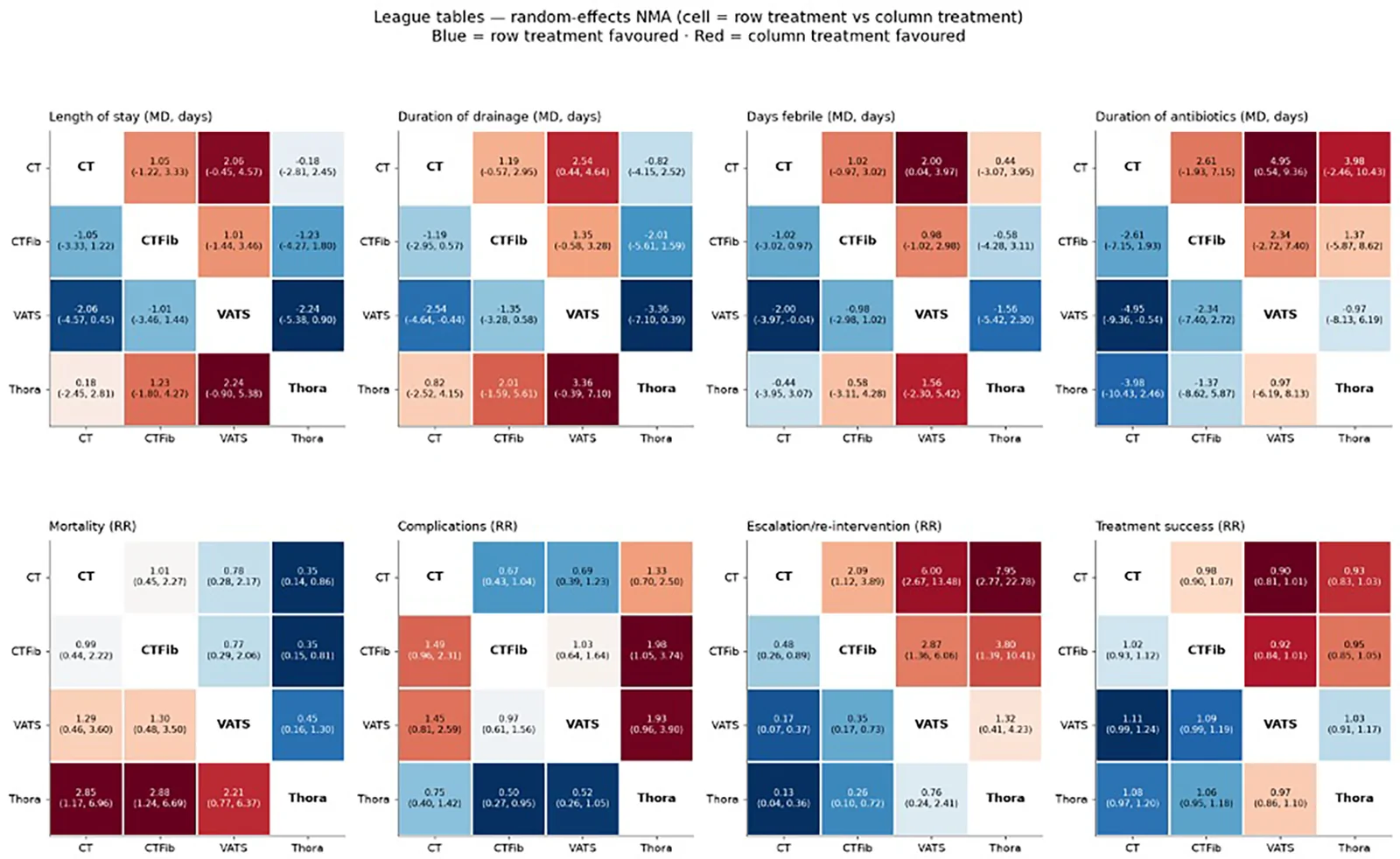
League tables presenting network meta-analysis estimates for all pairwise treatment comparisons across all eight outcomes. Each cell represents the effect of the row treatment relative to the column treatment.

#### 3.3.3 Treatment ranking

Treatments were ranked using P-scores, which summarize the extent of certainty that one treatment performs better than competing treatments based on the network estimates and their standard errors (Figure 11). The rankings depended on the outcome. VATS ranked first for all four duration outcomes and for treatment success (P-scores 0.80–0.96) and second for escalation (0.77) but third for mortality (0.51) and complications (0.23). Thoracotomy ranked first for escalation (0.89) and complications (0.92) but last for LOS (0.25), drainage duration (0.16), and mortality (0.03). CT with fibrinolytics ranked first for mortality (0.73) and last for complications (0.17). CT alone ranked last for fever, antibiotic duration, escalation, and treatment success (0.00–0.19) but second for mortality (0.72) and complications (0.68). Rankograms showed more concentrated ranking probabilities for outcomes with greater available evidence and more widely distributed probabilities for mortality and complications (Figure 11). As an exploratory summary only, the unweighted mean of the eight outcome-specific P-scores was 0.74 for VATS, 0.50 for thoracotomy, 0.47 for CT with fibrinolytics, and 0.29 for CT alone. No weighting was prespecified, and the outcomes differ in clinical importance, evidence base, and susceptibility to confounding. This mean describes the pattern of rankings only and is not a measure of overall effectiveness.

**Figure 11:**
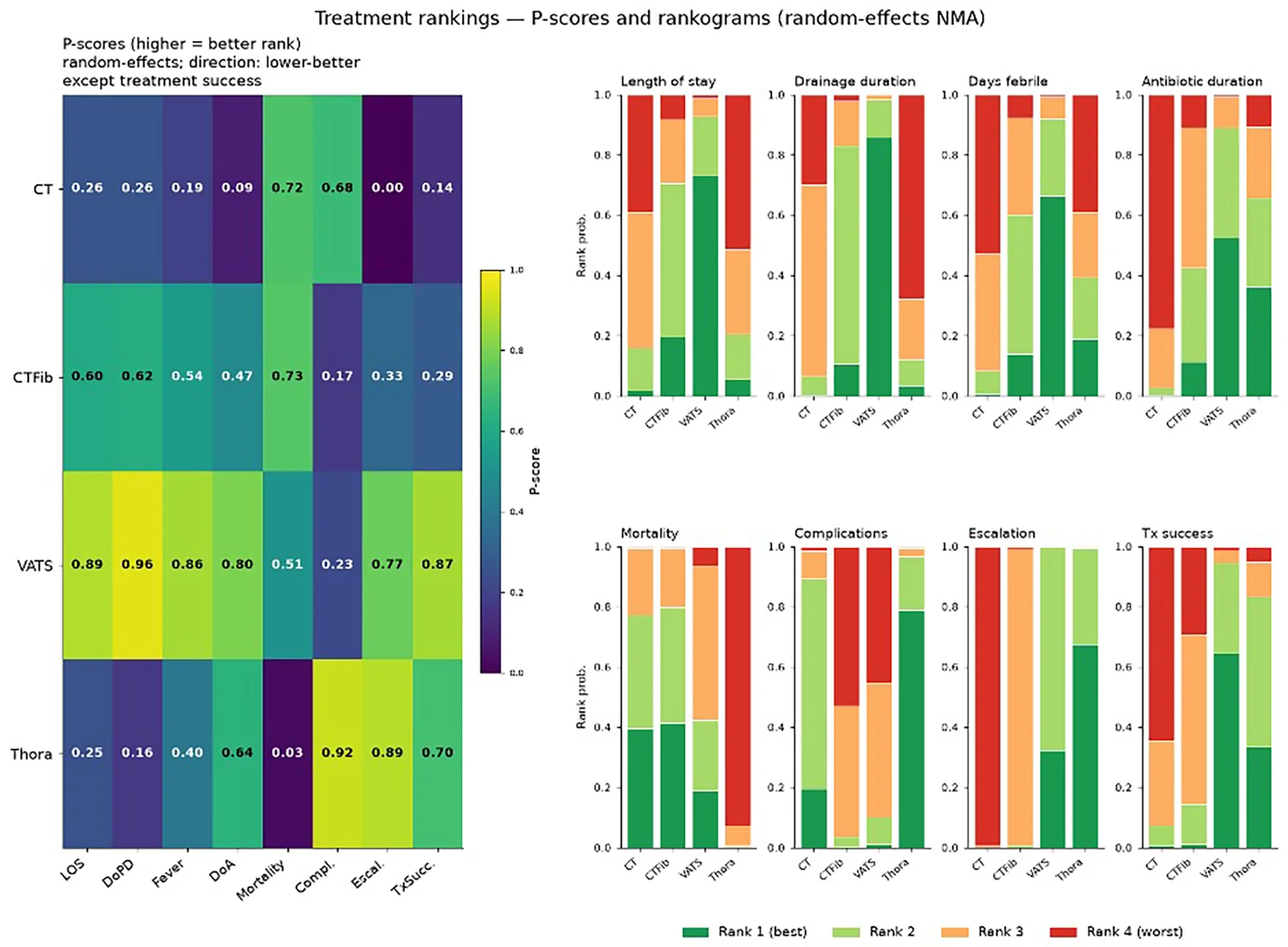
Treatment rankings based on P-scores across all eight outcomes, with the corresponding rankograms showing the probability of each treatment occupying each rank. Higher P-scores indicate more favorable relative rankings.

### 3.4 Assessment of inconsistency

Agreement between direct and indirect evidence was assessed both locally and globally. Locally, node-splitting compared direct and indirect estimates for each treatment comparison informed by both sources of evidence (Supplementary Figure S23). Node-splitting identified one comparison with significant disagreement between direct and indirect evidence: for treatment success, the direct estimate for CT with fibrinolytics versus CT alone (RR 0.958) differed from the indirect estimate (RR 1.310; *p* = 0.006). All other comparisons showed no significant difference between direct and indirect estimates. For treatment escalation, the random-effects global test gave *p* = 0.86, indicating no evidence of inconsistency across the network.

Under the fixed-effect model, the global design-by-treatment interaction test indicated significant inconsistency in five of the eight networks. Under the random-effects model, significant inconsistency remained in three networks: drainage duration (*p* = 0.017), fever duration (*p* < 0.001), and treatment success (*p* = 0.023). No significant global inconsistency was detected for LOS or antibiotic duration. Net-heat plots (Supplementary Figure S24) showed no localized areas of inconsistency within the treatment escalation network. The random-effects test is the prespecified assessment (Section 2.7), and its results are reported as the primary finding. Where fixed- and random-effects results differed, interpretation was based on the prespecified random-effects model because the fixed-effect model does not account for between-study heterogeneity. Inconsistency was therefore confined to the drainage duration, fever duration, and treatment success networks, and the network estimates for these three outcomes should be interpreted with corresponding caution and regarded as less certain than the corresponding direct estimates (Section 3.9).

### 3.5 Heterogeneity

Heterogeneity varied sharply by outcome type (Supplementary Figure S25). Among binary outcomes, escalation showed moderate network heterogeneity (*I*^2^ = 54%), and mortality and complications were low. As for the continuous duration outcomes, network *I*^2^ values for LOS, drainage, and fever ranged between 80% and 90%.

Several clinical and methodological differences may have contributed to this heterogeneity. For the duration outcomes, discharge criteria, thresholds for drain removal, antibiotic-duration policies, and the timing of referral for surgery differ between institutions and countries. These factors were rarely reported but may directly influence the measured durations and varied across studies. Treatment-line composition also differed across studies (Section 3.1). Where a surgical arm was a step-up procedure, the reported durations may or may not include the period spent under the failed initial strategy. Source reports rarely stated this clearly. Case mix by empyema stage could not be examined because stage was reported in a minority of studies, and the fibrinolytic node combines agents, doses, and schedules. These sources are considered further in Section 4.1.

### 3.6 Sensitivity analysis

Both prespecified sensitivity analyses were applied to all 39 pooled outcome-by-comparison estimates. In the leave-one-out analysis, the direction of effect was unchanged in 32 of the 39 estimates; the seven exceptions were all comparisons whose confidence interval already included the null. Of the 11 estimates that were statistically significant in the primary analysis, three retained significance across every iteration: drainage duration for CT with fibrinolytics versus VATS, and treatment escalation for both CT alone versus VATS and CT with fibrinolytics versus VATS, while the remaining eight lost significance when at least one study was omitted. Refitting every estimate with restricted maximum likelihood and Hartung–Knapp confidence intervals reproduced the direction of effect in all 39 analyses and statistical significance in 34; the five divergences all arose in comparisons of two to four studies, where the Hartung–Knapp interval is appreciably wider. The three findings that withstood both checks are the only pooled results that were robust to both the exclusion of individual studies and the choice of variance estimator. However, robustness in sensitivity analyses does not imply high certainty of evidence. All three come largely from non-randomized comparisons and were rated moderate certainty after downgrading for risk of bias (Supplementary Table S7). Accordingly, these findings remained limited by the moderate certainty of the underlying evidence.

The primary-line sensitivity analysis (Section 2.5) retained 22 of the 30 studies in the LOS network, nine of 14 for drainage duration, nine of 11 for fever, and seven of eight for antibiotic duration. It also retained 17 of 27 studies for mortality, 16 of 24 for complications, 21 of 26 for escalation, and 17 of 24 for treatment success (Supplementary Table S8; Supplementary Figure S27). Thoracotomy left the drainage-duration and fever networks entirely, because every thoracotomy arm reporting those outcomes was a step-up or rescue procedure. For antibiotic duration, escalation, and treatment success, it was informed by only one to three primary-line studies. The six thoracotomy contrasts for drainage and fever are therefore not estimable, and its remaining estimates are sparse. Of the 42 estimable contrasts, the direction of effect was unchanged in 32 and statistical significance in 31. The escalation ordering was preserved. Relative to CT alone, RR was 0.49 (95% CI 0.25–0.97) for CT with fibrinolytics, 0.15 (0.07–0.35) for VATS, and 0.02 (0.003–0.12) for thoracotomy, the last from three studies. The VATS duration advantages persisted, with drainage MD −3.74 days (95% CI −5.49 to −1.98), fever MD −2.25 (95% CI −4.47 to −0.02), and antibiotics MD −4.97 (95% CI −9.56 to −0.38). LOS did not reach significance in the principal network, but it did so for VATS (MD −3.57, 95% CI −6.02 to −1.13) and for CT with fibrinolytics (−2.61, −4.92 to −0.31). Treatment success also became significantly higher than with CT alone for CT with fibrinolytics (RR 1.24, 95% CI 1.07–1.44) and VATS (1.34, 1.13–1.58). The changes were concentrated in the thoracotomy contrasts. The higher network mortality with thoracotomy relative to CT no longer reached significance (RR 2.32, 95% CI 0.54–10.0, versus 2.85 in the principal network). The apparent complication advantage of thoracotomy over CT with fibrinolytics reversed the direction (RR 1.27, 95% CI 0.36–4.47, versus 0.50). Thoracotomy also moved from the least to the most favorable P-score for LOS (MD −4.83 days versus CT, 95% CI −8.99 to −0.66). Exploratory mean P-scores over the estimable outcomes were 0.80 for VATS, 0.52 for thoracotomy, 0.50 for CT with fibrinolytics, and 0.19 for CT alone. The thoracotomy mean rests on six outcomes, three of them sparse, and is not comparable with the others.

### 3.7 Quality assessment

Of the 77 included studies, 69 were non-randomized and were therefore appraised with the ROBINS-I tool, of which 19 were judged at critical and 49 at serious overall risk of bias, and one at moderate risk (Supplementary Table S5A). Randomized trials (*n* = 5) were appraised using RoB 2; one was at low risk of bias and four raised some concerns (Supplementary Table S5B). Four studies were single-arm case series and were therefore appraised with the JBI critical appraisal checklist for case series. Kercher 2000 reported as a case series also contributed a between-strategy comparison to the pooled analyses and was therefore appraised with both ROBINS-I and the JBI checklist. The distribution of risk-of-bias judgments across the individual pooled analyses, together with the resulting certainty downgrades, is given in Supplementary Table S6.

Confounding determined the overall judgment in every non-randomized study: no study reported an effect estimate adjusted for stage of empyema at presentation, a major determinant of both treatment selection and clinical outcomes. Deviations from intended interventions were at serious risk in 40 studies, reflecting step-up protocols in which children crossed over to surgery after failed drainage, a judgment made against the initiation-of-treatment estimand defined in Section 2.6; selection of participants was at serious risk in 16. Reporting domains performed better: missing data were at low or moderate risk in 68 studies, and no study was at serious risk for measurement of outcomes or for selection of the reported result. Consequently, all 47 outcome-by-comparison cells appraised for risk of bias were downgraded on that domain, 28 by two levels and 19 by one, and none was free of serious concerns; 39 of these 47 yielded an estimable pooled effect and were carried forward to a full certainty rating (3.9). Randomized evidence covers only one of the six comparisons: all four contributing trials address CT with fibrinolytics versus VATS, with up to four randomized studies in a single cell. All 28 two-level downgrades occurred in analyses containing no randomized studies.

### 3.8 Small-study effects and publication bias

Small-study effects were examined with comparison-adjusted funnel plots for the networks with sufficient studies (k ≥ 10) and with Egger's regression test at the pairwise level (Supplementary Figure S26). The funnels for the well-populated networks, most importantly escalation, were broadly symmetric, and the Egger test for escalation showed no asymmetry (*p* = 0.88). However, asymmetry was observed for some comparisons. The treatment-success contrast between CT with fibrinolytics and CT alone showed evidence of asymmetry (Egger *p* = 0.003), as did the fever-duration network (*p* = 0.002), raising the possibility of small-study effects for these outcomes. Given the small number of contributing studies, these findings should be interpreted cautiously and do not establish the presence or absence of publication bias.

### 3.9 Certainty of evidence

Certainty of evidence was rated for each of the 39 pooled outcome-by-comparison analyses (Figure 12; Supplementary Table S7). No comparison reached high certainty: eight were rated moderate, four low, and 27 very low. Certainty was highest for treatment escalation, where three of the six comparisons reached moderate certainty, and was uniformly very low for hospital length of stay, where all six comparisons were downgraded for serious risk of bias combined with substantial heterogeneity and imprecision. Downgrading was driven principally by risk of bias, which was serious or critical in every cell, and by imprecision arising from the small number of studies contributing to most comparisons. A further eight outcome-by-comparison cells were appraised for risk of bias but could not be pooled. Five were informed by a single study, and three were mortality comparisons in which no deaths, or a single death overall, were recorded, leaving the RR inestimable. Certainty was therefore not rated for these cells (Supplementary Table S6). These ratings apply to the direct pairwise estimates. Network estimates also depend on transitivity, which could not be verified for stage, severity, or treatment line (Section 2.7; Supplementary Table S9), and on coherence, which was violated for drainage duration, fever duration, and treatment success (Section 3.4). Network estimates for those three outcomes should be regarded as less certain than the corresponding direct estimates, and no network estimate should be regarded as more certain than the direct evidence that informs it.

**Figure 12:**
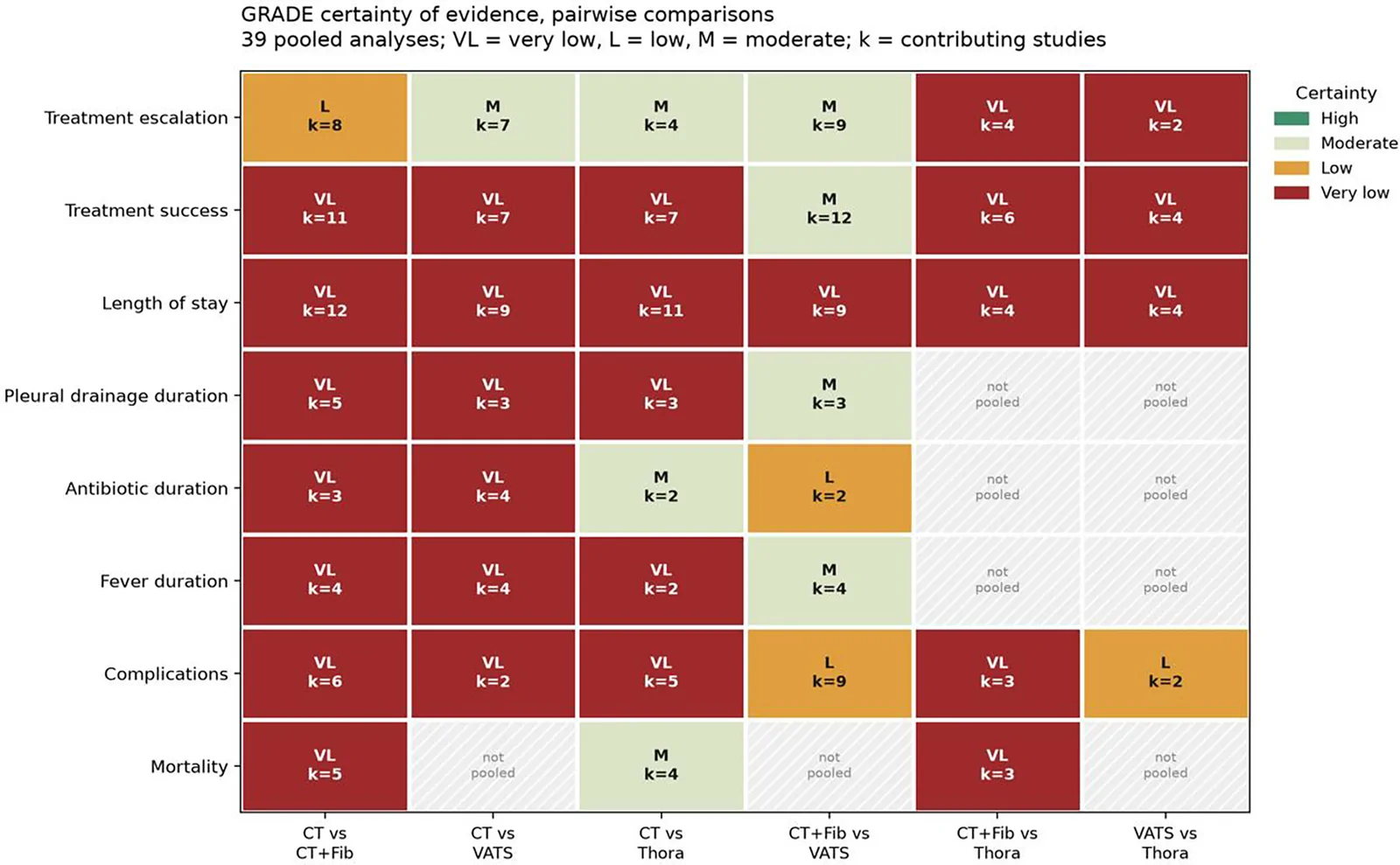
GRADE certainty of evidence for all 39 pooled outcome-by-comparison analyses. The cells are shaded by certainty (moderate, low, very low; no comparison reached high certainty) and annotated with the number of contributing studies (k). Hatched cells could not be pooled, either because no study or only one study contributed, or because no events or only one event overall were observed. CT, chest tube drainage; CT + Fib, chest tube drainage with intrapleural fibrinolytics; VATS, video-assisted thoracoscopic surgery; Thora, thoracotomy with decortication.

## 4 Discussion

This review compared four treatment strategies for pediatric empyema thoracis across eight outcomes in 77 studies and 7,378 children, although the effective sample for any single comparison was far smaller. Individual pooled estimates were informed by two to 12 studies. Four principal findings emerged. First, treatment escalation was less frequent after VATS, thoracotomy or CT with fibrinolytics than after CT alone, and less frequent after VATS than after CT with fibrinolytics. This is the finding with the strongest support. Three of the six escalation comparisons reached moderate certainty, and the ordering held in direction and significance when the network was restricted to primary-line arms. Second, VATS was associated with shorter pleural drainage, fever, and antibiotic duration than CT alone, at low to very low certainty because of a serious risk of bias, heterogeneity, and imprecision. These associations also persisted in the primary-line analysis. Third, the higher network mortality with thoracotomy, and its apparently lower complication rate relative to CT with fibrinolytics, did not persist when step-up and rescue arms were excluded. This attenuation is consistent with confounding by indication related to treatment line. Fourth, no pooled estimate reached high certainty. Eight were moderate, four low, and 27 very low, and because transitivity could not be verified, no network estimate is more certain than the direct evidence informing it. Treatment success rates exceeded 85%–90% with every strategy, limiting the ability of this outcome to discriminate between treatments. Randomized evidence exists for only one comparison, drawn from four trials, all of CT with fibrinolytics against VATS. Previous trial reports have found similar treatment success and complication rates between these modalities; interpretation should therefore prioritize randomized evidence (90, 100). The remaining five treatment comparisons were supported exclusively by observational evidence. P-score rankings summarize these outcome-specific associations and are reported as exploratory rather than constituting a treatment hierarchy. CT alone was associated with more escalation than any alternative in every analysis, and VATS and CT with fibrinolytics differed mainly in escalation and drainage duration and not in success or safety. These associations come largely from non-randomized comparisons and are hypotheses for trials and not grounds for a recommendation.

By requiring microbiological, radiological, biochemical, or procedural/surgical confirmation of empyema, the eligibility criteria improved the specificity of the case definition relative to reviews that accepted any parapneumonic effusion. These criteria improved specificity but did not ensure comparability in baseline disease severity, particularly because stage was reported in only one-third of studies and severity was rarely quantified. In contrast to recent meta-analyses, which either focused on head-to-head comparisons of two treatment modalities or evaluated a limited range of outcomes, the present review compared all major treatment strategies using both pairwise and network meta-analyses (4, 97, 98).

VATS was associated with lower escalation than CT alone and CT with fibrinolytics, and with shorter drainage, fever, and antibiotic durations than CT alone. Although VATS ranked favorably across several outcomes, P-scores do not incorporate effect magnitude or certainty. Gates et al. were among the first to compare the four treatment modalities, reporting a shorter LOS particularly among children undergoing early VATS or thoracotomy (99). However, with advancements of further therapies, including the increased use of fibrinolytics, subsequent comparative studies have provided inconclusive evidence regarding the optimal treatment modality, particularly when comparing VATS with CT with fibrinolytics (89, 97, 98, 100). Fernandez Elviro et al., for instance, found no significant difference between VATS and CT with fibrinolytics; however, their analysis primarily evaluated LOS as the main outcome measure (97). In contrast, our analysis incorporated multiple clinically meaningful outcomes and found that VATS was associated with a significantly lower risk of treatment escalation compared with both CT alone and CT with fibrinolytics. In addition, VATS was associated with shorter pleural drainage duration and reduced postintervention fever duration. No statistically significant increase in complications or mortality was detected with VATS, although the intervals do not exclude clinically relevant differences. Pacilli et al. similarly demonstrated favorable outcomes with VATS compared with fibrinolytic therapy, including fewer reinterventions and a slightly shorter LOS, while reporting comparable complication rates between treatment approaches (98). Previous RCTs directly comparing VATS with CT with fibrinolytics have also demonstrated similar treatment success and complication rates between modalities, primarily aiming to establish that VATS does not result in prolonged hospitalization compared with fibrinolytic therapy (90, 100). However, these studies were limited by relatively small sample sizes and were often underpowered to detect differences in treatment failure or escalation.

The 2026 IDSA/PIDS guideline recommends CT with fibrinolytics as first-line therapy for most children requiring pleural drainage (8). This recommendation is based on comparable overall outcomes between fibrinolytics and VATS, while recognizing the less invasive nature, lower cost, and reduced requirement for general anesthesia associated with fibrinolytic therapy (60, 89, 100, 101). The guideline generally reserves VATS for selected patients with extensive loculations, advanced disease, or failure of initial management with chest tube drainage and fibrinolysis (8). Previous studies, including Mahant et al., found insufficient evidence to demonstrate the superiority of VATS over fibrinolytics because of limited randomized evidence and small sample sizes (102). Conversely, more recent systematic reviews, such as Miscia et al., have reported advantages associated with VATS, including shorter LOS, reduced drainage duration, and faster resolution of postoperative fever (4). In our analysis, CT with fibrinolytics was associated with longer drainage duration and approximately a 3-fold higher risk of escalation than VATS (moderate certainty for escalation). No statistically significant difference was detected for LOS, antibiotic duration, or complications. This does not establish that fibrinolytics are inferior. The guideline considers the lower invasiveness and avoidance of general anesthesia alongside the possibility of subsequent escalation. Our findings therefore quantify the difference in treatment escalation without contradicting the broader rationale underlying current treatment recommendations.

Consistent with previous evidence, CT alone was associated with more escalation than every alternative in the pairwise, network, and primary-line analyses, and with longer drainage and antibiotic duration in the network. Prior studies have reported higher treatment failure rates, longer LOS, longer drainage duration, and a greater likelihood of escalation compared with other modalities (2, 97, 99, 103). In our analysis, CT alone ranked last for four of the eight outcomes (fever, antibiotic duration, escalation, and treatment success) but second for mortality and complications. Because CT alone was almost always a primary strategy, its escalation estimates are also the least affected by the treatment-line imbalance discussed below. These findings are consistent with reduced use of CT drainage alone as definitive therapy where additional modalities are available, but their observational basis precludes a causal treatment recommendation.

The principal analyses detected no statistically significant difference between thoracotomy and VATS for treatment success or escalation. The intervals were wide, and the comparison rests on few studies. Equivalence is therefore not established. These findings align with previous comparative evidence demonstrating similar clinical efficacy between surgical approaches. The recent network meta-analysis by Fernandez Elviro et al. similarly reported that both VATS and thoracotomy reduced hospital LOS compared with CT alone, although direct comparisons between the surgical modalities remained limited (97). The higher mortality observed with thoracotomy should be interpreted cautiously. This estimate was based on very few events and is particularly susceptible to confounding by indication, as thoracotomy was generally reserved for patients with more severe or refractory disease, consistent with the 2017 American Association for Thoracic Surgery consensus guidelines for the management of empyema (104). Therefore, this finding may reflect differences in baseline disease severity and treatment selection. This interpretation was supported by the primary-line sensitivity analysis. When step-up and rescue arms were excluded, the thoracotomy mortality estimate no longer reached significance (RR 2.32, 95% CI 0.54–10.0), and its apparent complication advantage over CT with fibrinolytics reversed the direction. The thoracotomy node also lost all of its drainage-duration and fever evidence and became sparse for the remaining outcomes (Section 3.6). Similar concerns regarding selection bias and confounding by disease severity have been highlighted in observational comparisons of surgical approaches, limiting causal interpretation of mortality differences (5, 105). Similarly, the significant pairwise difference in complications between VATS and thoracotomy was based on only two small studies and nine events and should therefore be considered an unstable estimate rather than evidence of a meaningful difference in safety between the two surgical approaches.

Treatment escalation was the outcome with the most consistent evidence in our analysis. Its direction was consistent across pairwise, network, and all three sensitivity analyses, and the network showed no significant inconsistency between direct and indirect evidence. Its interpretation nevertheless depends on treatment line. We counted escalation from the assigned intervention onward, and because fewer more-invasive options remained after a step-up surgical procedure, this definition may bias escalation comparisons in favor of surgical arms. This makes escalation estimates involving thoracotomy particularly difficult to interpret and means that this outcome on its own may not be used as a valid basis for ranking treatment strategies. The finding chiefly rests on the CT-versus-VATS and fibrinolytics-versus-VATS comparisons, in which 73%–100% of arms on both sides were primary line (Supplementary Table S9). This contrasts with the greater heterogeneity and inconsistency observed for several duration-based outcomes. Treatment success rates were high across all four modalities, generally exceeding 85%–90%, which limited the ability of the outcome to discriminate between treatment strategies. This ceiling effect, compounded by variation in how success was defined, may explain why treatment success was a less statistically informative outcome. Previous meta-analyses, including Fernandez Elviro et al., have primarily focused on LOS, while evidence regarding escalation has been limited by inconsistent reporting and reliance mainly on RCTs (97). Similarly, previous systematic reviews that evaluated treatment failure have been unable to establish definitive conclusions because of small sample sizes and limited statistical power (102). Including observational studies increased the evidence for treatment escalation but also introduced greater susceptibility to confounding. Although previous network meta-analyses have characterized CT drainage alone primarily through duration outcomes, our findings indicate that escalation is a clinically relevant endpoint that should be defined and reported consistently in future comparative studies (99, 103).

### 4.1 Strengths and limitations

The strengths of this review include the use of prespecified diagnostic criteria for Stage II/III empyema thoracis, incorporating biochemical, radiological, microbiological, procedural, and surgical criteria, thereby improving the specificity of the included populations. Our analysis drew on 77 studies and 7,378 children, although this aggregate overstates the evidence for any single comparison. Individual pooled estimates were informed by two to 12 studies. The thoracotomy comparisons in particular rested on a few studies, and after restriction to primary-line arms, on one to three or on none. Moreover, we compared multiple treatment modalities, including CT alone, CT with fibrinolytics, VATS, and thoracotomy/decortication, using both pairwise and network meta-analysis. Finally, our study evaluated multiple clinically relevant outcomes beyond hospital LOS, including treatment escalation, treatment success, duration of pleural drainage, antibiotic duration, postintervention fever, complications, and mortality. This broader assessment allowed for a more thorough evaluation of treatment effectiveness and safety.

Although the inclusion of multiple study designs allowed us to incorporate a broad range of clinical evidence, only five of the 77 included studies were RCTs, with the remainder being observational. This limits causal inference and introduces substantial potential for confounding by indication, as children with more severe or advanced disease may have been preferentially selected for more invasive treatment. None of the included observational comparisons adjusted for empyema stage at presentation, potentially biasing estimates against surgical strategies by attributing outcomes related to greater baseline disease severity to the treatment itself. This concern is particularly relevant in cohorts using step-up protocols, where children underwent surgery only after failure of initial drainage, inherently selecting a more treatment-refractory population for the surgical group. The primary-line sensitivity analysis quantifies this. Restricting the network to primary arms removed thoracotomy from two networks, left it sparsely informed in the others, and reversed or attenuated its mortality and complication estimates. The escalation and duration associations for VATS and CT with fibrinolytics were preserved (Section 3.6). That analysis has limitations of its own. Treatment-line classification relied on the descriptions in source reports and could not be resolved for five study-by-treatment units. Four additional units combined primary and step-up patients within one treatment node and were excluded from the restricted network, and the restriction was added during peer review rather than prespecified. Comparative estimates from observational studies should therefore be interpreted cautiously and should not be assumed to represent the causal effect of selecting one treatment strategy over another. Substantial heterogeneity was also present for several duration outcomes, likely reflecting differences in clinical practice, including referral timing, discharge criteria, and thresholds for CT removal or treatment escalation. Inconsistency in the drainage duration and fever duration networks further warrants caution when interpreting these estimates. Treatment success definitions varied across studies and thus showed network inconsistency, limiting comparability for this outcome. The fibrinolytics node combined different agents, doses, and administration schedules and was therefore clinically heterogeneous. Agent-, dose-, and schedule-specific effects could not be estimated, and this within-node heterogeneity may have contributed to inconsistency in the duration outcomes. Empyema stage was reported in only a minority of studies, preventing meaningful stage-specific analyses.

Transitivity could not be verified because important effect modifiers, particularly disease stage and severity, were inconsistently reported and differed across direct comparisons, limiting our ability to confirm their comparable distribution across the network. The reported effect modifiers also differed across direct comparisons. The six direct comparisons differed in the proportion of arms delivered as primary treatment (21%–38% of thoracotomy arms versus 60% or more in eight of the nine other node-by-comparison cells). They also differed in the proportion of studies reporting stage (14%–40%), and in the presence of randomized evidence, which was confined to the fibrinolytics-versus-VATS comparison (Supplementary Table S9). Network connectivity alone does not establish exchangeability across comparisons, and the network estimates should be interpreted with this limitation in mind. Although consistency was supported for several networks, evidence of inconsistency for some outcomes indicates that direct and indirect evidence was not universally concordant. Network geometry was also uneven, with fewer studies informing some comparisons, particularly those involving thoracotomy; consequently, several network estimates were informed by relatively sparse direct evidence and were less precise. At the review level, restriction to English-language publications may have resulted in the omission of relevant evidence, and reporting or publication bias cannot be excluded, particularly for comparisons supported by few studies. Formal assessment of publication bias was also limited by the small number of studies in some comparisons, although asymmetry was detected for treatment success comparing CT with fibrinolytics and CT alone (Egger *p* = 0.003). These limitations have similarly been highlighted in previous systematic reviews, where comparisons between fibrinolytics and VATS have been constrained by heterogeneous trial designs, small sample sizes, and lack of standardized outcome reporting (96, 97, 100, 103).

### 4.2 Future directions

With moderate certainty, CT alone was associated with a greater need for subsequent intervention than other strategies. Whether earlier definitive intervention would improve outcomes for a given child is a hypothesis that these data generate rather than one they test. The predominantly observational evidence base, the unverified transitivity assumption, and the treatment-line imbalance do not support a treatment hierarchy or a recommendation favoring one strategy for all patients. Our findings do not contradict current guideline recommendations for CT with fibrinolytics as first-line therapy. Rather, they estimate the difference in subsequent treatment escalation between treatment strategies. These findings support individualized treatment selection according to disease severity, local expertise, resource availability, and response to initial therapy, while promoting standardized definitions of treatment success and escalation to improve consistency across institutions.

Future research should prioritize well-designed prospective studies and RCTs using explicit diagnostic criteria for pediatric empyema thoracis. Although several RCTs comparing treatment strategies have previously been conducted and incorporated into meta-analyses, many applied broad or poorly defined eligibility criteria that did not reliably distinguish Stage II or III empyema from uncomplicated or early parapneumonic effusions. Consequently, several of these trials did not meet the diagnostic criteria required for inclusion in the present review, limiting the available high-quality randomized evidence specific to established empyema. Clinical trials should therefore use explicit biochemical, microbiological, radiological, procedural, or operative criteria to confirm empyema and should systematically report disease stage and baseline severity. Standardized definitions of treatment success, treatment failure, escalation, and discharge criteria are also needed to improve comparability across studies. Adequately powered RCTs directly comparing VATS with CT with fibrinolytics using these criteria would help determine whether the differences observed in treatment escalation and other outcomes represent true treatment effects. Greater standardization of fibrinolytic regimens and procedural techniques, alongside assessment of long-term, patient-centered, and cost-effectiveness outcomes, would further strengthen the evidence base and support more definitive treatment recommendations.

### 4.3 Conclusion

This systematic review and meta-analysis compared the four principal treatment strategies for pediatric empyema thoracis across eight outcomes. The most consistent finding was that treatment escalation occurred less often after VATS, thoracotomy, or CT with fibrinolytics than after CT alone. Three of six escalation comparisons were supported by moderate-certainty evidence. Escalation was also less frequent after VATS than after CT with fibrinolytics. This ordering did not change when the analysis was restricted to primary-line treatment. VATS was also associated with shorter drainage, fever, and antibiotic duration than CT alone, at low to very low certainty. No statistically significant increase in complications or mortality was detected. Estimates involving thoracotomy, including its higher network mortality, were strongly influenced by the frequent use of thoracotomy as rescue treatment in refractory disease. Because the evidence is predominantly observational, the network's transitivity assumption could not be verified, and no estimate reached high certainty. As a result, these associations do not establish a treatment hierarchy. They instead identify priorities for adequately powered trials with explicit disease staging and standardized outcome definitions, particularly VATS versus CT with fibrinolytics for treatment escalation. Until then, treatment selection should continue to rest on disease stage and severity, clinical response, and local expertise.

## Data Availability

The original contributions presented in the study are included in the article/Supplementary Material, and further inquiries can be directed to the corresponding author.
