# Supplementary material for: Comparative effectiveness of treatment strategies for pediatric empyema thoracis: a systematic review with pairwise and network meta-analyses
Source: Front Pediatr. 2026 Oct 26;14:1980275. doi: 10.3389/fped.2026.1980275 (PMC13745557; doi:10.3389/fped.2026.1980275)
Supplement: Supplementary file 2 [file Supplementaryfile1.docx]

Supplementary Material

# 1 Supplementary Figures

**Supplementary Figure S1.** Complications — CT+Fib versus VATS. Random-effects meta-analysis of the risk ratio (RR) with 95% confidence intervals. Heterogeneity: I² = 0.0%, τ² = 0, p = 0.5368.

**Supplementary Figure S2.** Duration of antibiotics (days) — CT+Fib versus VATS. Random-effects meta-analysis of the mean difference (MD) with 95% confidence intervals. Heterogeneity: I² = 0.0%, τ² = 0, p = 0.3596.

**Supplementary Figure S3.** Length of stay (days) — CT+Fib versus VATS. Random-effects meta-analysis of the mean difference (MD) with 95% confidence intervals. Heterogeneity: I² = 84.0%, τ² = 3.9818, p < 0.0001.
**Supplementary Figure S4.** Complications — CT+Fib versus CT alone. Random-effects meta-analysis of the risk ratio (RR) with 95% confidence intervals. Heterogeneity: I² = 74.1%, τ² = 0.7236, p = 0.0017.

**Supplementary Figure S5.** Duration of antibiotics (days) — CT+Fib versus CT alone. Random-effects meta-analysis of the mean difference (MD) with 95% confidence intervals. Heterogeneity: I² = 84.4%, τ² = 14.6780, p = 0.0016.

**Supplementary Figure S6.** Duration of drainage (days) — CT+Fib versus CT alone. Random-effects meta-analysis of the mean difference (MD) with 95% confidence intervals. Heterogeneity: I² = 83.4%, τ² = 6.2570, p < 0.0001.

**Supplementary Figure S7.** Fever duration (days) — CT+Fib versus CT alone. Random-effects meta-analysis of the mean difference (MD) with 95% confidence intervals. Heterogeneity: I² = 91.4%, τ² = 15.4218, p < 0.0001.

**Supplementary Figure S8.** Length of stay (days) — CT+Fib versus CT alone. Random-effects meta-analysis of the mean difference (MD) with 95% confidence intervals. Heterogeneity: I² = 87.6%, τ² = 23.5055, p < 0.0001.

**Supplementary Figure S9.** Mortality — CT+Fib versus CT alone. Random-effects meta-analysis of the risk ratio (RR) with 95% confidence intervals. Heterogeneity: I² = 28.6%, τ² = 0.6645, p = 0.2308

**Supplementary Figure S10.** Treatment success — CT+Fib versus CT alone. Random-effects meta-analysis of the risk ratio (RR) with 95% confidence intervals. Heterogeneity: I² = 82.1%, τ² = 0.0318, p < 0.0001.

**Supplementary Figure S11.** Complications — CT alone versus Thora. Random-effects meta-analysis of the risk ratio (RR) with 95% confidence intervals. Heterogeneity: I² = 0.0%, τ² = 0, p = 0.8128.

**Supplementary Figure S12.** Duration of drainage (days) — CT alone versus Thora. Random-effects meta-analysis of the mean difference (MD) with 95% confidence intervals. Heterogeneity: I² = 88.5%, τ² = 52.4584, p = 0.0002.

**Supplementary Figure S13.** Fever duration (days) — CT alone versus Thora. Random-effects meta-analysis of the mean difference (MD) with 95% confidence intervals. Heterogeneity: I² = 0.0%, τ² = 0, p = 0.9016.

**Supplementary Figure S14.** Length of stay (days) — CT alone versus Thora. Random-effects meta-analysis of the mean difference (MD) with 95% confidence intervals. Heterogeneity: I² = 91.6%, τ² = 35.1525, p < 0.0001.

**Supplementary Figure S15.** Treatment success — CT alone versus Thora. Random-effects meta-analysis of the risk ratio (RR) with 95% confidence intervals. Heterogeneity: I² = 84.6%, τ² = 0.0087, p < 0.0001.

**Supplementary Figure S16.** Complications — CT alone versus VATS. Random-effects meta-analysis of the risk ratio (RR) with 95% confidence intervals. Heterogeneity: I² = 0.0%, τ² = 0, p = 0.3213.

**Supplementary Figure S17.** Length of stay (days) — CT alone versus VATS. Random-effects meta-analysis of the mean difference (MD) with 95% confidence intervals. Heterogeneity: I² = 88.3%, τ² = 21.0479, p < 0.0001.

**Supplementary Figure S18.** Treatment success — CT alone versus VATS. Random-effects meta-analysis of the risk ratio (RR) with 95% confidence intervals. Heterogeneity: I² = 98.9%, τ² = 1.7700, p < 0.0001.

**Supplementary Figure S19.** Treatment success — Thora versus VATS. Random-effects meta-analysis of the risk ratio (RR) with 95% confidence intervals. Heterogeneity: I² = 64.6%, τ² = 0.0261, p = 0.0373.

**Supplementary Figure S20.** Complications — CT+Fib versus Thora. Random-effects meta-analysis of the risk ratio (RR) with 95% confidence intervals. Heterogeneity: I² = 0.0%, τ² = 0, p = 0.5949.

**Supplementary Figure S21.** Length of stay (days) — CT+Fib versus Thora. Random-effects meta-analysis of the mean difference (MD) with 95% confidence intervals. Heterogeneity: I² = 89.0%, τ² = 56.2145, p < 0.0001.

**Supplementary Figure S22.** Mortality — CT+Fib versus Thora. Random-effects meta-analysis of the risk ratio (RR) with 95% confidence intervals. Heterogeneity: I² = 0.0%, τ² = 0, p = 0.4141.

**Supplementary Figure S23.** Node-splitting: direct, indirect and network estimates for each contrast.

**Supplementary Figure S24.** Net-heat matrices; warm cells would indicate designs contributing to inconsistency.

**Supplementary Figure S25.** Network heterogeneity diagnostics (I² and τ²) by outcome.

**Supplementary Figure S26.** Comparison-adjusted funnel plots for networks with ≥ 10 studies.

**Supplementary Figure S27.** Network estimates versus chest tube alone in the full network (open circles; 40 studies) and in the sensitivity network restricted to primary-line treatment arms (filled squares; 26 studies), for all eight outcomes. k = number of direct studies, full | primary-only. "Not estimable" indicates that the thoracotomy node was disconnected from the primary-only network for that outcome.

# 2 Supplementary Tables

**Supplementary Table S1. Electronic database search strategy**

| **Database** | **Search strategy** |
| --- | --- |
| PubMed | ((“empyema thoracis” OR “pleural empyema” OR “pediatric empyema”) AND (incidence OR epidemiology OR microbiology OR “causative agents” OR VATS OR thoracoscopy OR “intrapleural fibrinolytics” OR urokinase OR “tPA DNase” OR “chest tube drainage”) AND (pediatric OR paediatric OR child OR children OR infant)) |
| Embase | ((“empyema thoracis” OR “pleural empyema” OR “pediatric empyema”) AND (incidence OR epidemiology OR microbiology OR “causative agents” OR VATS OR thoracoscopy OR “intrapleural fibrinolytics” OR urokinase OR “tPA DNase” OR “chest tube drainage”) AND (pediatric OR paediatric OR child OR children OR infant)) |
| Scopus | ((“empyema thoracis” OR “pleural empyema” OR “pediatric empyema”) AND (incidence OR epidemiology OR microbiology OR “causative agents” OR VATS OR thoracoscopy OR “intrapleural fibrinolytics” OR urokinase OR “tPA DNase” OR “chest tube drainage”) AND (pediatric OR paediatric OR child OR children OR infant)) |
| Web of Science | ((“empyema thoracis” OR “pleural empyema” OR “pediatric empyema”) AND (incidence OR epidemiology OR microbiology OR “causative agents” OR VATS OR thoracoscopy OR “intrapleural fibrinolytics” OR urokinase OR “tPA DNase” OR “chest tube drainage”) AND (pediatric OR paediatric OR child OR children OR infant)) |
| Cochrane Central Register of Controlled Trials | ((“empyema thoracis” OR “pleural empyema” OR “pediatric empyema”) AND (incidence OR epidemiology OR microbiology OR “causative agents” OR VATS OR thoracoscopy OR “intrapleural fibrinolytics” OR urokinase OR “tPA DNase” OR “chest tube drainage”) AND (pediatric OR paediatric OR child OR children OR infant)) |

**Supplementary Table S2. Characteristics of the 77 studies contributing to the quantitative synthesis**

| First Author | Country | Study Design | Single/multi-Center | Study Period | N | Male % | Age | Empyema Stage Reported | Microbiology Reported | Treatment Modality |
| --- | --- | --- | --- | --- | --- | --- | --- | --- | --- | --- |
| Ratta 2023 (76) | Italy | Retrospective Cohort | Multicenter | 2004-2021 | 52 | 57.2 | 4.8 (0-16)^a^ | Yes | Yes | Chest tube + Fibrinolytics  VATS |
| Huang 2021 (47) | China | Case Series | Single Center | 2019-2019 | 21 | 57.1 | 4.1 ± 3^b^ | Yes | Yes | VATS |
| Barglik 2021 (28) | Poland | Prospective Cohort | Single Center | 1996-2017 | 61 | 44.4 | 6.6 ± 5.3^b^ | Yes | Yes | VATS |
| Jaiswal 2020 (48) | Nepal | Retrospective Cohort | Single Center | 2016-2019 | 55 | 47.6 | 6 (1-15)^c^ | Yes | Yes | Thoracotomy |
| Pogorelic 2021 (73) | Croatia | Retrospective Cohort | Single Center | 2009-2019 | 15 | 47.6 | 5 (2.8-6)^d^ | Yes | Yes | VATS |
| Livingston 2020 (58) | Canada | Randomised Controlled Trial | Multicenter | 2013-2017 | 48 | 25 | 5.3 ± 3.7^b^ | No | Yes | Chest tube+ Fibrinolytics |
| Yue 2019 (95) | China | Retrospective Cohort | Single Center | 2010-2016 | 89 | 51 | 2.72 ± 0.37^b^ | No | Yes | Chest tube |
| Nandan 2019 (64) | India | Retrospective Cohort | Single Center | 2013-2017 | 84 | 54.5 | 7.1 (3.28-9.41)^d^ | No | Yes | Chest tube + fibrinolytic  Chest tube |
| Shankar 2020 (84) | India | Randomised Controlled Trial | Single Center | 2015-2017 | 48 | 64 | 5 ± 4.34^b^ | Yes | No | Chest tube+ fibrinolytic  VATS |
| Maffey 2019 (59) | Argentina | Prospective Cohort | Single Center | NR | 41 | 63 | 9.7 ± 3.2^b^ | No | Yes | Chest tube  Thoracotomy |
| Knebel 2018 (52) | Brazil | Retrospective Cohort | Single Center | 2000-2011 | 79 | 51.9 | 2.9 (0.2-11.4)^a^ | No | Yes | VATS |
| Parelkar 2017 (60) | India | Retrospective Cohort | Single Center | 2005-2014 | 97 | 63.5 | 0.08-15^e^ | No | No | VATS |
| Segerer 2017 (83) | Germany | Prospective Cohort | Multicenter | 2010-2013 | 645 | 49 | 5 (3-9)^d^ | Yes | Yes | Chest tube  Chest tube + fibrinolytics |
| Espinosa 2016 (41) | USA | Retrospective Cohort | Single Center | 2008-2012 | 61 | 57 | 3.5 (2-7)^f^ | No | Yes | VATS |
| Gayretli-Aydın 2016 (43) | Turkey | Retrospective Cohort | Single Center | 2006-2015 | 70 | 57 | 6 (5.6)^d^ | No | Yes | Chest tube + fibrinolytic  VATS  Thoracotomy |
| Bender 2015 (29) | USA | Retrospective Cohort | Single Center | 2005-2013 | 112 | 52.6 | 5.26 (0.17-17)^a^ | Yes | Yes | VATS |
| Goyal 2014 (44) | India | Prospective Cohort | Single Center | 2010-2013 | 70 | 48.6 | 5.44 (1-14)^a^ | No | Yes | Chest tube  Thoracotomy |
| Gasior 2013 (42) | USA | Retrospective Cohort | Single Center | 2007-2012 | 102 | 52.9 | 6.1 ± 4.7^b^ | Yes | No | Chest tube + Fibrinolytic |
| Kumar 2013 (54) | India | Prospective Cohort | Single Center | 2008-2009 | 25 | 68 | 3 (0.33-11)^d^ | No | Yes | Chest tube |
| Grisaru-Soen 2013 (45) | Israel | Retrospective Cohort | Multicenter | 2000-2009 | 47 | 46.8 | 4.12 (0.75-16.5)^a^ | No | Yes | Chest tube  Chest tube + Fibrinolytic  Thoracotomy |
| Narayanappa 2013 (65) | India | Prospective Cohort | Single Center | 2008-2010 | 50 | 60.7 | 0.17-12^e^ | No | Yes | Chest tube  VATS  Thoracotomy |
| Cobanoglu 2011 (33) | Turkey | Randomized Controlled Trial | Single Center | NR | 54 | 48.1 | 8.02 ± 2.68^b^ | Yes | Yes | Chest tube + Fibrinolytic  VATS |
| Dass 2011 (36) | India | Retrospective Cohort | Single Center | 2006-2010 | 150 | 58.7 | 4.74 ± 3.53^b^ | No | Yes | Chest tube  Chest tube + fibrinolytic  Thoracotomy |
| Plackett 2010 (72) | USA | Retrospective Cohort | Single Center | 2002-2007 | 25 | 36 | 4.6 ± 0.7^b^ | Yes | Yes | Chest tube  VATS |
| Stefanutti 2010 (90) | Italy | Prospective Cohort | Single Center | 2001-2007 | 41 | 44 | 4.4 (3.2–6.9)^d^ | No | Yes | Chest tube + fibrinolytic |
| Chen 2009 (30) | Taiwan | Retrospective Cohort | Single Center | 1995-2008 | 101 | 51.5 | 4.4 ± 2.7^b^ | No | Yes | VATS |
| Demirhan 2008 (38) | Turkey | Retrospective Cohort | Multicenter | 2000-2016 | 681 | 44.3 | 4.8 (0.08-15)^a^ | No | Yes | Chest tube  Chest tube+ fibrinolytics  VATS  Thoracotomy |
| Khalil 2007 (50) | United Kingdom | Retrospective Cohort | Single Center | 2001-2003 | 38 | 65.8 | 4.9 (0.4-15.8)^a^ | No | Yes | Chest tube  Chest tube + fibrinolytic  VATS  Thoracotomy |
| Satpathy 2005 (81) | India | Prospective Cohort | Single Center | 2001 - 2003 | 53 | 49.05 | 4.68 ± 3.34^b^ | No | Yes | Chest tube  Thoracotomy |
| Eroğlu E 2004 (40) | Turkey | Retrospective Cohort | Single Center | NR | 12 | 55.9 | 3.8 (0.3-14)^a^ | No | Yes | Thoracotomy |
| Schultz 2004 (82) | USA | Retrospective Cohort | Single Center | 1993-2002 | 125 | 56.5 | 4 ± 3.6^b^ | No | Yes | VATS |
| Ozcelik 2004 (66) | Turkey | Retrospective Cohort | Single Center | 1990-2002 | 486 | 56 | 4.7 (0.05-15)^a^ | Yes | Yes | Chest tube  Chest tube + fibrinolytic  Thoracotomy |
| Knudtson 2004 (53) | USA | Retrospective Cohort | Single Center | 1997-2001 | 22 | 63.6 | 4.1 (0.66-12.6)^a^ | No | Yes | VATS |
| Baranwal 2003 (27) | India | Retrospective Cohort | Single Center | 1989-1998 | 243 | 70.6 | <1 y: 23.5%,  1–5 y: 43%,  >5 y: 33%^g^ | No | Yes | Chest tube  Thoracotomy |
| Cohen 2003 (34) | United Kingdom | Retrospective Cohort | Single Center | 1989-1997 + 2000-2001 | 75 | 57.2 | 5.90 ± 2.82^h^ | No | Yes | Chest tube  VATS |
| Shoseyov 2002 (86) | Israel | Prospective Cohort | Multicenter | 1992-1999 | 67 | 59.7 | 5 ± 4.7^b^ | No | No | Chest tube |
| Meier 2000 (62) | USA | Retrospective Cohort | Single Center | 1995-1999 | 31 | 54.8 | 5.1 (0.92-17)^a^ | No | Yes | Chest tube+ fibrinolytic  VATS |
| Shankar 2000 (85) | United Kingdom | Retrospective Cohort | Single Center | 1980-1997 | 47 | 63.8 | 7 (2-16)^c^ | No | Yes | Thoracotomy |
| Haapanen 2026 (46) | Finland | Retrospective Cohort | Single Center | 2018-2024 | 87 | 42.5 | 3.9 ( 0.7-17.3)^c^ | No | Yes | Chest tube + Fibrinolytic  VATS |
| Agrawal 2021 (21) | India | Retrospective Cohort | Single Center | 2012-2019 | 93 | 59.1 | 4 (3-5)^d^ | No | No | Thoracotomy |
| Singh 2020 (88) | India | Retrospective Cohort | Single Center | 2018-2020 | 15 | 40 | < 1: 6.66% 1 - 5 y: 66.66% 5 - 10: 20% > 10: 6.66%^g^ | Yes | No | VATS  Chest tube |
| Sehitogullari 2019 (7) | Turkey | Retrospective Cohort | Multicenter | 2000-2016 | 681 | 55.7 | 4.8 (0.08-15)^a^ | No | Yes | Chest tube |
| Lamas-Pinheiro 2016 (56) | Portugal | Retrospective Cohort | Single Center | 2006-2014 | 91 | 58.2 | 5.2 ± 3.7^b^ | No | No | VATS |
| Pandey 2015 (68) | India | Prospective Cohort | Single Center | 2011-2013 | 28 | 53.5 | 7.32 ± 2.42^b^ | Yes | No | Thoracotomy |
| Sahin 2013 (78) | Turkey | Retrospective Cohort | Single Center | 2002-2012 | 330 | 55.2 | 4.3 (1.25-15)^d^ | No | Yes | Thoracotomy  Chest tube + fibrinolytic |
| Roxburgh 2007 (77) | United Kingdom | Retrospective Cohort | Single Center | 1990-2006 | 28 | 50.00 | 6.8 (1-13)^a^ | No | Yes | Thoracotomy  VATS |
| Ulku 2004 (93) | Turkey | Retrospective Cohort | Single Center | 1994-2002 | 78 | 61.50 | 5 ± 3.99^b^ | Yes | Yes | Chest tube + fibrinolytics |
| Ozcelik 2003 (67) | Turkey | Retrospective Cohort | Single Center | 1994-2002 | 72 | 59.72 | 5 (0.41-15)^a^ | No | Yes | Chest tube + fibrinolytics |
| Kılıç 2002 (51) | Turkey | Retrospective Cohort | Single Center | 1998-2000 | 25 | 76 | 4.2 (1-12)^a^ | No | No | Chest tube + fibrinolytics |
| Proesmans 2013 (74) | Belgium | Retrospective Cohort | Single Center | 2006-2012 | 132 | 59.8 | 3.7 (2.5-5.4)^a^ | No | Yes | Chest tube + fibrinolytic  VATS  Chest tube |
| St. Peter 2008 (89) | USA | Randomized Controlled Trial | Single Center | 2006-1007 | 36 | NR | 5.0 ± 4.19^b^ | No | Yes | VATS  Chest tube + fibrinolytic |
| Livingston 2016 (57) | Uk | Retrospective Cohort | Single Center | 2005-2013 | 67 | 41.7 | 5.72 (0.167-17)^a^ | Yes | No | VATS  Chest tube + fibrinolytic |
| Mathew 2018 (61) | India | Retrospective Cohort | Single Center | 2013-2017 | 149 | NR | **3.1 (1.3–6.0)**^d^ | No | No | Chest tube + fibrinolytic  Chest tube |
| Pereira 2017 (71) | Brazil | Retrospective Cohort | Single Center | 2000-2013 | 60 | 46.7 | 3 (0-14)^d^ | No | No | VATS |
| Satish 2003 (80) | United kingdom | Retrospective Cohort | Single Center | 1996-1998 | 14 | 50 | 5 (2-14)^d^ | No | Yes | Chest tube |
| Chen 2002 (31) | America | Retrospective Cohort | Single Center | 1990-2000 | 70 | 31.6% | 6 (1-17)^c^ | Yes | Yes | Chest tube |
| Ekingen 2004 (39) | Turkey | Retrospective Cohort | Single Center | 2000-NR | 32 | 52.6 | 3.4 (0.42-10)^a^ | Yes | Yes | Chest tube + fibrinolytic |
| Zampoli 2015 (96) | South Africa | Retrospective Cohort | Single Center | 2006-2011 | 99 | 57 | 1.5 (0.7–3.6)^d^ | No | No | Chest tube + fibrinolytic  Chest tube |
| Aydoğan 2008 (25) | Turkey | Retrospective Cohort | Single Center | 1999-2002 | 53 | 54.7 | 4.16 ± 3.63^b^ | Yes | Yes | Chest tube  Chest tube + fibrinolytics |
| Tander 2007 (92) | Turkey | Retrospective Cohort | Single Center | 2002-2006 | 12 | 58.3 | 4.5 (0.24-14)^c^ | No | No | VATS |
| Mondal 2025 (63) | India | Prospective Cohort | Single Center | 2023-2024 | 100 | 56.9 | 5.42 ± 2.65^b^ | No | Yes | Chest tube  Chest tube + fibrinolytics |
| Ranjan Kunar 2024 (75) | India | Prospective Cohort | Single Center | 2016-2018 | 50 | 54 | 0.83-12^e^ | No | No | Chest tube  Chest tube +fibrinolytics |
| Darpan Jakkal 2024 (35) | India | Prospective Cohort | Single Center | 2010-2012 | 30 | 80 | 4.4 (0-12)^a^ | No | No | Chets tube + fibrinolytics  VATS |
| Ateş 2023 (24) | Turkey | Case Series | Single Center | NR | 34 | 70.6 | 5.62 (0.7-17.5)^a^ | Yes | Yes | VATS |
| Sarafi 2023 (79) | Iran | Prospective Cohort | Single Center | 2015-2022 | 31 | 41.9 | 6.25 ± 4.8^b^ | Yes | No | Chest tube + fibrinolytics |
| Kumar 2022 (55) | India | Prospective Cohort | Single Center | 2018-2019 | 130 | 67.7 | 4.75 (0.17-12)^a^ | No | Yes | Chest tube |
| De Benedictis 2019 (37) | Italy | Prospective Cohort | Single Center | 2003-2014 | 39 | 61.5 | 4.6 (0.92-15)^c^ | No | Yes | VATS  Chest tube |
| Singh 2018 (87) | India | Retrospective Cohort | Single Center | 2015-2016 | 100 | 65 | 4.5 (0.17-13)^a^ | Yes | No | Thoracotomy |
| Almaramhy 2015 (23) | Saudi Arabia | Retrospective Cohort | Single Center | 2007-2012 | 62 | 40.3 | 3.77 ± 2.99^b^ | No | Yes | Chest tube  Thoracotomy |
| Van Loo 2014 (94) | Australia | Retrospective Cohort | Single Center | 1998-2009 | 60 | 58.3 | 4.70 ± 4.30^b^ | No | No | Chest tube + fibrinolytics  VATS |
| Marhuenda 2014 (60) | Spain | Randomized Controlled Trial | Multicenter | 2008-2010 | 103 | 59 | 4.4 (1-13.7)^a^ | No | Yes | VATS  Chest tube + fibrinolytics |
| Pappalardo 2009 (69) | Belgium | Case Series | Single Center | 2003-2007 | 23 | NR | 4.9 (0.25-15)^a^ | No | Yes | VATS |
| Suchar 2006 (91) | USA | Retrospective Cohort | Single Center | 2001-2005 | 42 | 50 | 5.9 (0.3-17)^a^ | Yes | Yes | VATS |
| Bailey 2005 (26) | Canada | Retrospective Cohort | Single Center | 1991-2003 | 58 | 58.6 | 6 (0.33-17)^c^ | No | Yes | Chest tube  VATS  Thoracotomy |
| Alexiou 2003 (22) | United Kingdom | Retrospective Cohort | Single Center | 1993-2002 | 44 | 50 | 8 (0.17-16)^c^ | Yes | Yes | Thoracotomy |
| Kercher 2000 (49) | USA | Case Series | Single Center | 1997-1998 | 16 | 56.3 | 5 (1-16)^a^ | No | Yes | VATS  Chest tube |
| Chiu 2006 (32) | Taiwan | Retrospective Cohort | Single Center | 2000-2004 | 81 | 50.6 | 4 (1.2-16.5)^c^ | Yes | Yes | Chest tube  Chest tube + fibrinolytics  VATS  Thoracotomy |

NR, not reported; N, total sample size; USA, United States of America.

^a^ mean (range)

^b^ mean ± standard deviation

^c^ median (range)

^d^ median (interquartile range)

^e^ range (years)

^f^ mean (interquartile range)

^g^ range (%)

^h^ mean ± SEM

**Supplementary Table S3. Potentially relevant randomized studies excluded following full-text assessment**

| **Study** | **Reason for exclusion** |
| --- | --- |
| Kurt 2006 (106) | Mixed Stage I and Stage II population without separately extractable outcomes for the eligible Stage II population. Enrollment was based on a large parapneumonic effusion requiring evacuation rather than restriction to fibrinopurulent/organizing empyema. The authors explicitly state that their population included Stage I or II parapneumonic effusions, while the review prespecified inclusion of Stage II/III disease only. Although loculations were present in many participants, treatment outcomes were not reported separately for the eligible Stage II subgroup. |
| Sonnappa 2006 (100) | Mixed Stage I–III population without sufficiently extractable treatment outcomes restricted to the eligible Stage II/III population. Sixteen of the 60 randomized children had Stage I disease. Overall treatment outcomes were reported for the complete randomized groups and therefore included ineligible Stage I patients. |
| Thakur 2025 (107) | Did not compare distinct treatment modalities represented in the prespecified treatment framework. All randomized participants had already received CT drainage plus three doses of intrapleural streptokinase; randomization then compared three additional streptokinase doses with placebo. Thus, both randomized groups belonged to the same prespecified treatment category (CT + fibrinolytics), and the trial evaluated fibrinolytic dosing rather than comparative effectiveness between the review’s treatment strategies. |

**Supplementary Table S4. Definitions of treatment success across included studies**

| **Study** | **Definition of treatment success, as reported** |
| --- | --- |
| ***Composite (n = 11)*** | |
| *Barglik 2021* | Resolution by CXR, no need for treatment escalation |
| *Marhuenda 2014* | No fever ≥38°C >4 days and no persistent pleural collection on ultrasound and not needing further procedures |
| *Dass 2011* | Full lung expansion; no escalation from chest tube |
| *Pappalardo 2009* | Clinically stable and symptom-free + CXR improvement at 4-week follow-up |
| *Eroğlu E 2004* | Clinical and radiological |
| *Jaiswal 2020* | Good clinical condition, satisfactory CXR |
| *Nandan 2019* | No need for surgery (no persistent symptoms or pus drainage, & FU US showing adequate drainage) |
| *Ratta 2023* | Defervescence and radiographic resolution by CXR, no need for treatment escalation |
| *Shankar 2020* | Clinical improvement in fever/respiratory distress + radiographic clearance on CXR/US, no subsequent intervention |
| *Stefanutti 2010* | Clinical improvement after intrapleural urokinase with adequate fluid evacuation and CXR clearance |
| *Yue 2019* | Normal temp/WBC/CRP + imaging resolution, no repeat surgery |
| ***Clinical resolution (n = 2)*** | |
| *Shoseyov 2002* | Clinically |
| *Pandey 2015* | Clinically |
| ***Radiographic clearance (n = 5)*** | |
| *Ozcelik 2003* | Response to treatment, radiological clearance |
| *Cobanoglu 2011* | Lungs found to be fully expanded = success |
| *Huang 2021* | CXR shows no pneumothorax or complications |
| *Kilic 2002* | Resolution on imaging |
| *Ulku 2004* | Response to treatment, radiological clearance |
| ***No further surgery/procedure (n = 13)*** | |
| *Alexiou 2003* | 40/44 showed resolution without need for further surgery |
| *Loo 2014* | No need for subsequent surgical intervention, consisting of either VATS or decortication |
| *Bender 2015* | Not requiring reoperation or readmission |
| *Chiu 2006* | Managed successfully without tube drainage |
| *Espinosa 2016* | No second surgery required |
| *Gasior 2013* | Not required subsequent VATS |
| *Goyal 2014* | Not requiring further decortication |
| *Khalil 2007* | Not requiring second procedure |
| *Knebel 2018* | No need for any additional surgical procedure after VATS |
| *Livingston 2020* | Not needing additional pleural drainage procedures after the initial treatment |
| *Parelkar 2017* | No need for repeat surgery |
| *Pogorelic 2021* | No need for further surgery |
| *Suchar 2006* | VATD completed; no return to OR, no recurrence on follow-up |
| ***No escalation (n = 12)*** | |
| *Sahin 2013* | No escalation or complications |
| *Roxburgh 2007* | No escalation required |
| *Chen 2009* | Not requiring escalation |
| *Demirham 2008* | Not requiring escalation |
| *Ekingen 2004* | No escalation |
| *Mathew 2018* | No escalation required |
| *Zampoli 2015* | No escalation required |
| *Proesmans 2013* | No escalation required |
| *Livingston 2016* | No escalation required |
| *Narayanappa 2013* | No escalation after chest tube drainage |
| *Plackett 2010* | Not requiring escalation |
| *St. Peter 2008* | No escalation (incl. ventilation support) required |
| ***Other/unclear (n = 1)*** | |
| *Kumar 2013* | Responding to management |
| ***Not stated (n = 11)*** | |
| *Sehitogullari 2019* | Not stated |
| *Meier 2000* | Not stated |
| *Satish 2003* | Not stated |
| *Ozcelik 2004* | Not stated |
| *Cohen 2003* | Not stated |
| *Haapanen 2026* | Not stated |
| *Knudtson 2004* | Not stated |
| *Chen 2002* | Not stated |
| *Ranjan 2024* | Not stated |
| *Singh 2020* | Not stated |
| *Satpathy 2005* | Not stated |

*55 studies reported treatment success as an outcome. Definitions are reproduced as extracted (lightly condensed for length; patient-count detail omitted). Classification is based on the reported criterion: Composite = ≥2 domains (clinical, radiographic, and/or absence of further intervention); No further surgery/procedure and No escalation = avoidance of a second intervention only; Not stated = success counted but no definition given. The absence of a uniform definition is a source of clinical heterogeneity for this outcome.*

**Supplementary Table S5. Domain-level risk-of-bias judgements.** Panel A: ROBINS-I, non-randomized studies (n = 69). Panel B: RoB 2, randomised trials (n = 5). Values are numbers of studies (%).

**Panel A. Non-randomised studies (ROBINS-I)**

| **Domain** | **Low** | **Moderate** | **Serious** | **Critical** |
| --- | --- | --- | --- | --- |
| D1 Confounding | 0 | 1 (1) | 49 (71) | 19 (28) |
| D2 Selection of participants | 5 (7) | 48 (70) | 16 (23) | 0 |
| D3 Classification of interventions | 60 (87) | 8 (12) | 1 (1) | 0 |
| D4 Deviations from intended interventions | 9 (13) | 20 (29) | 40 (58) | 0 |
| D5 Missing data | 36 (52) | 32 (46) | 1 (1) | 0 |
| D6 Measurement of outcomes | 24 (35) | 45 (65) | 0 | 0 |
| D7 Selection of reported result | 7 (10) | 62 (90) | 0 | 0 |
| **Overall judgement** | **0** | **1 (1)** | **49 (71)** | **19 (28)** |

**Panel B. Randomized trials (RoB 2)**

| **Domain** | **Low** | **Some concerns** | **High** |
| --- | --- | --- | --- |
| D1 Randomization process | 2 (40) | 3 (60) | 0 |
| D2 Deviations from intended interventions | 1 (20) | 4 (80) | 0 |
| D3 Missing outcome data | 5 (100) | 0 | 0 |
| D4 Measurement of the outcome | 5 (100) | 0 | 0 |
| D5 Selection of the reported result | 1 (20) | 4 (80) | 0 |
| **Overall judgement** | **1 (20)** | **4 (80)** | **0** |

*ROBINS-I, Risk Of Bias In Non-randomised Studies of Interventions; RoB 2, Cochrane risk-of-bias tool for randomised trials. Overall judgement is the worst domain judgement for each study. Percentages may not sum to 100 owing to rounding. Single-arm case series (n = 4) were appraised with the JBI checklist and are reported in Supplementary Table S1.*

**Supplementary Table S6. Risk-of-bias domain of the GRADE assessment for each pooled outcome-by-comparison analysis.** k, number of contributing studies.

| **Outcome** | **Comparison** | **k** | **n at serious risk** | **n at critical risk** | **Risk-of-bias judgement** | **Certainty downgrade** |
| --- | --- | --- | --- | --- | --- | --- |
| **Length of hospital stay** | Chest tube vs chest tube + fibrinolytics | 12 | 7 | 5 | Serious | −1 level |
|  | Chest tube vs VATS | 9 | 4 | 5 | Very serious | −2 levels |
|  | Chest tube vs thoracotomy | 11 | 5 | 6 | Very serious | −2 levels |
|  | Chest tube + fibrinolytics vs VATS | 9 | 4 | 1 | Serious | −1 level |
|  | Chest tube + fibrinolytics vs thoracotomy | 4 | 2 | 2 | Very serious | −2 levels |
|  | VATS vs thoracotomy | 4 | 1 | 3 | Very serious | −2 levels |
| **Duration of pleural drainage** | Chest tube vs chest tube + fibrinolytics | 5 | 2 | 3 | Very serious | −2 levels |
|  | Chest tube vs VATS | 3 | 1 | 2 | Very serious | −2 levels |
|  | Chest tube vs thoracotomy | 3 | 2 | 1 | Serious | −1 level |
|  | Chest tube + fibrinolytics vs VATS | 3 | 1 | 0 | Serious | −1 level |
|  | Chest tube + fibrinolytics vs thoracotomy | 1 | 0 | 1 | Very serious | −2 levels |
|  | VATS vs thoracotomy | 1 | 0 | 1 | Very serious | −2 levels |
| **Duration of antibiotics** | Chest tube vs chest tube + fibrinolytics | 3 | 3 | 0 | Serious | −1 level |
|  | Chest tube vs VATS | 4 | 2 | 2 | Very serious | −2 levels |
|  | Chest tube vs thoracotomy | 2 | 2 | 0 | Serious | −1 level |
|  | Chest tube + fibrinolytics vs VATS | 2 | 2 | 0 | Serious | −1 level |
|  | Chest tube + fibrinolytics vs thoracotomy | 1 | 1 | 0 | Serious | −1 level |
|  | VATS vs thoracotomy | 1 | 1 | 0 | Serious | −1 level |
| **Duration of fever** | Chest tube vs chest tube + fibrinolytics | 4 | 1 | 3 | Very serious | −2 levels |
|  | Chest tube vs VATS | 4 | 2 | 2 | Very serious | −2 levels |
|  | Chest tube vs thoracotomy | 2 | 1 | 1 | Very serious | −2 levels |
|  | Chest tube + fibrinolytics vs VATS | 4 | 0 | 1 | Serious | −1 level |
|  | Chest tube + fibrinolytics vs thoracotomy | 1 | 0 | 1 | Very serious | −2 levels |
| **Treatment success** | Chest tube vs chest tube + fibrinolytics | 11 | 5 | 6 | Very serious | −2 levels |
|  | Chest tube vs VATS | 7 | 3 | 4 | Very serious | −2 levels |
|  | Chest tube vs thoracotomy | 7 | 2 | 5 | Very serious | −2 levels |
|  | Chest tube + fibrinolytics vs VATS | 12 | 5 | 3 | Serious | −1 level |
|  | Chest tube + fibrinolytics vs thoracotomy | 6 | 1 | 5 | Very serious | −2 levels |
|  | VATS vs thoracotomy | 4 | 1 | 3 | Very serious | −2 levels |
| **Escalation to further intervention** | Chest tube vs chest tube + fibrinolytics | 8 | 6 | 2 | Serious | −1 level |
|  | Chest tube vs VATS | 7 | 4 | 3 | Serious | −1 level |
|  | Chest tube vs thoracotomy | 5 | 3 | 2 | Serious | −1 level |
|  | Chest tube + fibrinolytics vs VATS | 9 | 5 | 1 | Serious | −1 level |
|  | Chest tube + fibrinolytics vs thoracotomy | 4 | 1 | 3 | Very serious | −2 levels |
|  | VATS vs thoracotomy | 2 | 1 | 1 | Very serious | −2 levels |
| **Complications** | Chest tube vs chest tube + fibrinolytics | 7 | 4 | 3 | Serious | −1 level |
|  | Chest tube vs VATS | 5 | 2 | 3 | Very serious | −2 levels |
|  | Chest tube vs thoracotomy | 6 | 2 | 4 | Very serious | −2 levels |
|  | Chest tube + fibrinolytics vs VATS | 10 | 3 | 2 | Serious | −1 level |
|  | Chest tube + fibrinolytics vs thoracotomy | 3 | 0 | 3 | Very serious | −2 levels |
|  | VATS vs thoracotomy | 3 | 0 | 3 | Very serious | −2 levels |
| **Mortality** | Chest tube vs chest tube + fibrinolytics | 10 | 5 | 5 | Very serious | −2 levels |
|  | Chest tube vs VATS | 8 | 4 | 4 | Very serious | −2 levels |
|  | Chest tube vs thoracotomy | 10 | 6 | 4 | Serious | −1 level |
|  | Chest tube + fibrinolytics vs VATS | 9 | 4 | 2 | Serious | −1 level |
|  | Chest tube + fibrinolytics vs thoracotomy | 7 | 3 | 4 | Very serious | −2 levels |
|  | VATS vs thoracotomy | 6 | 2 | 4 | Very serious | −2 levels |

*VATS, video-assisted thoracoscopic surgery. A cell was rated at very serious risk of bias where at least half of the contributing studies were at critical risk, and at serious risk where any contributing study was at critical risk or at least one was at serious risk. Very serious risk led to a two-level downgrade of certainty, serious risk to a one-level downgrade. All 47 pooled analyses were downgraded; none reached low risk of bias.*

**Supplementary Table S7.** **GRADE certainty of evidence for each pooled outcome-by-comparison analysis. k**, number of contributing studies; n, total participants; I², heterogeneity; RCTs, randomized controlled trials; LOS, length of stay; ROB, risk of bias; ↓, downgrade. Certainty was rated from a starting level of high and downgraded across five domains; indirectness was rated not serious throughout and is therefore not tabulated separately. The 39 rows correspond to the pooled analyses; the eight further cells listed in Supplementary Table S6 could not be pooled and were not rated.

| **Outcome** | **Comparison** | **Measure** | **Studies (k)** | **Participants** | **Effect** | **p** | **I²** | **RoB** | **↓RoB** | **Inconsistency** | **↓Inconsistency** | **Indirectness** | **Imprecision** | **↓Imprecision** | **Publication bias** | **↓Publication bias** | **Total downgrades** | **Certainty of evidence** | **RCTs (k)** | **Certainty (OIS-conservative variant)** |
| --- | --- | --- | --- | --- | --- | --- | --- | --- | --- | --- | --- | --- | --- | --- | --- | --- | --- | --- | --- | --- |
| Treatment success | CT alone vs CT with fibrinolytics | RR | 11 | 1712 | 0.994 [0.871, 1.135] | 0.931 | 82% | Very serious | 2 | very serious (considerable I²) | 2 | not serious | serious (CI includes null) | 1 | serious (Egger p<0.05) | 1 | 6 | Very low | 0 | Very low |
| Treatment success | CT alone vs Thoracotomy | RR | 7 | 926 | 0.921 [0.842, 1.008] | 0.074 | 85% | Very serious | 2 | very serious (considerable I²) | 2 | not serious | serious (CI includes null) | 1 | undetected (k<10, not assessable) | 0 | 5 | Very low | 0 | Very low |
| Treatment success | CT alone vs VATS | RR | 7 | 625 | 0.609 [0.213, 1.743] | 0.355 | 99% | Very serious | 2 | very serious (considerable I²) | 2 | not serious | serious (CI includes null) | 1 | undetected (k<10, not assessable) | 0 | 5 | Very low | 0 | Very low |
| Treatment success | CT with fibrinolytics vs Thoracotomy | RR | 6 | 755 | 0.883 [0.706, 1.103] | 0.271 | 93% | Very serious | 2 | very serious (considerable I²) | 2 | not serious | serious (CI includes null) | 1 | undetected (k<10, not assessable) | 0 | 5 | Very low | 0 | Very low |
| Treatment success | CT with fibrinolytics vs VATS | RR | 12 | 813 | 0.91 [0.847, 0.978] | 0.01 | 36% | Serious | 1 | not serious | 0 | not serious | not serious | 0 | not serious | 0 | 1 | Moderate | 4 | Moderate |
| Treatment success | VATS vs Thoracotomy | RR | 4 | 314 | 0.921 [0.742, 1.143] | 0.455 | 65% | Very serious | 2 | serious (substantial I²) | 1 | not serious | serious (CI includes null) | 1 | undetected (k<10, not assessable) | 0 | 4 | Very low | 0 | Very low |
| Treatment escalation | CT alone vs CT with fibrinolytics | RR | 8 | 679 | 2.483 [1.263, 4.883] | 0.008 | 72% | Serious | 1 | serious (substantial I²) | 1 | not serious | not serious | 0 | undetected (k<10, not assessable) | 0 | 2 | Low | 0 | Low |
| Treatment escalation | CT alone vs Thoracotomy | RR | 4 | 282 | 5.961 [1.483, 23.953] | 0.012 | 0% | Serious | 1 | not serious | 0 | not serious | not serious (CI excludes null; small sample) | 0 | undetected (k<10, not assessable) | 0 | 1 | Moderate | 0 | Moderate |
| Treatment escalation | CT alone vs VATS | RR | 7 | 244 | 4.806 [1.549, 14.91] | 0.007 | 32% | Serious | 1 | not serious | 0 | not serious | not serious (CI excludes null; small sample) | 0 | undetected (k<10, not assessable) | 0 | 1 | Moderate | 0 | Moderate |
| Treatment escalation | CT with fibrinolytics vs Thoracotomy | RR | 4 | 637 | 6.159 [0.488, 77.704] | 0.16 | 86% | Very serious | 2 | very serious (considerable I²) | 2 | not serious | serious (CI includes null) | 1 | undetected (k<10, not assessable) | 0 | 5 | Very low | 0 | Very low |
| Treatment escalation | CT with fibrinolytics vs VATS | RR | 9 | 687 | 3.179 [1.452, 6.96] | 0.004 | 25% | Serious | 1 | not serious | 0 | not serious | not serious | 0 | undetected (k<10, not assessable) | 0 | 1 | Moderate | 3 | Moderate |
| Treatment escalation | VATS vs Thoracotomy | RR | 2 | 33 | 4.774 [0.588, 38.787] | 0.144 | 0% | Very serious | 2 | not serious | 0 | not serious | serious (CI includes null) | 1 | undetected (k<10, not assessable) | 0 | 3 | Very low | 0 | Very low |
| Mortality | CT alone vs CT with fibrinolytics | RR | 5 | 1330 | 0.704 [0.185, 2.686] | 0.608 | 29% | Very serious | 2 | not serious | 0 | not serious | serious (CI includes null) | 1 | undetected (k<10, not assessable) | 0 | 3 | Very low | 0 | Very low |
| Mortality | CT alone vs Thoracotomy | RR | 4 | 629 | 0.175 [0.036, 0.843] | 0.03 | 9% | Serious | 1 | not serious | 0 | not serious | not serious | 0 | undetected (k<10, not assessable) | 0 | 1 | Moderate | 0 | Moderate |
| Mortality | CT with fibrinolytics vs Thoracotomy | RR | 3 | 606 | 0.336 [0.096, 1.175] | 0.088 | 0% | Very serious | 2 | not serious | 0 | not serious | serious (CI includes null) | 1 | undetected (k<10, not assessable) | 0 | 3 | Very low | 0 | Very low |
| Complications | CT alone vs CT with fibrinolytics | RR | 6 | 791 | 0.49 [0.208, 1.156] | 0.104 | 74% | Serious | 1 | serious (substantial I²) | 1 | not serious | serious (CI includes null) | 1 | undetected (k<10, not assessable) | 0 | 3 | Very low | 0 | Very low |
| Complications | CT alone vs Thoracotomy | RR | 5 | 548 | 1.3 [0.675, 2.502] | 0.432 | 0% | Very serious | 2 | not serious | 0 | not serious | serious (CI includes null) | 1 | undetected (k<10, not assessable) | 0 | 3 | Very low | 0 | Very low |
| Complications | CT alone vs VATS | RR | 2 | 199 | 1.758 [0.492, 6.288] | 0.385 | 0% | Very serious | 2 | not serious | 0 | not serious | serious (CI includes null) | 1 | undetected (k<10, not assessable) | 0 | 3 | Very low | 0 | Very low |
| Complications | CT with fibrinolytics vs Thoracotomy | RR | 3 | 500 | 1.39 [0.66, 2.928] | 0.386 | 0% | Very serious | 2 | not serious | 0 | not serious | serious (CI includes null) | 1 | undetected (k<10, not assessable) | 0 | 3 | Very low | 0 | Very low |
| Complications | CT with fibrinolytics vs VATS | RR | 9 | 504 | 1.146 [0.722, 1.818] | 0.564 | 0% | Serious | 1 | not serious | 0 | not serious | serious (CI includes null) | 1 | undetected (k<10, not assessable) | 0 | 2 | Low | 4 | Low |
| Complications | VATS vs Thoracotomy | RR | 2 | 79 | 4.577 [1.418, 14.772] | 0.011 | 0% | Very serious | 2 | not serious | 0 | not serious | not serious (CI excludes null; small sample) | 0 | undetected (k<10, not assessable) | 0 | 2 | Low | 0 | Very low |
| LOS (days) | CT alone vs CT with fibrinolytics | MD | 12 | 1130 | 1.332 [-1.695, 4.359] | 0.389 | 88% | Serious | 1 | very serious (considerable I²) | 2 | not serious | serious (CI includes null) | 1 | not serious | 0 | 4 | Very low | 0 | Very low |
| LOS (days) | CT alone vs Thoracotomy | MD | 11 | 900 | -1.415 [-5.312, 2.482] | 0.477 | 92% | Very serious | 2 | very serious (considerable I²) | 2 | not serious | serious (CI includes null) | 1 | not serious | 0 | 5 | Very low | 0 | Very low |
| LOS (days) | CT alone vs VATS | MD | 9 | 311 | 2.533 [-1.005, 6.07] | 0.161 | 88% | Very serious | 2 | very serious (considerable I²) | 2 | not serious | serious (CI includes null) | 1 | undetected (k<10, not assessable) | 0 | 5 | Very low | 0 | Very low |
| LOS (days) | CT with fibrinolytics vs Thoracotomy | MD | 4 | 208 | 2.077 [-5.713, 9.866] | 0.601 | 89% | Very serious | 2 | very serious (considerable I²) | 2 | not serious | serious (CI includes null) | 1 | undetected (k<10, not assessable) | 0 | 5 | Very low | 0 | Very low |
| LOS (days) | CT with fibrinolytics vs VATS | MD | 9 | 493 | 0.456 [-1.243, 2.156] | 0.599 | 84% | Serious | 1 | very serious (considerable I²) | 2 | not serious | serious (CI includes null) | 1 | undetected (k<10, not assessable) | 0 | 4 | Very low | 3 | Very low |
| LOS (days) | VATS vs Thoracotomy | MD | 4 | 92 | -2.332 [-5.316, 0.651] | 0.126 | 22% | Very serious | 2 | not serious | 0 | not serious | serious (CI includes null) | 1 | undetected (k<10, not assessable) | 0 | 3 | Very low | 0 | Very low |
| Pleural drainage duration (days) | CT alone vs CT with fibrinolytics | MD | 5 | 719 | 0.743 [-1.716, 3.201] | 0.554 | 83% | Very serious | 2 | very serious (considerable I²) | 2 | not serious | serious (CI includes null) | 1 | undetected (k<10, not assessable) | 0 | 5 | Very low | 0 | Very low |
| Pleural drainage duration (days) | CT alone vs Thoracotomy | MD | 3 | 197 | -3.607 [-12.376, 5.161] | 0.42 | 88% | Serious | 1 | very serious (considerable I²) | 2 | not serious | serious (CI includes null) | 1 | undetected (k<10, not assessable) | 0 | 4 | Very low | 0 | Very low |
| Pleural drainage duration (days) | CT alone vs VATS | MD | 3 | 135 | 3.016 [-4.75, 10.782] | 0.447 | 92% | Very serious | 2 | very serious (considerable I²) | 2 | not serious | serious (CI includes null) | 1 | undetected (k<10, not assessable) | 0 | 5 | Very low | 0 | Very low |
| Pleural drainage duration (days) | CT with fibrinolytics vs VATS | MD | 3 | 220 | 1.11 [0.876, 1.345] | < 0.001 | 0% | Serious | 1 | not serious | 0 | not serious | not serious (CI excludes null; small sample) | 0 | undetected (k<10, not assessable) | 0 | 1 | Moderate | 1 | Low |
| Antibiotic duration (days) | CT alone vs CT with fibrinolytics | MD | 3 | 122 | 1.971 [-2.846, 6.789] | 0.423 | 84% | Serious | 1 | very serious (considerable I²) | 2 | not serious | serious (CI includes null) | 1 | undetected (k<10, not assessable) | 0 | 4 | Very low | 0 | Very low |
| Antibiotic duration (days) | CT alone vs Thoracotomy | MD | 2 | 65 | 4.106 [0.473, 7.738] | 0.027 | 0% | Serious | 1 | not serious | 0 | not serious | not serious (CI excludes null; small sample) | 0 | undetected (k<10, not assessable) | 0 | 1 | Moderate | 0 | Low |
| Antibiotic duration (days) | CT alone vs VATS | MD | 4 | 146 | 5.596 [-0.842, 12.035] | 0.088 | 85% | Very serious | 2 | very serious (considerable I²) | 2 | not serious | serious (CI includes null) | 1 | undetected (k<10, not assessable) | 0 | 5 | Very low | 0 | Very low |
| Antibiotic duration (days) | CT with fibrinolytics vs VATS | MD | 2 | 113 | 0.3 [-0.133, 0.733] | 0.174 | 0% | Serious | 1 | not serious | 0 | not serious | serious (CI includes null) | 1 | undetected (k<10, not assessable) | 0 | 2 | Low | 0 | Low |
| Fever duration (days) | CT alone vs CT with fibrinolytics | MD | 4 | 394 | 1.623 [-2.433, 5.68] | 0.433 | 91% | Very serious | 2 | very serious (considerable I²) | 2 | not serious | serious (CI includes null) | 1 | undetected (k<10, not assessable) | 0 | 5 | Very low | 0 | Very low |
| Fever duration (days) | CT alone vs Thoracotomy | MD | 2 | 158 | 0.067 [-1.995, 2.129] | 0.949 | 0% | Very serious | 2 | not serious | 0 | not serious | serious (CI includes null) | 1 | undetected (k<10, not assessable) | 0 | 3 | Very low | 0 | Very low |
| Fever duration (days) | CT alone vs VATS | MD | 4 | 120 | 2.96 [0.094, 5.827] | 0.043 | 95% | Very serious | 2 | very serious (considerable I²) | 2 | not serious | not serious (CI excludes null; small sample) | 0 | undetected (k<10, not assessable) | 0 | 4 | Very low | 0 | Very low |
| Fever duration (days) | CT with fibrinolytics vs VATS | MD | 4 | 198 | 0.953 [0.018, 1.889] | 0.046 | 0% | Serious | 1 | not serious | 0 | not serious | not serious (CI excludes null; small sample) | 0 | undetected (k<10, not assessable) | 0 | 1 | Moderate | 2 | Moderate |

**Supplementary Table S8. Primary-treatment-only sensitivity network meta-analysis compared with the principal network.** Panel A: estimates versus chest tube alone and all other contrasts (RR or MD with 95% CI), number of contributing studies, and whether direction and statistical significance were unchanged. Panel B: per-outcome network composition (studies, contrasts, nodes, τ², I²). Not estimable = thoracotomy node disconnected from the primary-only network.

**Panel A. Network estimates: full network versus primary-only network**

| **Outcome** | **Comparison** | **Measure** | **Full network: estimate (95% CI)** | **k direct, full** | **Primary-only network: estimate (95% CI)** | **k direct, primary-only** | **Direction unchanged** | **Significance unchanged** | **Note** |
| --- | --- | --- | --- | --- | --- | --- | --- | --- | --- |
| Length of stay (days) | CT+Fib vs CT | MD | −1.05 (−3.33 to 1.22) | 12 | −2.61 (−4.92 to −0.31) | 9 | Yes | No |  |
| Length of stay (days) | VATS vs CT | MD | −2.06 (−4.57 to 0.45) | 9 | −3.57 (−6.02 to −1.13) | 8 | Yes | No |  |
| Length of stay (days) | Thora vs CT | MD | 0.18 (−2.45 to 2.81) | 11 | −4.83 (−8.99 to −0.66) | 3 | No | No |  |
| Length of stay (days) | VATS vs CT+Fib | MD | −1.01 (−3.46 to 1.44) | 9 | −0.96 (−3.22 to 1.30) | 9 | Yes | Yes |  |
| Length of stay (days) | Thora vs CT+Fib | MD | 1.23 (−1.80 to 4.27) | 4 | −2.22 (−6.53 to 2.10) | 1 | No | Yes |  |
| Length of stay (days) | Thora vs VATS | MD | 2.24 (−0.90 to 5.38) | 4 | −1.26 (−5.41 to 2.90) | 3 | No | Yes |  |
| Duration of pleural drainage (days) | CT+Fib vs CT | MD | −1.19 (−2.95 to 0.57) | 5 | −2.53 (−4.17 to −0.90) | 3 | Yes | No |  |
| Duration of pleural drainage (days) | VATS vs CT | MD | −2.54 (−4.64 to −0.44) | 3 | −3.74 (−5.49 to −1.98) | 3 | Yes | Yes |  |
| Duration of pleural drainage (days) | Thora vs CT | MD | 0.82 (−2.52 to 4.15) | 3 | Not estimable | 0 | — | — | Not estimable: Thora node absent from primary-only network |
| Duration of pleural drainage (days) | VATS vs CT+Fib | MD | −1.35 (−3.28 to 0.58) | 3 | −1.20 (−2.66 to 0.25) | 3 | Yes | Yes |  |
| Duration of pleural drainage (days) | Thora vs CT+Fib | MD | 2.01 (−1.59 to 5.61) | 1 | Not estimable | 0 | — | — | Not estimable: Thora node absent from primary-only network |
| Duration of pleural drainage (days) | Thora vs VATS | MD | 3.36 (−0.39 to 7.10) | 1 | Not estimable | 0 | — | — | Not estimable: Thora node absent from primary-only network |
| Fever duration (days) | CT+Fib vs CT | MD | −1.02 (−3.02 to 0.97) | 4 | −1.38 (−3.85 to 1.10) | 3 | Yes | Yes |  |
| Fever duration (days) | VATS vs CT | MD | −2.00 (−3.97 to −0.04) | 4 | −2.25 (−4.47 to −0.02) | 4 | Yes | Yes |  |
| Fever duration (days) | Thora vs CT | MD | −0.44 (−3.95 to 3.07) | 2 | Not estimable | 0 | — | — | Not estimable: Thora node absent from primary-only network |
| Fever duration (days) | VATS vs CT+Fib | MD | −0.98 (−2.98 to 1.02) | 4 | −0.87 (−3.14 to 1.40) | 4 | Yes | Yes |  |
| Fever duration (days) | Thora vs CT+Fib | MD | 0.58 (−3.11 to 4.28) | 1 | Not estimable | 0 | — | — | Not estimable: Thora node absent from primary-only network |
| Fever duration (days) | Thora vs VATS | MD | 1.56 (−2.30 to 5.42) | 0 | Not estimable | 0 | — | — | Not estimable: Thora node absent from primary-only network |
| Duration of antibiotics (days) | CT+Fib vs CT | MD | −2.61 (−7.15 to 1.93) | 3 | −2.63 (−7.36 to 2.11) | 3 | Yes | Yes |  |
| Duration of antibiotics (days) | VATS vs CT | MD | −4.95 (−9.36 to −0.54) | 4 | −4.97 (−9.56 to −0.38) | 4 | Yes | Yes |  |
| Duration of antibiotics (days) | Thora vs CT | MD | −3.99 (−10.43 to 2.46) | 2 | −4.22 (−13.29 to 4.86) | 1 | Yes | Yes | Sparse: node k ≤ 3 in primary-only network (k Thora=1, k CT=6) |
| Duration of antibiotics (days) | VATS vs CT+Fib | MD | −2.34 (−7.40 to 2.72) | 2 | −2.34 (−7.56 to 2.87) | 2 | Yes | Yes |  |
| Duration of antibiotics (days) | Thora vs CT+Fib | MD | −1.37 (−8.62 to 5.87) | 1 | −1.59 (−10.83 to 7.65) | 1 | Yes | Yes | Sparse: node k ≤ 3 in primary-only network (k Thora=1, k CTFib=4) |
| Duration of antibiotics (days) | Thora vs VATS | MD | 0.97 (−6.19 to 8.13) | 1 | 0.76 (−8.41 to 9.92) | 1 | Yes | Yes | Sparse: node k ≤ 3 in primary-only network (k Thora=1, k VATS=5) |
| Mortality | CT+Fib vs CT | RR | 0.99 (0.44 to 2.22) | 10 | 0.94 (0.31 to 2.89) | 5 | Yes | Yes |  |
| Mortality | VATS vs CT | RR | 1.29 (0.46 to 3.60) | 8 | 0.80 (0.24 to 2.68) | 7 | No | Yes |  |
| Mortality | Thora vs CT | RR | 2.85 (1.17 to 6.96) | 10 | 2.32 (0.54 to 10.00) | 3 | Yes | No |  |
| Mortality | VATS vs CT+Fib | RR | 1.30 (0.48 to 3.50) | 9 | 0.85 (0.25 to 2.89) | 6 | No | Yes |  |
| Mortality | Thora vs CT+Fib | RR | 2.88 (1.24 to 6.69) | 7 | 2.46 (0.57 to 10.55) | 2 | Yes | No |  |
| Mortality | Thora vs VATS | RR | 2.21 (0.77 to 6.37) | 6 | 2.91 (0.65 to 12.97) | 3 | Yes | Yes |  |
| Complications | CT+Fib vs CT | RR | 1.49 (0.96 to 2.31) | 7 | 1.15 (0.65 to 2.04) | 4 | Yes | Yes |  |
| Complications | VATS vs CT | RR | 1.45 (0.81 to 2.59) | 5 | 0.91 (0.47 to 1.76) | 5 | No | Yes |  |
| Complications | Thora vs CT | RR | 0.75 (0.40 to 1.42) | 6 | 1.46 (0.39 to 5.50) | 2 | No | Yes |  |
| Complications | VATS vs CT+Fib | RR | 0.97 (0.61 to 1.56) | 10 | 0.79 (0.50 to 1.25) | 8 | Yes | Yes |  |
| Complications | Thora vs CT+Fib | RR | 0.50 (0.27 to 0.95) | 3 | 1.27 (0.36 to 4.47) | 1 | No | No |  |
| Complications | Thora vs VATS | RR | 0.52 (0.26 to 1.05) | 3 | 1.61 (0.44 to 5.88) | 2 | No | Yes |  |
| Treatment escalation | CT+Fib vs CT | RR | 0.48 (0.26 to 0.89) | 8 | 0.49 (0.25 to 0.97) | 7 | Yes | Yes |  |
| Treatment escalation | VATS vs CT | RR | 0.17 (0.07 to 0.37) | 7 | 0.15 (0.07 to 0.35) | 7 | Yes | Yes |  |
| Treatment escalation | Thora vs CT | RR | 0.13 (0.04 to 0.36) | 5 | 0.02 (0.003 to 0.12) | 1 | Yes | Yes | Sparse: node k ≤ 3 in primary-only network (k Thora=3, k CT=12) |
| Treatment escalation | VATS vs CT+Fib | RR | 0.35 (0.17 to 0.73) | 9 | 0.31 (0.15 to 0.65) | 9 | Yes | Yes |  |
| Treatment escalation | Thora vs CT+Fib | RR | 0.26 (0.10 to 0.72) | 4 | 0.04 (0.008 to 0.22) | 2 | Yes | Yes | Sparse: node k ≤ 3 in primary-only network (k Thora=3, k CTFib=15) |
| Treatment escalation | Thora vs VATS | RR | 0.76 (0.24 to 2.41) | 2 | 0.13 (0.02 to 0.75) | 2 | Yes | No | Sparse: node k ≤ 3 in primary-only network (k Thora=3, k VATS=15) |
| Treatment success | CT+Fib vs CT | RR | 1.02 (0.93 to 1.12) | 9 | 1.24 (1.07 to 1.44) | 5 | Yes | No |  |
| Treatment success | VATS vs CT | RR | 1.11 (0.99 to 1.24) | 5 | 1.34 (1.13 to 1.58) | 4 | Yes | No |  |
| Treatment success | Thora vs CT | RR | 1.08 (0.97 to 1.20) | 5 | 1.03 (0.82 to 1.30) | 1 | Yes | Yes | Sparse: node k ≤ 3 in primary-only network (k Thora=3, k CT=9) |
| Treatment success | VATS vs CT+Fib | RR | 1.09 (0.99 to 1.19) | 10 | 1.07 (0.95 to 1.22) | 7 | Yes | Yes |  |
| Treatment success | Thora vs CT+Fib | RR | 1.06 (0.95 to 1.18) | 4 | 0.83 (0.66 to 1.04) | 1 | No | Yes | Sparse: node k ≤ 3 in primary-only network (k Thora=3, k CTFib=12) |
| Treatment success | Thora vs VATS | RR | 0.97 (0.86 to 1.10) | 2 | 0.77 (0.61 to 0.98) | 1 | Yes | No | Sparse: node k ≤ 3 in primary-only network (k Thora=3, k VATS=11) |

**Panel B. Network composition and heterogeneity by outcome**

| **Outcome** | **Measure** | **Studies, full** | **Contrasts, full** | **Nodes, full** | **τ², full** | **I², full (%)** | **Studies, primary-only** | **Contrasts, primary-only** | **Nodes, primary-only** | **τ², primary-only** | **I², primary-only (%)** |
| --- | --- | --- | --- | --- | --- | --- | --- | --- | --- | --- | --- |
| Length of stay (days) | MD | 30 | 49 | 4 | 16.35 | 91 | 22 | 33 | 4 (CT, CT+Fib, Thora, VATS) | 11.84 | 90 |
| Duration of pleural drainage (days) | MD | 14 | 16 | 4 | 3.72 | 83 | 9 | 9 | 3 (CT, CT+Fib, VATS) | 1.72 | 78 |
| Fever duration (days) | MD | 11 | 15 | 4 | 4.31 | 86 | 9 | 11 | 3 (CT, CT+Fib, VATS) | 5.38 | 89 |
| Duration of antibiotics (days) | MD | 8 | 13 | 4 | 17.44 | 87 | 7 | 12 | 4 (CT, CT+Fib, Thora, VATS) | 18.72 | 89 |
| Mortality | RR | 27 | 50 | 4 | 0.00 | 0 | 17 | 26 | 4 (CT, CT+Fib, Thora, VATS) | 0.00 | 0 |
| Complications | RR | 24 | 34 | 4 | 0.10 | 16 | 16 | 22 | 4 (CT, CT+Fib, Thora, VATS) | 0.00 | 0 |
| Treatment escalation | RR | 26 | 35 | 4 | 0.66 | 54 | 21 | 28 | 4 (CT, CT+Fib, Thora, VATS) | 0.62 | 50 |
| Treatment success | RR | 24 | 35 | 4 | 0.01 | 80 | 17 | 19 | 4 (CT, CT+Fib, Thora, VATS) | 0.02 | 58 |

*CT, chest tube alone; CT+Fib, chest tube with intrapleural fibrinolytics; VATS, video-assisted thoracoscopic surgery; Thora, thoracotomy; RR, risk ratio; MD, mean difference (days); CI, confidence interval; k direct, number of studies contributing direct evidence to the contrast. Estimates are from the random-effects network model; the first-named treatment is compared with the second (MD < 0 or RR < 1 favors the first-named treatment for length of stay, drainage, fever, antibiotics, mortality, complications and escalation; RR > 1 favors the first-named treatment for treatment success). Values are rounded half-up to two decimal places (three for risk-ratio lower bounds below 0.01). Full-network estimates are reproduced as reported in the main text. The primary-only network was restricted to study-by-treatment units classified as primary-line (Supplementary Table S11); mixed, step-up/rescue and unclear units were excluded. Direction unchanged = point estimate on the same side of the null in both networks; significance unchanged = 95% CI excludes or includes the null in both networks. Sparse = at least one node contributed three or fewer studies in the primary-only network.*

**Supplementary Table S9. Distribution of potential effect modifiers across the six direct comparisons in the principal network (transitivity assessment): study design, publication period, setting, WHO region, reporting of empyema stage, and treatment line of contributing arms by node.**

| **Characteristic** | **CT vs CT+Fib** | **CT vs VATS** | **CT vs Thora** | **CT+Fib vs VATS** | **CT+Fib vs Thora** | **VATS vs Thora** |
| --- | --- | --- | --- | --- | --- | --- |
| Studies in the direct comparison, k | 15 | 12 | 14 | 15 | 8 | 8 |
| **Study design, n** | | | | | | |
| Randomized trial | 0 | 0 | 0 | 4 | 0 | 0 |
| Prospective cohort | 2 | 2 | 4 | 1 | 0 | 2 |
| Retrospective cohort | 13 | 9 | 10 | 10 | 8 | 6 |
| Case series | 0 | 1 | 0 | 0 | 0 | 0 |
| **Publication period** | | | | | | |
| Median year (range) | 2013 (2004-2025) | 2009 (2000-2020) | 2009 (2003-2019) | 2014 (2000-2026) | 2009 (2004-2016) | 2007 (2005-2016) |
| **Setting, n** | | | | | | |
| Single-center | 12 | 11 | 12 | 12 | 6 | 7 |
| Multicenter | 3 | 1 | 2 | 3 | 2 | 1 |
| **WHO region, n** | | | | | | |
| Europe | 8 | 6 | 5 | 9 | 6 | 4 |
| South-East Asia | 5 | 2 | 5 | 2 | 1 | 2 |
| Americas | 0 | 3 | 2 | 3 | 0 | 1 |
| Western Pacific | 1 | 1 | 1 | 1 | 1 | 1 |
| Eastern Mediterranean | 0 | 0 | 1 | 0 | 0 | 0 |
| Africa | 1 | 0 | 0 | 0 | 0 | 0 |
| **Reporting of empyema stage** | | | | | | |
| Stage reported, n (%) | 3 (20%) | 3 (25%) | 2 (14%) | 6 (40%) | 2 (25%) | 2 (25%) |
| **Treatment line of contributing arms, by node** | | | | | | |
| **CT alone arms, n** | 15 | 12 | 14 | — | — | — |
| Primary, n (%) | 13 (87%) | 12 (100%) | 13 (93%) | — | — | — |
| Step-up/rescue, n (%) | 0 (0%) | 0 (0%) | 0 (0%) | — | — | — |
| Mixed, n | 0 | 0 | 0 | — | — | — |
| Unclear, n | 2 | 0 | 1 | — | — | — |
| **CT+Fib arms, n** | 15 | — | — | 15 | 8 | — |
| Primary, n (%) | 9 (60%) | — | — | 12 (80%) | 3 (38%) | — |
| Step-up/rescue, n (%) | 4 (27%) | — | — | 3 (20%) | 4 (50%) | — |
| Mixed, n | 0 | — | — | 0 | 0 | — |
| Unclear, n | 2 | — | — | 0 | 1 | — |
| **VATS arms, n** | — | 12 | — | 15 | — | 8 |
| Primary, n (%) | — | 9 (75%) | — | 11 (73%) | — | 5 (62%) |
| Step-up/rescue, n (%) | — | 2 (17%) | — | 3 (20%) | — | 2 (25%) |
| Mixed, n | — | 1 | — | 1 | — | 1 |
| Unclear, n | — | 0 | — | 0 | — | 0 |
| **Thoracotomy arms, n** | — | — | 14 | — | 8 | 8 |
| Primary, n (%) | — | — | 3 (21%) | — | 3 (38%) | 3 (38%) |
| Step-up/rescue, n (%) | — | — | 8 (57%) | — | 3 (38%) | 4 (50%) |
| Mixed, n | — | — | 2 | — | 1 | 1 |
| Unclear, n | — | — | 1 | — | 1 | 0 |

*CT, chest tube alone; CT+Fib, chest tube with intrapleural fibrinolytics; VATS, video-assisted thoracoscopic surgery; Thora, thoracotomy; k, number of studies contributing direct evidence to the comparison. Treatment line follows the study-by-treatment unit classification in Supplementary Table S11 (primary; step-up/rescue; mixed = node pools primary and step-up patients that cannot be separated; unclear). Percentages are of the arms contributing to that node in that comparison. — = node not part of the comparison. WHO region was assigned from the country of the study population as follows: Argentina, Americas; Australia, Western Pacific; Belgium, Europe; Brazil, Americas; Canada, Americas; China, Western Pacific; Croatia, Europe; Finland, Europe; Germany, Europe; India, South-East Asia; Iran, Eastern Mediterranean; Israel, Europe; Italy, Europe; Nepal, South-East Asia; Poland, Europe; Portugal, Europe; Saudi Arabia, Eastern Mediterranean; South Africa, Africa; Spain, Europe; Taiwan, Western Pacific; Turkey, Europe; USA, Americas; United Kingdom, Europe.*

**Supplementary Table S10. Study-level accounting of the 77 included studies by design, contribution to the analyses and risk-of-bias instrument.** Kercher 2000 was appraised with both ROBINS-I and JBI and is counted once as a study and twice as an appraisal (78 appraisals of 77 studies).

**Panel A. Cross-tabulation of study design, contribution to the analyses and risk-of-bias instrument (number of studies)**

| **Study design** | **Contribution to the analyses** | **JBI** | **JBI + ROBINS-I** | **ROBINS-I** | **RoB 2** | **Total** |
| --- | --- | --- | --- | --- | --- | --- |
| Comparative observational cohort | Network (>=2 arms) | 0 | 0 | 35 | 0 | 35 |
| Comparative observational cohort | Not in network: single-arm data only (descriptive synthesis; no pairwise contrast) | 0 | 0 | 33 | 0 | 33 |
| RCT | Network (>=2 arms) | 0 | 0 | 0 | 4 | 4 |
| RCT | Not in network: single-arm data only (descriptive synthesis; no pairwise contrast) | 0 | 0 | 0 | 1 | 1 |
| Single-arm case series | Network (>=2 arms) | 0 | 1 | 0 | 0 | 1 |
| Single-arm case series | Not in network: single-arm data only (descriptive synthesis; no pairwise contrast) | 3 | 0 | 0 | 0 | 3 |
| **Total** | All studies | 3 | 1 | 68 | 5 | 77 |

**Panel B. Appraisal totals**

| **Instrument** | **Appraisals, n** |
| --- | --- |
| ROBINS-I | 69 |
| RoB 2 | 5 |
| JBI | 4 |
| Total appraisals | 78 |
| Distinct studies | 77 |

**Panel C. Contribution totals**

| **Contribution to the analyses** | **Studies, n** |
| --- | --- |
| Network (>=2 arms) | 40 |
| Pairwise-only (2 arms but not in network) | 0 |
| Not in network (single-arm) | 37 |
| Total studies | 77 |

*Kercher 2000 is a case series with two treatment groups; it was appraised with both ROBINS-I (for the comparative contrast) and JBI (for its case-series design) and is counted once as a study, twice as an appraisal. 'Pairwise only' is 0 because every study with >=2 network-node arms for any outcome enters the network; the pairwise meta-analyses use the same contrasts. The 5 RoB 2 studies: 4 contribute to the network; Livingston 2020 (RCT) contributed only single-arm data because its comparator was not a network node. Design classes follow Supplementary Table S2 (RCT; prospective/retrospective cohort = comparative observational cohort; case series).*

**Supplementary Table S11. Treatment-line classification of every study-by-treatment unit (primary, step-up/rescue, mixed, unclear) with the basis for each decision; units marked in_network = TRUE entered the principal network.**

| **Study** | **Node** | **In network** | **Final treatment line** | **Basis for decision** |
| --- | --- | --- | --- | --- |
| Agrawal 2021 (21) | Thora | FALSE | Primary | All 2 contributing arms were coded primary (initial pleural intervention) in the extraction record. |
| Alexiou 2003 (22) | Thora | FALSE | Step-up/Rescue | The single contributing arm was coded step-up (delivered after failure of a less invasive strategy) in the extraction record. |
| Almaramhy 2015 (23) | CT | TRUE | Primary | The single contributing arm was coded primary (initial pleural intervention) in the extraction record. |
| Almaramhy 2015 (23) | Thora | TRUE | Step-up/Rescue | The single contributing arm was coded step-up (delivered after failure of a less invasive strategy) in the extraction record. |
| Ateş 2023 (24) | VATS | FALSE | Primary | All 2 contributing arms were coded primary (initial pleural intervention) in the extraction record. |
| Aydoğan 2008 (25) | CT | TRUE | Primary | The single contributing arm was coded primary (initial pleural intervention) in the extraction record. |
| Aydoğan 2008 (25) | CT+Fib | TRUE | Primary | The single contributing arm was coded primary (initial pleural intervention) in the extraction record. |
| Bailey 2005 (26) | CT | TRUE | Primary | The single contributing arm was coded primary (initial pleural intervention) in the extraction record. |
| Bailey 2005 (26) | Thora | TRUE | Primary | The single contributing arm was coded primary (initial pleural intervention) in the extraction record. |
| Bailey 2005 (26) | VATS | TRUE | Primary | The single contributing arm was coded primary (initial pleural intervention) in the extraction record. |
| Baranwal 2003 (27) | CT | TRUE | Primary | All 2 contributing arms were coded primary (initial pleural intervention) in the extraction record. |
| Baranwal 2003 (27) | Thora | TRUE | Step-up/Rescue | All 2 contributing arms were coded step-up (delivered after failure of a less invasive strategy) in the extraction record. |
| Barglik 2021 (28) | VATS | FALSE | Step-up/Rescue | The single contributing arm was coded step-up (delivered after failure of a less invasive strategy) in the extraction record. |
| Chen 2002 (31) | CT | FALSE | Primary | The single contributing arm was coded primary (initial pleural intervention) in the extraction record. |
| Chen 2009 (30) | VATS | FALSE | Primary | The single contributing arm was coded primary (initial pleural intervention) in the extraction record. |
| Chiu 2006 (32) | CT | TRUE | Primary | The single contributing arm was coded primary (initial pleural intervention) in the extraction record. |
| Chiu 2006 (32) | CT+Fib | TRUE | Primary | The single contributing arm was coded primary (initial pleural intervention) in the extraction record. |
| Chiu 2006 (32) | Thora | TRUE | Step-up/Rescue | The single contributing arm was coded rescue (delivered for clinical deterioration) in the extraction record. |
| Chiu 2006 (32) | VATS | TRUE | Primary | The single contributing arm was coded primary (initial pleural intervention) in the extraction record. |
| Cobanoglu 2011 (33) | CT+Fib | TRUE | Primary | The single contributing arm was coded primary (initial pleural intervention) in the extraction record. |
| Cobanoglu 2011 (33) | VATS | TRUE | Primary | The single contributing arm was coded primary (initial pleural intervention) in the extraction record. |
| Cohen 2003 (34) | CT | TRUE | Primary | The single contributing arm was coded primary (initial pleural intervention) in the extraction record. |
| Cohen 2003 (34) | VATS | TRUE | Primary | The single contributing arm was coded primary (initial pleural intervention) in the extraction record. |
| Darpan Jakkal 2024 (35) | CT+Fib | TRUE | Primary | The single contributing arm was coded primary (initial pleural intervention) in the extraction record. |
| Darpan Jakkal 2024 (35) | VATS | TRUE | Primary | The single contributing arm was coded primary (initial pleural intervention) in the extraction record. |
| Dass 2011 (36) | CT | TRUE | Primary | The single contributing arm was coded primary (initial pleural intervention) in the extraction record. |
| Dass 2011 (36) | CT+Fib | TRUE | Step-up/Rescue | The single contributing arm was coded step-up (delivered after failure of a less invasive strategy) in the extraction record. |
| Dass 2011 (36) | Thora | TRUE | Step-up/Rescue | The single contributing arm was coded rescue (delivered for clinical deterioration) in the extraction record. |
| De Benedictis 2019 (37) | CT | TRUE | Primary | The single contributing arm was coded primary (initial pleural intervention) in the extraction record. |
| De Benedictis 2019 (37) | VATS | TRUE | Primary | The single contributing arm was coded primary (initial pleural intervention) in the extraction record. |
| Demirhan 2008 (38) | CT | TRUE | Primary | The single contributing arm was coded primary (initial pleural intervention) in the extraction record. |
| Demirhan 2008 (38) | CT+Fib | TRUE | Step-up/Rescue | The single contributing arm was coded step-up (delivered after failure of a less invasive strategy) in the extraction record. |
| Demirhan 2008 (38) | Thora | TRUE | Mixed | The node pools arms coded primary (n = 17) and step-up (n = 110); these patients cannot be separated in the analysis, so the unit is classified as mixed rather than primary. |
| Demirhan 2008 (38) | VATS | TRUE | Step-up/Rescue | The single contributing arm was coded step-up (delivered after failure of a less invasive strategy) in the extraction record. |
| Ekingen 2004 (39) | CT+Fib | FALSE | Primary | All 2 contributing arms were coded primary (initial pleural intervention) in the extraction record. |
| Eroğlu E 2004 (40) | Thora | FALSE | Primary | The single contributing arm was coded primary (initial pleural intervention) in the extraction record. |
| Espinosa 2016 (41) | VATS | FALSE | Primary | The single contributing arm was coded primary (initial pleural intervention) in the extraction record. |
| Gasior 2013 (42) | CT+Fib | FALSE | Primary | The single contributing arm was coded primary (initial pleural intervention) in the extraction record. |
| Gayretli-Aydın 2016 (43) | CT | TRUE | Primary | The single contributing arm was coded primary (initial pleural intervention) in the extraction record. |
| Gayretli-Aydın 2016 (43) | CT+Fib | TRUE | Step-up/Rescue | The single contributing arm was coded step-up (delivered after failure of a less invasive strategy) in the extraction record. |
| Gayretli-Aydın 2016 (43) | Thora | TRUE | Step-up/Rescue | The single contributing arm was coded step-up (delivered after failure of a less invasive strategy) in the extraction record. |
| Gayretli-Aydın 2016 (43) | VATS | TRUE | Step-up/Rescue | The single contributing arm was coded step-up (delivered after failure of a less invasive strategy) in the extraction record. |
| Goyal 2014 (44) | CT | TRUE | Primary | The single contributing arm was coded primary (initial pleural intervention) in the extraction record. |
| Goyal 2014 (44) | Thora | TRUE | Step-up/Rescue | The single contributing arm was coded rescue (delivered for clinical deterioration) in the extraction record. |
| Grisaru-Soen 2013 (45) | CT | TRUE | Unclear | Treatment line was not recorded in the extraction record; the arm labels in the report (chest tube alone, chest tube with fibrinolytics, VATS decortication) do not state treatment line and the full text could not be obtained for re-examination, so the unit is classified as unclear. |
| Grisaru-Soen 2013 (45) | CT+Fib | TRUE | Unclear | Treatment line was not recorded in the extraction record; the arm labels in the report (chest tube alone, chest tube with fibrinolytics, VATS decortication) do not state treatment line and the full text could not be obtained for re-examination, so the unit is classified as unclear. |
| Grisaru-Soen 2013 (45) | Thora | TRUE | Unclear | Treatment line was not recorded in the extraction record; the arm labels in the report (chest tube alone, chest tube with fibrinolytics, VATS decortication) do not state treatment line and the full text could not be obtained for re-examination, so the unit is classified as unclear. |
| Haapanen 2026 (46) | CT+Fib | TRUE | Primary | The single contributing arm was coded primary (initial pleural intervention) in the extraction record. |
| Haapanen 2026 (46) | VATS | TRUE | Primary | The single contributing arm was coded primary (initial pleural intervention) in the extraction record. |
| Huang 2021 (47) | VATS | FALSE | Step-up/Rescue | The single contributing arm was coded step-up (delivered after failure of a less invasive strategy) in the extraction record. |
| Jaiswal 2020 (48) | Thora | FALSE | Step-up/Rescue | The single contributing arm was coded step-up (delivered after failure of a less invasive strategy) in the extraction record. |
| Kercher 2000 (49) | CT | TRUE | Primary | The single contributing arm was coded primary (initial pleural intervention) in the extraction record. |
| Kercher 2000 (49) | VATS | TRUE | Primary | The single contributing arm was coded primary (initial pleural intervention) in the extraction record. |
| Khalil 2007 (50) | CT | TRUE | Primary | The single contributing arm was coded primary (initial pleural intervention) in the extraction record. |
| Khalil 2007 (50) | CT+Fib | TRUE | Primary | The single contributing arm was coded primary (initial pleural intervention) in the extraction record. |
| Khalil 2007 (50) | Thora | TRUE | Primary | The single contributing arm was coded primary (initial pleural intervention) in the extraction record. |
| Khalil 2007 (50) | VATS | TRUE | Primary | The single contributing arm was coded primary (initial pleural intervention) in the extraction record. |
| Knebel 2018 (52) | VATS | FALSE | Mixed | The node pools arms coded primary (n = 60) and step-up (n = 19); these patients cannot be separated in the analysis, so the unit is classified as mixed rather than primary. |
| Knudtson 2004 (53) | VATS | FALSE | Primary | The single contributing arm was coded primary (initial pleural intervention) in the extraction record. |
| Kumar 2013 (54) | CT | FALSE | Primary | Treatment line was not recorded in the extraction record; the full text states that all children received injectable antibiotics and chest tube drainage as initial management, so the unit is classified as primary. |
| Kumar 2022 (55) | CT | FALSE | Primary | All 2 contributing arms were coded primary (initial pleural intervention) in the extraction record. |
| Kılıç 2002 (51) | CT+Fib | FALSE | Primary | The single contributing arm was coded primary (initial pleural intervention) in the extraction record. |
| Lamas-Pinheiro 2016 (56) | VATS | FALSE | Primary | All 2 contributing arms were coded primary (initial pleural intervention) in the extraction record. |
| Livingston 2016 (57) | CT+Fib | TRUE | Primary | The single contributing arm was coded primary (initial pleural intervention) in the extraction record. |
| Livingston 2016 (57) | VATS | TRUE | Primary | The single contributing arm was coded primary (initial pleural intervention) in the extraction record. |
| Livingston 2020 (58) | CT+Fib | FALSE | Step-up/Rescue | The single contributing arm was coded step-up (delivered after failure of a less invasive strategy) in the extraction record. |
| Maffey 2019 (59) | CT | TRUE | Primary | The single contributing arm was coded primary (initial pleural intervention) in the extraction record. |
| Maffey 2019 (59) | Thora | TRUE | Step-up/Rescue | The single contributing arm was coded step-up (delivered after failure of a less invasive strategy) in the extraction record. |
| Marhuenda 2014 (60) | CT+Fib | TRUE | Primary | The single contributing arm was coded primary (initial pleural intervention) in the extraction record. |
| Marhuenda 2014 (60) | VATS | TRUE | Primary | The single contributing arm was coded primary (initial pleural intervention) in the extraction record. |
| Mathew 2018 (61) | CT | TRUE | Primary | The single contributing arm was coded primary (initial pleural intervention) in the extraction record. |
| Mathew 2018 (61) | CT+Fib | TRUE | Primary | The single contributing arm was coded primary (initial pleural intervention) in the extraction record. |
| Meier 2000 (62) | CT+Fib | TRUE | Primary | The single contributing arm was coded primary (initial pleural intervention) in the extraction record. |
| Meier 2000 (62) | VATS | TRUE | Mixed | The node pools arms coded primary (n = 4) and step-up (n = 4); these patients cannot be separated in the analysis, so the unit is classified as mixed rather than primary. |
| Mondal 2025 (63) | CT | TRUE | Primary | The single contributing arm was coded primary (initial pleural intervention) in the extraction record. |
| Mondal 2025 (63) | CT+Fib | TRUE | Primary | The single contributing arm was coded primary (initial pleural intervention) in the extraction record. |
| Nandan 2019 (64) | CT | TRUE | Primary | The single contributing arm was coded primary (initial pleural intervention) in the extraction record. |
| Nandan 2019 (64) | CT+Fib | TRUE | Primary | The single contributing arm was coded primary (initial pleural intervention) in the extraction record. |
| Narayanappa 2013 (65) | CT | TRUE | Primary | The single contributing arm was coded primary (initial pleural intervention) in the extraction record. |
| Narayanappa 2013 (65) | Thora | TRUE | Step-up/Rescue | All 2 contributing arms were coded step-up (delivered after failure of a less invasive strategy) in the extraction record. |
| Narayanappa 2013 (65) | VATS | TRUE | Mixed | The node pools arms coded primary (n = 4) and step-up (n = 5); these patients cannot be separated in the analysis, so the unit is classified as mixed rather than primary. |
| Ozcelik 2003 (67) | CT+Fib | FALSE | Primary | The single contributing arm was coded primary (initial pleural intervention) in the extraction record. |
| Ozcelik 2004 (66) | CT | TRUE | Primary | The single contributing arm was coded primary (initial pleural intervention) in the extraction record. |
| Ozcelik 2004 (66) | CT+Fib | TRUE | Step-up/Rescue | The single contributing arm was coded step-up (delivered after failure of a less invasive strategy) in the extraction record. |
| Ozcelik 2004 (66) | Thora | TRUE | Primary | The single contributing arm was coded primary (initial pleural intervention) in the extraction record. |
| Pandey 2015 (68) | Thora | TRUE | Step-up/Rescue | The single contributing arm was coded step-up (delivered after failure of a less invasive strategy) in the extraction record. |
| Pandey 2015 (68) | VATS | TRUE | Primary | The single contributing arm was coded primary (initial pleural intervention) in the extraction record. |
| Pappalardo 2009 (69) | VATS | FALSE | Primary | The single contributing arm was coded primary (initial pleural intervention) in the extraction record. |
| Parelkar 2017 (60) | VATS | FALSE | Step-up/Rescue | The single contributing arm was coded step-up (delivered after failure of a less invasive strategy) in the extraction record. |
| Pereira 2017 (71) | VATS | FALSE | Primary | All 2 contributing arms were coded primary (initial pleural intervention) in the extraction record. |
| Plackett 2010 (72) | CT | TRUE | Primary | The single contributing arm was coded primary (initial pleural intervention) in the extraction record. |
| Plackett 2010 (72) | VATS | TRUE | Primary | The single contributing arm was coded primary (initial pleural intervention) in the extraction record. |
| Pogorelic 2021 (73) | VATS | FALSE | Primary | The single contributing arm was coded primary (initial pleural intervention) in the extraction record. |
| Proesmans 2013 (74) | CT | TRUE | Primary | The single contributing arm was coded primary (initial pleural intervention) in the extraction record. |
| Proesmans 2013 (74) | CT+Fib | TRUE | Primary | The single contributing arm was coded primary (initial pleural intervention) in the extraction record. |
| Proesmans 2013 (74) | VATS | TRUE | Primary | The single contributing arm was coded primary (initial pleural intervention) in the extraction record. |
| Ranjan Kunar 2024 (75) | CT | TRUE | Primary | The single contributing arm was coded primary (initial pleural intervention) in the extraction record. |
| Ranjan Kunar 2024 (75) | CT+Fib | TRUE | Primary | The single contributing arm was coded primary (initial pleural intervention) in the extraction record. |
| Ratta 2023 (76) | CT+Fib | TRUE | Primary | All 2 contributing arms were coded primary (initial pleural intervention) in the extraction record. |
| Ratta 2023 (76) | VATS | TRUE | Primary | All 2 contributing arms were coded primary (initial pleural intervention) in the extraction record. |
| Roxburgh 2007 (77) | Thora | TRUE | Primary | The single contributing arm was coded primary (initial pleural intervention) in the extraction record. |
| Roxburgh 2007 (77) | VATS | TRUE | Primary | The single contributing arm was coded primary (initial pleural intervention) in the extraction record. |
| Sahin 2013 (78) | CT+Fib | TRUE | Primary | The single contributing arm was coded primary (initial pleural intervention) in the extraction record. |
| Sahin 2013 (78) | Thora | TRUE | Primary | The single contributing arm was coded primary (initial pleural intervention) in the extraction record. |
| Sarafi 2023 (79) | CT+Fib | FALSE | Step-up/Rescue | The single contributing arm was coded rescue (delivered for clinical deterioration) in the extraction record. |
| Satish 2003 (80) | CT | FALSE | Primary | The single contributing arm was coded primary (initial pleural intervention) in the extraction record. |
| Satpathy 2005 (81) | CT | TRUE | Primary | Treatment line was not recorded in the extraction record; the full text states that all cases were treated with chest tube drainage as initial therapy, so the unit is classified as primary. |
| Satpathy 2005 (81) | Thora | TRUE | Mixed | Treatment line was not recorded in the extraction record; the full text reports 5 thoracotomies, 2 performed as primary treatment for multiloculated disease and 3 as salvage after failed chest tube drainage, so the pooled node is predominantly rescue and is classified as mixed rather than primary. |
| Schultz 2004 (82) | VATS | FALSE | Primary | All 2 contributing arms were coded primary (initial pleural intervention) in the extraction record. |
| Segerer 2017 (83) | CT | FALSE | Primary | The single contributing arm was coded primary (initial pleural intervention) in the extraction record. |
| Segerer 2017 (83) | CT+Fib | TRUE | Primary | The single contributing arm was coded primary (initial pleural intervention) in the extraction record. |
| Segerer 2017 (83) | VATS | TRUE | Primary | The single contributing arm was coded primary (initial pleural intervention) in the extraction record. |
| Sehitogullari 2019 (7) | CT | TRUE | Unclear | No chest tube or chest tube with fibrinolytics arm is recorded in the extraction record (the only recorded arm is thoracocentesis with antibiotics), so the treatment line of this unit could not be determined and it is classified as unclear. |
| Sehitogullari 2019 (7) | CT+Fib | TRUE | Unclear | No chest tube or chest tube with fibrinolytics arm is recorded in the extraction record (the only recorded arm is thoracocentesis with antibiotics), so the treatment line of this unit could not be determined and it is classified as unclear. |
| Shankar 2000 (85) | Thora | FALSE | Primary | The single contributing arm was coded primary (initial pleural intervention) in the extraction record. |
| Shankar 2020 (84) | CT+Fib | TRUE | Step-up/Rescue | The single contributing arm was coded step-up (delivered after failure of a less invasive strategy) in the extraction record. |
| Shankar 2020 (84) | VATS | TRUE | Step-up/Rescue | The single contributing arm was coded step-up (delivered after failure of a less invasive strategy) in the extraction record. |
| Shoseyov 2002 (86) | CT | FALSE | Primary | The single contributing arm was coded primary (initial pleural intervention) in the extraction record. |
| Singh 2018 (87) | Thora | FALSE | Primary | The single contributing arm was coded primary (initial pleural intervention) in the extraction record. |
| Singh 2020 (88) | CT | TRUE | Primary | The single contributing arm was coded primary (initial pleural intervention) in the extraction record. |
| Singh 2020 (88) | VATS | TRUE | Primary | The single contributing arm was coded primary (initial pleural intervention) in the extraction record. |
| St. Peter 2008 (89) | CT+Fib | TRUE | Primary | The single contributing arm was coded primary (initial pleural intervention) in the extraction record. |
| St. Peter 2008 (89) | VATS | TRUE | Primary | The single contributing arm was coded primary (initial pleural intervention) in the extraction record. |
| Stefanutti 2010 (90) | CT+Fib | FALSE | Primary | The single contributing arm was coded primary (initial pleural intervention) in the extraction record. |
| Suchar 2006 (91) | VATS | FALSE | Primary | The single contributing arm was coded primary (initial pleural intervention) in the extraction record. |
| Tander 2007 (92) | VATS | FALSE | Primary | The single contributing arm was coded primary (initial pleural intervention) in the extraction record. |
| Ulku 2004 (93) | CT+Fib | FALSE | Primary | All 2 contributing arms were coded primary (initial pleural intervention) in the extraction record. |
| Van Loo 2014 (94) | CT+Fib | FALSE | Primary | The single contributing arm was coded primary (initial pleural intervention) in the extraction record. |
| Yue 2019 (95) | CT | FALSE | Primary | The single contributing arm was coded primary (initial pleural intervention) in the extraction record. |
| Zampoli 2015 (96) | CT | TRUE | Primary | The single contributing arm was coded primary (initial pleural intervention) in the extraction record. |
| Zampoli 2015 (96) | CT+Fib | TRUE | Primary | The single contributing arm was coded primary (initial pleural intervention) in the extraction record. |

*CT, chest tube alone; CT+Fib, chest tube with intrapleural fibrinolytics; VATS, video-assisted thoracoscopic surgery; Thora, thoracotomy. Studies are identified as in Supplementary Table S2 (first author, year, reference number). One row per study-by-treatment unit (a study contributing two treatments appears twice). In network = TRUE indicates that the unit contributed to at least one contrast in the principal network; units marked FALSE contributed single-arm data only. Final treatment line: Primary = all contributing arms coded as primary therapy; Step-up/Rescue = all contributing arms delivered after failure of a prior intervention or for clinical deterioration; Mixed = node pools primary and step-up patients that cannot be separated; Unclear = treatment line not determinable from the report. Basis for decision states the source of the classification: the arm-level coding in the data-extraction record, or, where that coding was absent, a re-reading of the full text. Only units classified as Primary entered the primary-treatment-only sensitivity network (Supplementary Table S8, Supplementary Figure S27).*
